# Global analysis of constraints to natural climate solution implementation

**DOI:** 10.1093/pnasnexus/pgaf173

**Published:** 2025-06-24

**Authors:** Hilary Brumberg, Margaret Hegwood, Waverly Eichhorst, Anna LoPresti, James T Erbaugh, Timm Kroeger

**Affiliations:** Department of Environmental Studies, University of Colorado Boulder, Boulder, CO 80303, USA; Emmett Interdisciplinary Program in Environment and Resources, Doerr School of Sustainability, Stanford University, Stanford, CA 94305, USA; The Natural Capital Project, Stanford University, Stanford, CA 94305, USA; Department of Environmental Studies, University of Colorado Boulder, Boulder, CO 80303, USA; Cooperative Institute for Research in Environmental Science, Boulder, CO 80309, USA; Department of Environmental Studies, University of Colorado Boulder, Boulder, CO 80303, USA; Department of Ecology and Evolutionary Biology, University of Colorado Boulder, Boulder, CO 80302, USA; Global Science, The Nature Conservancy, Arlington, VA 22203, USA; Department of Environmental Studies, Dartmouth College, Hanover, NH 03755, USA; Global Science, The Nature Conservancy, Arlington, VA 22203, USA

**Keywords:** nature-based climate solutions, climate change, Sustainable Development Goals, spatial analysis, conservation

## Abstract

Natural climate solutions (NCS) could provide over one-third of the climate mitigation needed between now and 2030 to limit warming below 2°C and support the Sustainable Development Goals. However, large disparities persist between the estimated biophysical climate mitigation potential (CMP) of NCS and their actual implementation. Social, political, informational, and economic factors contribute to this gap, but the spatial distribution of these constraints and their impacts on different NCS pathways remains poorly understood. Understanding these constraints is especially important due to the large uncertainties in NCS CMP and growing research on spatial prioritization of NCS, often based only on biophysical criteria. We identified and mapped nonbiophysical constraints to NCS implementation efficacy by conducting a systematic review of recent peer-reviewed literature across 10 high-CMP NCS pathways. From 1,821 papers, we identified 352 that provided 2,480 observations of 39 unique constraints from 135 countries. We mapped the spatial distribution of these constraints and analyzed patterns across NCS pathways and geographic classifications. Lack of funding, insufficient information on NCS management, and ineffective policies emerged as the most common constraints globally. However, each pathway and geography faced a distinct suite of interrelated constraints spanning multiple categories. These findings highlight the need for context-specific, equitable solutions, likely requiring transdisciplinary approaches and cross-sectoral collaborations. The results could also help increase accuracy of NCS CMP estimates. We discuss how adaptive management may be used for NCS initiatives at any scale to proactively diagnose co-occurring constraints at each implementation phase and to develop integrated, place-based solutions.

**Significance Statement**

This study presents the first global, country-level analysis of a comprehensive set of constraints on natural climate solution (NCS) implementation. Previous studies either reported NCS enabling factors, were nonspatial, or examined a small set of broad feasibility indicators. We identified 2,480 instances of 39 unique constraints across 135 countries to map factors contributing to the critical gap between the biophysical potential of NCS and their actual implementation. While lack of funding was the most observed constraint, each geography and NCS pathway faced a unique suite of co-occurring and interrelated constraints, highlighting the necessity of context-specific, integrated solutions. Our results and open source database can inform international NCS policy, funding decisions, and accuracy of NCS climate mitigation potential estimates. We discuss how adaptive management may be used to systematically diagnose co-occurring constraints and develop proactive, holistic solutions for NCS initiatives of any scale.

## Introduction

Natural climate solutions (NCS) are conservation, restoration, and improved management practices in terrestrial or aquatic systems that reduce greenhouse gas emissions (GHG) or increase carbon dioxide sequestration, with no net negative impact on food and fiber supply or biodiversity, when implemented in socially and culturally responsible ways (1, 2). If effectively implemented on a global scale, NCS have the potential to provide over one-third of the climate mitigation needed to stabilize warming to below 2°C by 2030 (1) and help achieve multiple Sustainable Development Goals (SDGs) (3, 4). More than 80% of revised climate change commitments under the Paris Agreement (nationally determined contributions, NDCs) include nature-based climate solutions, which include the majority of NCS actions (2, 5). NCS are prominently featured in Aichi Biodiversity Targets (6), the 30 × 30 target under the Convention on Biological Diversity (6), climate initiatives from corporations and financial institutions (5), and multinational forest landscape restoration initiatives (4, 6, 7). However, the limited data available on progress towards NCS commitments suggests that on-the-ground implementation of NCS is falling short of these targets (6, 8, 9). Uncertainties remain about the realization of potential benefits from NCS activities due to a lack of information on factors that may contribute to an “implementation gap,” or a gap between targets and outcomes (3, 6, 10).

The identification of priority areas for NCS is often guided by biophysical criteria, such as carbon sequestration and GHG emission reduction potential (hereafter jointly referred to as climate mitigation potential [CMP]), biodiversity conservation priorities, and the maximization of ecosystem service provisioning, rather than considerations of NCS implementation feasibility, leading to high uncertainty in CMP estimates (1, 5, 11–17). Factors that affect the feasibility of NCS implementation may include biophysical conditions as well as constraints posed by policy, technology, economic, organizational, and governance factors (2, 13, 18–22). Constraints to implementing NCS may occur at various phases of project design and implementation. These constraints may decrease the likelihood of successful implementation of NCS or their efficacy in mitigating greenhouse gases; they may also increase risks of impermanence or leakage (5, 20). For instance, Zeng et al. (23) found that only 0.3–18% of the biophysical CMP of reforestation in Southeast Asia may be feasible when considering operational, financial, and land use constraints. More work is needed to identify NCS implementation constraints to improve estimates of the potential for NCS to effectively contribute to climate change mitigation, biodiversity conservation, and SDGs (2).

The presence and magnitude of nonbiophysical constraints to NCS implementation efficacy often vary with local context (6, 7, 20). Local socioeconomic, political, and environmental contexts—including political stability, cultural attitudes toward conservation, economic dependence on natural resources, levels of existing degradation, vulnerability to climate change, and legal and regulatory frameworks—influence the constraints faced in different geographies (6, 7, 22, 24). NCS projects that fail to integrate local contexts or meaningfully engage local actors, rightsholders, and other stakeholders may result in inequitable outcomes and other undesirable consequences (25–28). In such cases, tradeoffs between NCS outcomes and community priorities can arise (13, 22, 27). Moreover, as CMP is distributed unevenly around the world (1, 12), overcoming constraints in locations with high CMP may provide greater climate change mitigation.

Additionally, different NCS pathways, such as reforestation, agroforestry, and avoided grassland conversion, face different constraints (1, 6, 7, 12, 22). Pathways may vary in their complexity of implementation and monitoring, attractiveness to stakeholders, cultural acceptance, perceived or actual risks, and socioeconomic benefits (6, 7, 22). For instance, pathways that provide tangible co-benefits such as fruit from agroforestry may be more attractive to some stakeholders (29). However, these pathways often require a high degree of technical expertise to implement because they involve complex land-use changes and management practices (30). Moreover, different ecosystem types vary in their CMP and may face different implementation challenges due to local socio-ecological conditions (1, 6, 7, 12).

More detailed and spatially explicit information is needed on NCS constraints for at least three reasons. First, such information can facilitate closing the “implementation gap” through the development of interventions that overcome place-based constraints by better directing resources to on-the-ground challenges experienced by stakeholders during NCS implementation (7, 24). Second, understanding constraints can mitigate inequitable outcomes by identifying the conditions under which NCS meet the goal of resulting in no net negative impact on social, cultural, and biodiversity goals—and the conditions under which they do not (2). Third, spatially explicit information about the feasibility of NCS implementation can inform more accurate estimates of the CMP that could be achieved through NCS and to provide a better understanding of how much NCS can likely contribute to each country's NDC (6, 7, 14, 20, 24).

We conducted the most comprehensive and spatially explicit analysis to date on reported constraints to NCS implementation globally across 10 major NCS pathways: agroforestry, avoided forest conversion, avoided coastal wetland conversion, coastal wetland restoration, avoided grassland conversion, grassland restoration, avoided peatland conversion, peatland restoration, reforestation, and climate-smart forestry. These pathways represent many of the actions with the highest global CMP and offer many co-benefits such as biodiversity conservation and sustainable livelihoods (1, 12, 14, 31). This paper asks: What are the most common constraints to NCS implementation globally, and how do implementation constraints differ across geographies and NCS pathways? Our systematic literature review focuses on the most recent years of publication at the time of the review (2020–2021) to provide a snapshot of current constraints. We map the spatial distribution of implementation constraints by NCS pathway, SDG region, UN subregion, and country. We find that each geography and pathway face multiple, often interrelated constraints that require integrated, comprehensive approaches to overcome. By identifying where and how constraints are limiting the successful implementation of NCS, our results can inform equitable solutions for reducing the implementation gap and more realistic estimates of NCS CMP. To support the development of such solutions, we discuss how adaptive management may be used to systematically diagnose co-occurring constraints in each phase of NCS implementation and develop context-specific integrated solutions that address interrelated constraints.

## Results

**Fig. 1.**
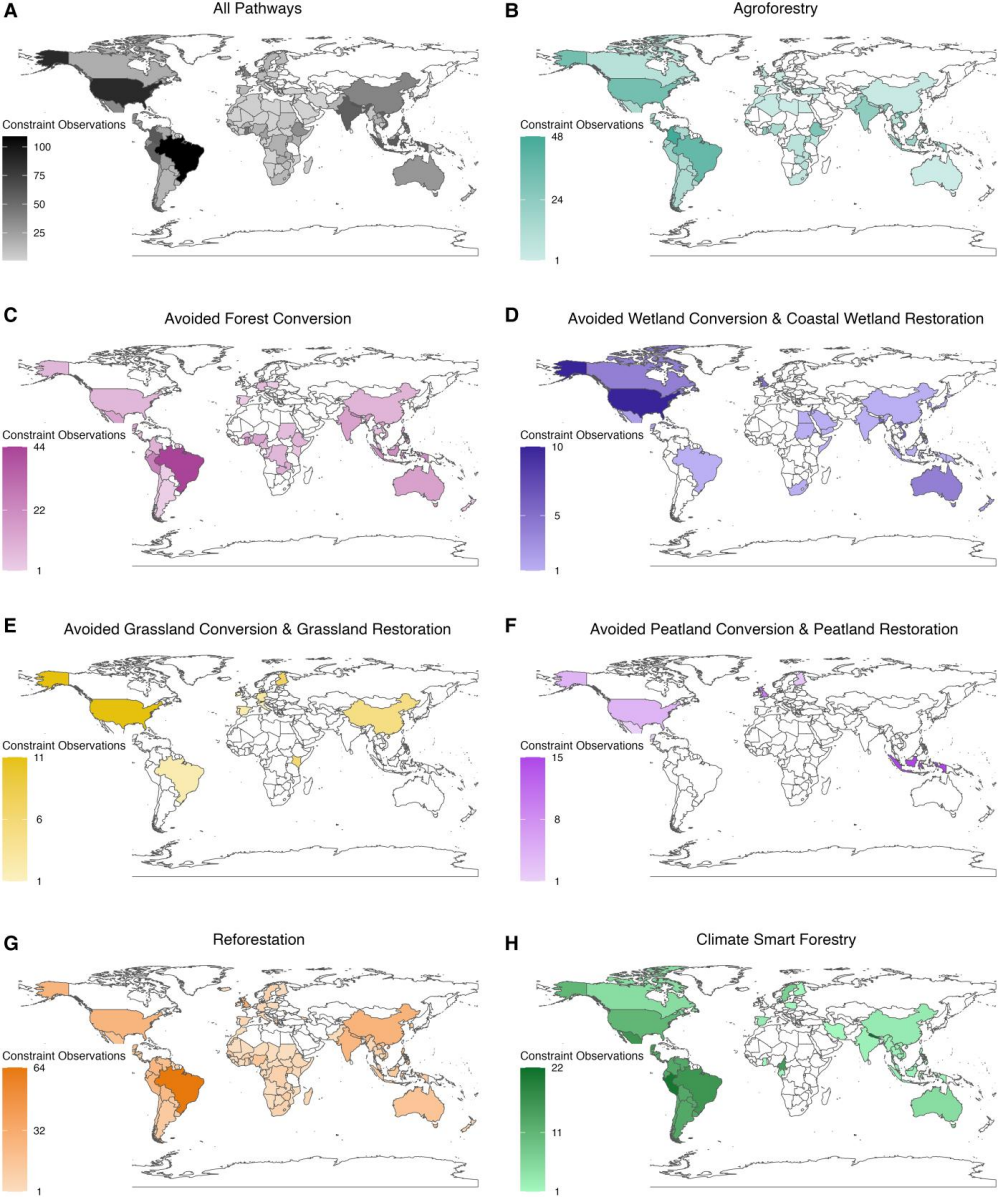
Pathway constraint evidence map. Number of constraints observed in each country A) across all 10 NCS pathways and for the following pathways: B) agroforestry, C) avoided forest conversion, D) avoided wetland conversion and coastal wetland restoration, E) avoided grassland conversion and grassland restoration, F) avoided peatland conversion and peatland restoration, G) reforestation, and H) climate-smart forestry.

Our systematic literature review revealed 2,480 instances of nonbiophysical constraints to NCS implementation feasibility from 352 papers covering 135 countries. We defined “constraints” as factors that prevent NCS adoption, implementation, performance, evaluation, or permanence. We found that the peer-reviewed literature that we reviewed on NCS implementation constraints is geographically unevenly distributed (Fig. 1A). Brazil and the United States, the two countries with the highest biophysical NCS CMP globally (32), had the most reported instances of constraints (167 and 109, respectively). Although several countries with high CMP had strong NCS constraint evidence bases, others did not; for instance, we found no papers reporting constraints in Russia, the country with the sixth-highest CMP (32). Latin America and the Caribbean was by far the SDG region with the most instances of reported constraints (42.1% of constraint observations), followed by Sub-Saharan Africa (16.5%).

We found that there is also uneven coverage of NCS pathways and ecosystems in literature on NCS constraints (Fig. 1). Reforestation was the pathway with the most papers reporting constraints (821 instances of constraints across 140 papers), followed by agroforestry (736 instances of constraints across 128 papers), and avoided forest conversion (401 instances of constraints across 108 papers). Forest pathway (avoided forest conversion, reforestation, and climate-smart forestry) constraints comprised 63.1% of all instances of reported constraints. Wetlands (4.0%), grasslands (1.9%), and peatlands (1.5%) had the fewest observed instances of constraints of all ecosystems. The only agriculture pathway included in our analysis, agroforestry, comprised an additional 29.7% of instances of reported constraints. Across all ecosystem types, approximately twice as many instances of restoration constraints (*n* = 955) were observed as avoided ecosystem conversion constraints (*n* = 527).

In the remainder of this paper, we use abbreviated names for the constraints. In the Supplementary material, we include the full name for each of the 39 constraints, its description, and the constraint category to which it belongs (Table S1 and Dataset S1) (33).

## Most common constraints

**Fig. 2.**
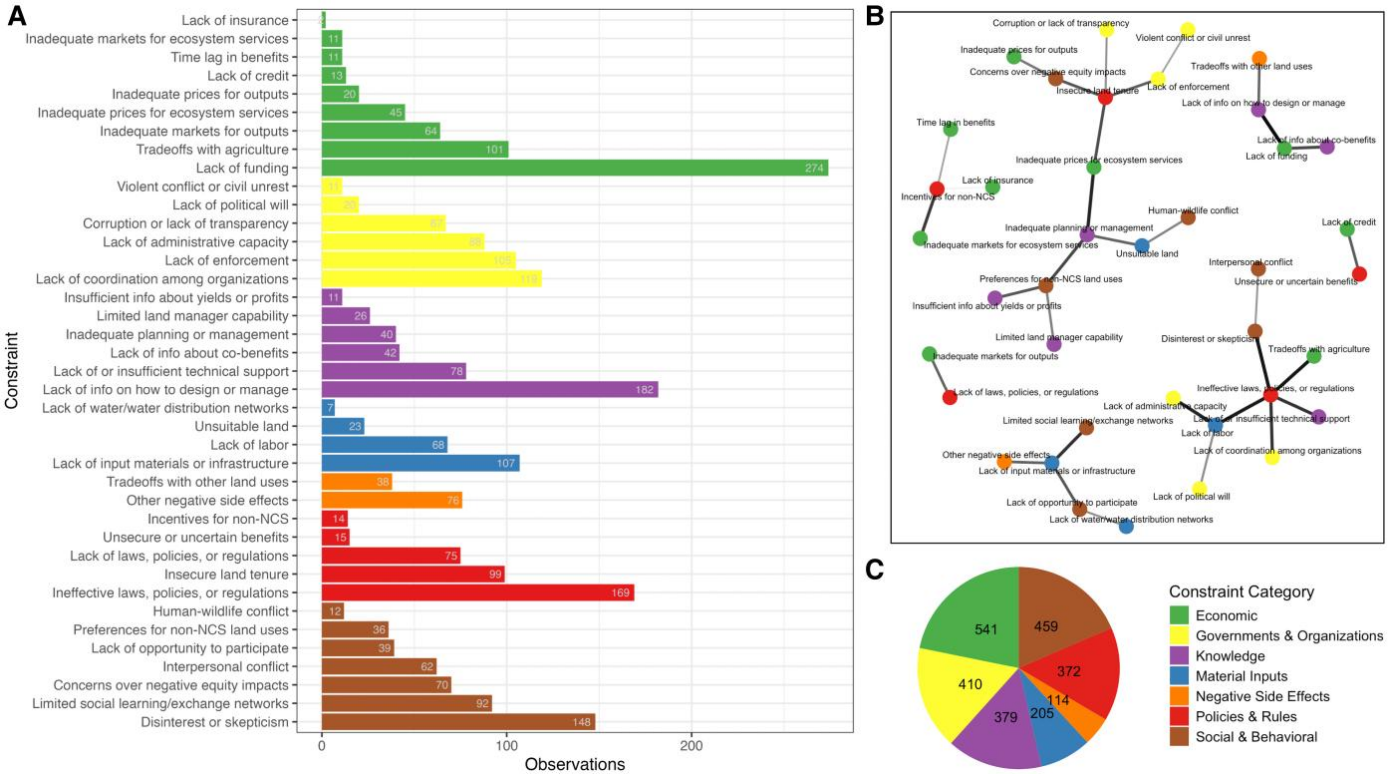
Constraint frequency and co-occurrence. A) Number of observations of each constraint, organized by constraint category. B) Co-occurrence frequency—network graph illustrating constraints that most commonly co-occurred within a UN subregion, based on the highest Jaccard similarity. Nodes represent individual constraints, color-coded by category. Connectors indicate the strength of co-occurrence measured using the Jaccard similarity index, with thicker lines indicating stronger co-occurrence. The visualization represents pairwise relationships between constraints, not clusters. C) Number of constraint observations in each constraint category.

“Lack of funding” was the most frequently and widely observed constraint (274 observations from 65 countries) (Figs. 2A and S3–S7). This constraint included cases where NCS implementation was limited by the lack of access to funding or prohibitively high costs of establishing, implementing, or maintaining the NCS pathway. The next most common constraints were “Lack of information on how to design or manage” (182 observations); “Ineffective laws, policies, or regulations” (169 observations); and “Disinterest or skepticism” (148 observations). Economic, Social & Behavioral, and Governments & Organizations were the three most common constraint categories, accounting for 21.8, 18.5, and 16.5% of the total constraint count, respectively (Fig. 2C). No single constraint occurred in all countries in our dataset; even the most widely observed constraint, “Lack of funding,” occurred in just 47% of countries (Fig. S3).

In addition to the presence of individual constraints, we evaluated the co-occurrence of constraints to determine whether certain combinations were commonly identified in the same UN subregion. Some pairs of constraints never occurred together, while others overlapped in 89.5% of the UN subregions in which they were present. Figure 2B shows the constraint that most often co-occurred in the same UN subregion with each constraint. For any node, the constraint it is connected to is the constraint with which it is most frequently identified in the same subregion. The three constraint pairs with the highest co-occurrence in the same UN subregions were (i) “Lack of funding” with “Lack of information on how to design or manage NCS,” (ii) “Disinterest or skepticism” with “Ineffective laws, policies, or regulations,” and (iii) “Tradeoffs with agriculture” with “Ineffective laws, policies, or regulations” (Fig. 2B). Interestingly, the highest co-occurrence tended to be found between constraints from different categories (e.g. an Economic constraint co-occurring with a Knowledge constraint). There were two exceptions where both constraints in a high co-occurrence pair were from the same category: “Violent conflict or civil unrest” with “Lack of enforcement,” and “Interpersonal conflict” with “Disinterest or skepticism of NCS.”

## Constraints across pathways

**Fig. 3.**
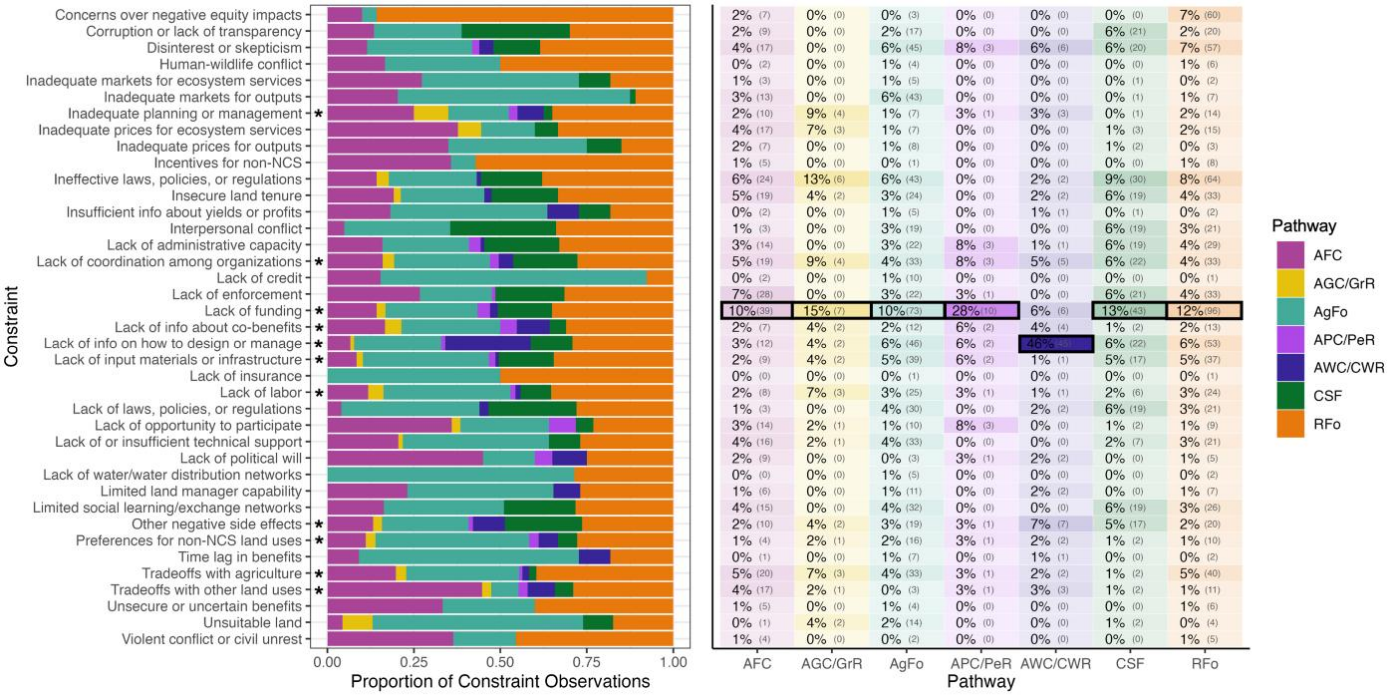
Constraints by NCS pathway. Pathways are avoided forest conversion (AFC), avoided grassland conversion and grassland restoration (AGC/GrR), agroforestry (AgFo), avoided peatland conversion and peatland restoration (APC/PeR), avoided wetland conversion and coastal wetland restoration (AWC/CWR), climate-smart forestry, and reforestation (RFo). Left: Share of NCS pathways in the frequency count of each constraint. For example, RFo accounted for 85.7% of instances of “Concerns over negative equity impacts.” Right: Frequency distributions of constraints by NCS pathway. Shading corresponds to the share of each constraint in the total constraint frequency count of each pathway. Black boxes indicate the most frequently reported constraint for each pathway. For example, “Lack of funding” was observed 39 times for AFC, representing 10% of the constraint share for AFC, the most frequent constraint for this pathway. Asterisks indicate constraints observed in all seven pathways.

All NCS pathways faced at least 16 constraints across at least six of the seven constraint categories (Fig. 3). Eleven constraints were shared by all NCS pathways, suggesting that while some constraints are pathway-specific, others impact NCS implementation more generally. All 39 constraints were identified for the reforestation and agroforestry pathways. Reforestation, avoided forest conversion, and agroforestry had the most similar constraint presence on average within UN subregions (higher Jaccard similarity; see full methods in Supplementary material) and similar constraint shares averaged within subregions (higher Euclidean distance), as did avoided wetland conversion and coastal wetland restoration (Figs. S9 and S10).

Several pathways shared their most frequent constraints. “Lack of funding” was the most frequent constraint for every pathway except avoided wetland conversion and coastal wetland restoration. “Lack of information on how to design or manage” was by far the most observed constraint for avoided wetland conversion and coastal wetland restoration. Reforestation, climate-smart forestry, and avoided grassland conversion shared “Ineffective laws, policies, or regulations” as their second most common constraint after “Lack of funding.” Most (85.7%) observations of “Concerns over negative equity impacts” came from reforestation studies. For avoided forest conversion, “Lack of enforcement” was the second-most observed constraint. Agroforestry differed from other pathways in having “Lack of information on how to design or manage” and “Disinterest or skepticism” as its second- and third-most observed constraints.

## Constraints across geographies

Countries tended to have more similar constraints to other countries within the same UN subregion than to countries from other subregions (Fig. S8B and D). The same was true for countries within the same SDG region (Fig. S8A and C), although the differences were smaller. PERMANOVA analysis confirmed that both the differences in country constraint presence (Jaccard similarity) and constraint shares (Euclidean distance) between SDG regions and UN subregions were statistically significant (*P* < 0.001 for all models). This finding suggests that there are significant differences among SDG regions and among UN subregions in both the constraints they faced and the relative importance of each constraint.

The average Jaccard similarity for SDG regions and UN subregions was 60.8 and 34.9%, respectively. This indicates that pairs of SDG regions shared, on average, nearly two-thirds of their observed constraints, and that pairs of UN subregions shared, on average, one-third of their constraints.

### Constraints across SDG regions

**Fig. 4.**
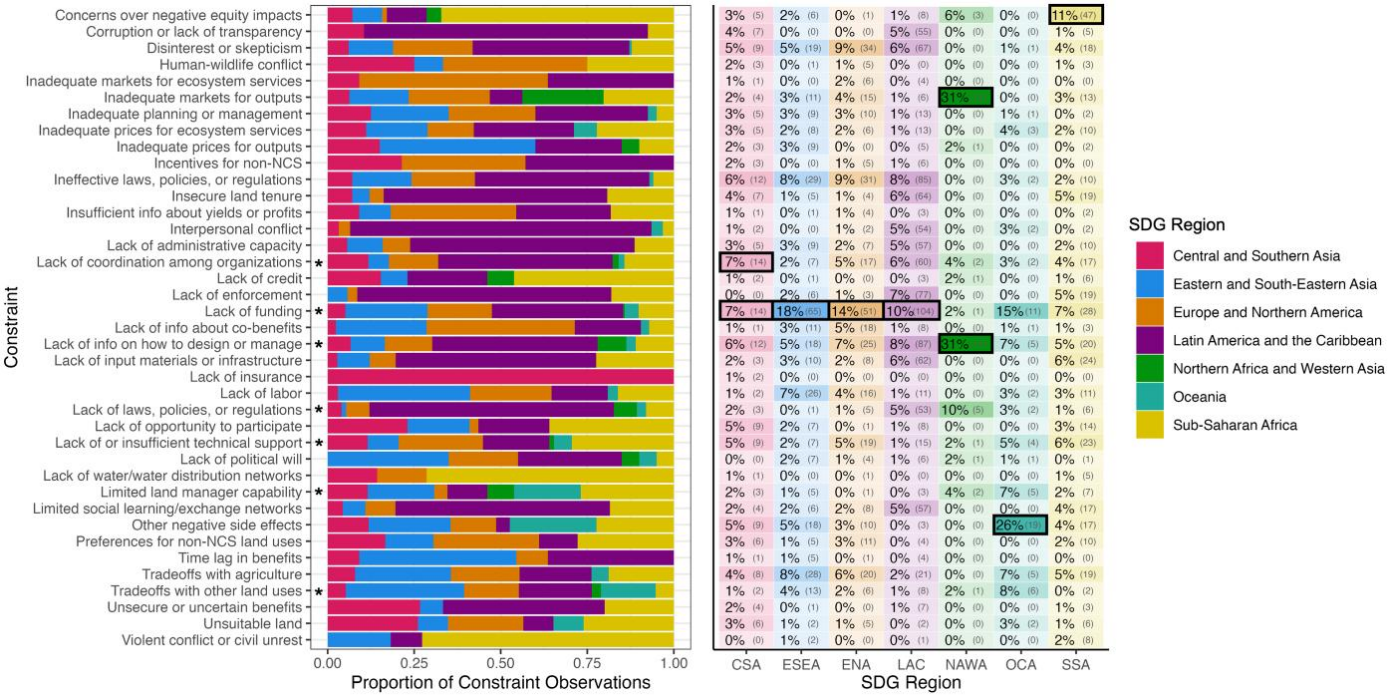
Constraints by SDG region. SDG regions are Central and Southern Asia (CSA), Eastern and South-Eastern Asia, Europe and Northern America, Latin America and Caribbean, Northern Africa and Western Asia (NAWA), Oceania (OCA), and Sub-Saharan Africa (SSA). Left: Percent breakdown of constraint observations by SDG region. For example, 61.7% of instances of “Concerns over negative equity impacts” were found in SSA. Right: Percent share and frequency of each constraint within each SDG region. Shading indicates the share of each constraint in total constraint count in a region. Black boxes indicate the most frequent constraint in each SDG region (two constraints were tied for CSA and NAWA). For example, “Negative equity impacts” was observed 47 times in SSA, representing 11% of total constraint observations in SSA, the most frequent constraint reported for this SDG region. Asterisks indicate constraints observed in all seven SDG regions.

We identified at least 12 constraints in each SDG region (Fig. 4). These constraints spanned all seven constraint categories for all regions except Northern Africa and Western Asia. All SDG regions shared a core set of seven constraints (asterisks in Fig. 4). Regions varied widely in their most common constraints. Both Europe and Northern America and Latin America and the Caribbean had the most observations of “Lack of funding” but differed in their second most common constraints: “Disinterest or skepticism” in Europe and Northern America and “Lack of information on how to design or manage” in Latin America and the Caribbean. The vast majority of observations of “Interpersonal conflict” (87.1%), “Corruption or lack of transparency” (82.1%), “Lack of enforcement” (73.3%), and “Lack of laws, policies, and regulations” (70.7%) were found in Latin America and the Caribbean. Most observations of “Inadequate markets for ecosystem services” (54.5%) were found in Europe and Northern America. The most observed constraints in Eastern and South-Eastern Asia was “Lack of funding,” while Central and South Eastern Asia tied between “Lack of funding” and “Lack of coordination among organizations.” Northern Africa and Western Asia were tied between “Inadequate markets for outputs” and “Lack of information on how to design or manage” as the most common constraints. Top constraints in Oceania were “Other negative side effects,” “Lack of funding,” and “Tradeoffs with other land uses.” In Sub-Saharan Africa, the most common constraints were “Concerns over negative equity impacts” and “Lack of funding.” Most observations of “Violent conflict or civil unrest” (72.7%), “Lack of water/water distribution networks” (71.4%), and “Concerns over negative equity impacts” (67.1%) were observed in Sub-Saharan Africa.

### Constraints across UN subregions

**Fig. 5.**
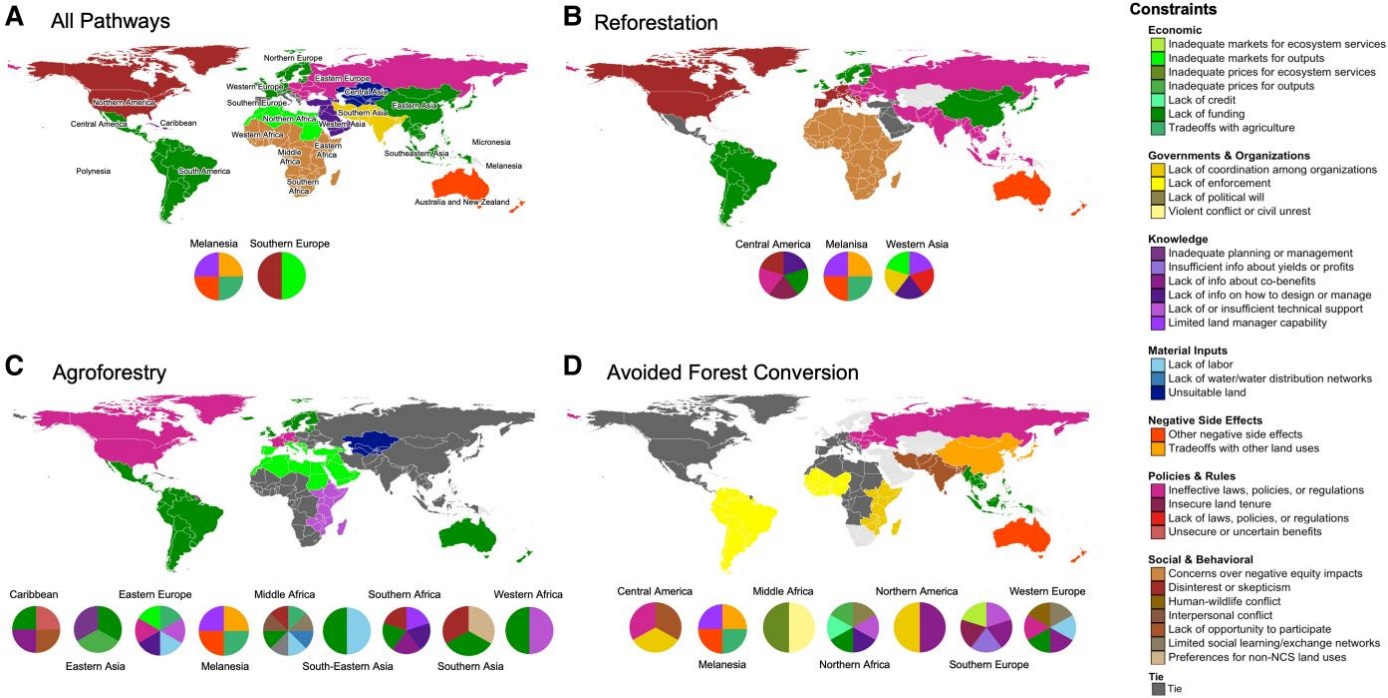
Most frequently identified constraint(s) in each UN subregion for all pathways A), and separately for the three pathways with the most observations in our dataset (together accounting for 79.0% of all observations): B) reforestation, C) agroforestry, and D) avoided forest conversion. Countries are colored on the map based on the most frequently identified constraint in their subregion. Subregions where multiple constraints were tied for the most frequently identified are shown in dark gray on the map and have pie charts below showing the tied top constraints. Subregions where no data were available for that pathway are shown in light gray.

UN subregions offer a finer geographic scale than SDG regions that may be more appropriate for certain supranational policy interventions. We observed an average of 20 constraints per subregion, ranging from no observations in some subregions to almost all in others (36, or 92.3%). We found constraints from all 7 categories in most subregions (13 of 20). The most frequently reported constraints varied considerably among UN subregions, with 12 different top constraints among the 20 UN subregions (Fig. 5A). Interestingly, some subregions that are often aggregated as SDG regions or continents had different top constraints (e.g. different parts of Central and Southern Asia and Europe and Northern America had different top constraints). “Lack of funding” (six subregions) and “Concerns over negative equity impacts” (four subregions) were the top constraints or tied for the top constraint in the most subregions.

Each UN subregion tended to have a different constraint distribution for each pathway, suggesting that the relative importance of challenges varies across pathways. All UN subregions differed in their most frequently identified constraint for reforestation, agroforestry, and avoided forest conversion, the three pathways with the most observations in our dataset (Fig. 5). For instance, South America's top constraint was “Lack of funding” for reforestation and agroforestry, but it was “Lack of enforcement” for avoided forest conversion. Eastern Africa's top constraint for reforestation was “Concerns over negative equity impacts,” but “Lack of or insufficient technical support” for agroforestry and “Lack of coordination among organizations” for avoided forest conversion. Southern Europe's top constraints for reforestation and agroforestry were “Disinterest or skepticism” and “Inadequate markets for outputs,” respectively. In Southern Asia, the top constraints for reforestation and avoided forest conversion were “Ineffective laws, policies, or regulations” and “Lack of opportunity to particulate,” respectively.

### Constraints across countries

**Fig. 6.**
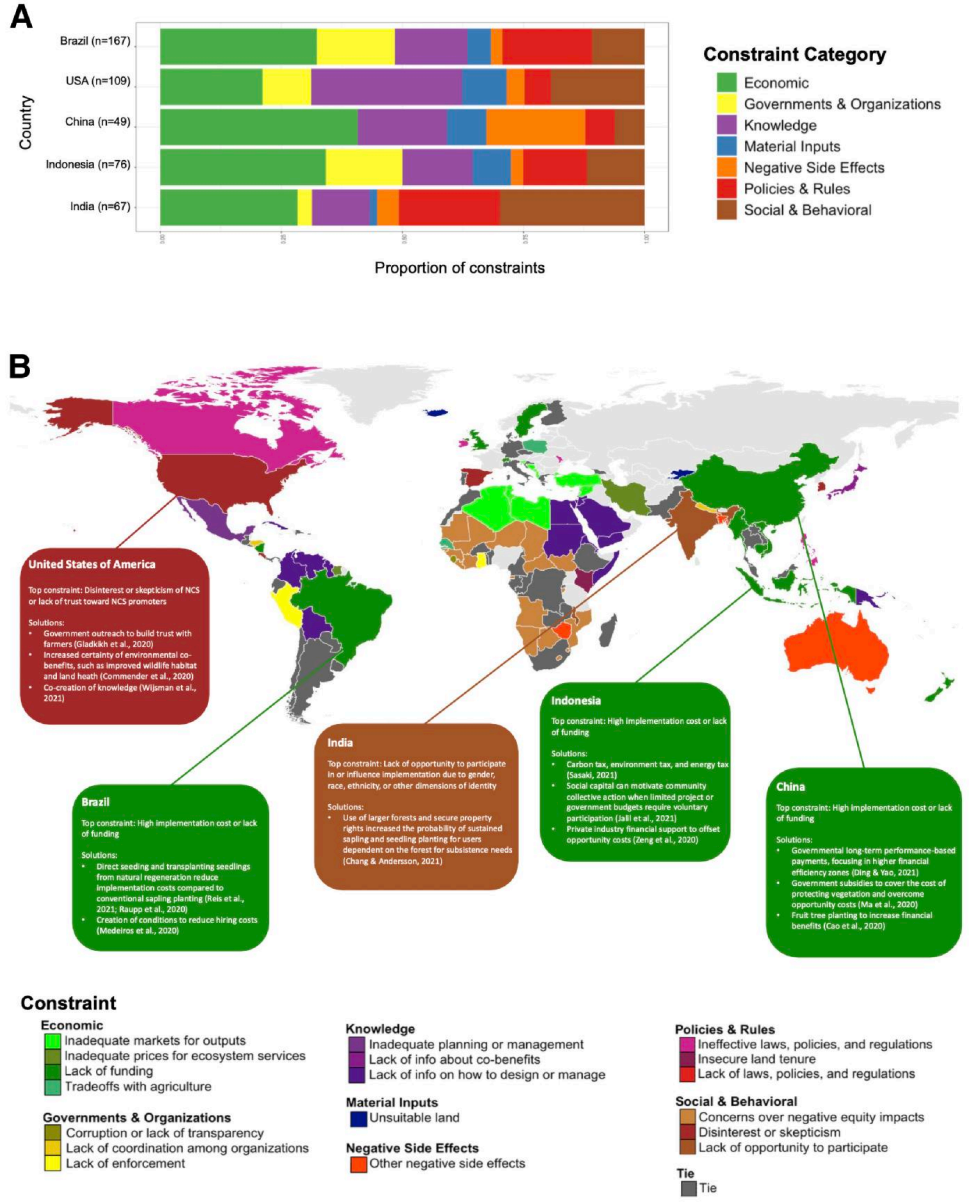
A) Breakdown of constraint categories of constraints observed for the five countries with the highest NCS CMP globally. Parentheses indicate the number of constraint observations for each country. B) The most commonly identified constraint in each country. Dark gray indicates ties. Light gray indicates no data. Boxes show solutions identified in the literature review for the five countries with the highest CMP (23, 34–45).

The most frequently reported constraints varied even more at the country level. Almost all constraints (32 of the 39 constraints) were the top constraint or tied for the top constraint for at least one country (Fig. 6B), highlighting the breadth of constraints globally. “Lack of funding” was the most frequently observed constraint in the highest number of countries (30 countries), followed by “Lack of information on how to design or manage” (27) and “Concerns over negative equity impacts” (27). Constraints also differed across World Bank income groups (Fig. S2).

We found that countries with the highest NCS CMP—Brazil, the United States, China, Indonesia, and India (32)—had high constraint diversity, including constraints from almost every category (Fig. 6A). Most of the 39 constraints were observed in Brazil (31 constraints), the country with the largest evidence base (*n* = 167). Interestingly, India had the second highest constraint richness (27 constraints) of these 5 high CMP countries, despite having the second-smallest evidence base (*n* = 67). Twenty-four unique constraints were observed in the United States, 18 in Indonesia, and 15 in China.

## Solutions

In addition to identifying constraints, some studies also identified, recommended, or evaluated solutions to advance NCS implementation (example solutions to each constraint in Table S3, full data available in available in Dataset S1). Of the 2,480 constraint observations in our sample, 58.0% included information about solutions, with 9.3% of these also including details about the cost of implementing the proposed solution. The Policies & Rules category had the most constraints with proposed solutions (73.6%) but had the lowest percentage of solutions with cost information (1.1%). The Economic constraint category had the highest proportion of solutions with cost information (27.6%), of which “Inadequate prices for ecosystem services” was the constraint with the highest proportion of solutions with cost information (44.4%). “Insufficient information about yields or profits” (100%), “Concerns over negative equity impacts” (92.9%), and “Interpersonal conflict” (90.3%) were the constraints with the highest proportions of recommended solutions, although cost information was rarely provided for the solutions. Northern Africa and Western Asia was the SDG region with the highest proportion of constraints with solutions (97.9%), although none of these papers included solution costs. Papers offered diverse solutions to the same constraints. For instance, “Lack of funding” was the most common constraint in Brazil, Indonesia, and China, three of the countries with highest CMP, although solutions and implementing entities identified in the literature review varied across these countries (Fig. 6B).

## Discussion

In this paper, we advance the growing body of research on spatial prioritization of NCS by conducting a spatially explicit analysis of recent nonbiophysical constraints to NCS implementation efficacy. We found that “Lack of funding” was the most frequently reported and widespread constraint globally, with Economic emerging as the most frequent constraint category. However, Economic constraints comprised less than a quarter of all constraint observations, and each geography and NCS pathway faced many co-occurring constraints spanning multiple categories. By identifying constraints for each country, UN subregion, SDG region, and NCS pathway, our study provides a reference for practitioners, policymakers, and researchers to assess the most prevalent constraints in their area of focus and helps enable more realistic estimates of NCS CMP. Our findings underscore the need for context-specific, integrated solutions that address multiple interrelated constraints simultaneously, likely requiring trans-sectoral and trans-disciplinary collaboration with attention to equity. To this end, we explore how adaptive management can support NCS implementation by systematically diagnosing and addressing co-occurring constraints across implementation phases.

## High implementation costs and lack of funding was the most-observed constraint

“High implementation costs or lack of funding” was the most frequently and widely observed constraint. It was the top constraint for almost every pathway and among the top three constraints in almost every SDG region. Economic was also the most common constraint category. This is not surprising given that 393 billion USD a year would be needed to mitigate 6.0 GtCO_2_ per year through forest NCS alone (46). This finding aligns with Karki et al. (19), who found that financing was one of the main limiting factors to land-based mitigation technology implementation at scale; and Schulte et al. (22), who found that performance-based finance was among the most mentioned enabling factors for NCS.

There are a variety of potential reasons why “High implementation costs or lack of funding” was so frequently cited as a constraint. Like most changes in land management, NCS require investments prior to, during, and after initial implementation. For example, a study found that shade cacao agroforestry farmers in Ghana experienced high investment costs in establishing farms, as well as high operating and maintenance costs throughout the production cycle (47). Consequently, these farmers may not recover their costs nor make profits. In other circumstances, where NCS practices may be economically profitable over multi-year periods, initial funding needs may exceed individual land manager funding capacity. Available third-party funding for NCS often is insufficient, not well coordinated, or difficult to access (48, 49).

To address these limitations, it may be necessary to mobilize public and private funding mechanisms that are matched to appropriate NCS implementation phases and to improve coordination between funders and implementers (Table S3). For instance, Calle (50) recommended that hybrid financial incentive schemes that offer both in-kind support for immediate action and smaller financial rewards for good stewardship could aid silvopastoralists in Colombia to afford high input and maintenance costs while achieving sustainable conservation outcomes. Sasaki (34) suggested creating a carbon, environmental, or energy tax in Southeast Asia like those implemented in Europe to mobilize additional financing for NCS. However, given the prevalence of other constraints, our findings indicate that funding alone will be insufficient to realize the full biophysical potential of NCS (34), and we recommend identifying solutions that consider co-occurring constraints.

## Global variation in constraints

The distribution and composition of constraints vary across SDG regions, UN subregions, countries, and income groups (Figs. 4–6, S2, S8, and S11). Different UN subregions only shared a third of their observed constraints on average, and even neighboring countries differed in observed constraints. Even within a single NCS pathway, constraints differed by region. This variation could be attributable to the diverse economic, institutional, geophysical, technological, sociocultural, and environmental contexts these geographies represent (51). These broader contexts lead to unique challenges and priorities that influence NCS implementation in different locations. Factors such as a country's government effectiveness, regulatory quality, political stability, history, personal rights, nutrition and basic medical care, and access to information and communications may all affect the feasibility of NCS implementation (51). For instance, “Disinterest or skepticism,” which includes lack of trust of NCS promoters, was the most observed constraint for reforestation in Northern America and Southern and Western Europe (Fig. 5B), likely reflecting discriminatory land ownership and land management histories leading to distrust (52), strong value of agency amongst farmers (53), and centuries-old agrarian cultural heritage (54) in those geographies.

This result has several implications for research and management. Understanding the challenges unique to each geography can ensure that NCS implementation supports rather than conflicts with the diverse social–ecological goals prioritized by communities. Integrating equity and cultural competency into NCS planning and implementation will be critical to addressing the unique constraints identified across regions. Additionally, understanding constraints can improve the accuracy of NCS feasibility assessments and realistic NCS CMP estimates and reduce uncertainty (5). The location-specific nature of constraints necessitates context-specific, tailored solutions that address the underlying causes and local dynamics of each constraint. It is likely that constraints further vary and manifest differently at the subnational level, which should be evaluated in future research.

## Geographic grouping of constraints within UN subregions

We observed two notable ways that constraints cluster geographically. First, countries tend to have more similar constraints with other countries in their SDG region and UN subregion than with those in other regions or subregions. Proximate countries may share constraints due to similarity in sociocultural, political, and economic conditions, such as similar governance structures, regional agreements, and cultural attitudes toward land use and conservation (55–58). Biophysical continuity, including shared climate conditions, transboundary ecosystems, and common land-use pressures, may also contribute to the clustering of constraints (58, 59). Due to the commonalities of constraints among proximate countries, there exists potential for the development of collaborative supranational policy and other initiatives aimed at addressing constraints to avoid duplicative efforts and take advantage of potential economies of scale compared with isolated domestic efforts.

Second, the similarity of constraints was higher for countries within the same UN subregion than within the same SDG region, which is intuitive given the smaller size of UN subregions. This pattern likely arises because countries in the same UN subregion often share more fine-scale socio-political, economic, and environmental characteristics than those in the broader SDG region. For instance, countries within the same UN subregion may have more interconnected trade and financial systems, more similar legal and governance structures, and shared policy histories—such as regional environmental agreements or colonial legacies—that shape how NCS implementation is constrained (55–58). Additionally, UN subregions tend to encompass ecosystems and climate zones that are more homogeneous than those spanning an entire SDG region, which may lead to more similar biophysical challenges like restoration feasibility, land tenure conflicts, and vulnerability to climate extremes (58, 59). Therefore, policy interventions for NCS may be more effective at the UN subregion level, where countries face more comparable constraints. Supranational interventions could be particularly beneficial for resource- and data-poor countries within the same UN subregion.

## Co-occurrence of constraints from different categories necessitates integrated solution development

Multiple constraints often occurred together, indicating the importance of addressing constraints through integrated interventions. In our dataset, the average country faces 7 constraints, and the average UN subregion faces 20 constraints across almost all categories. Consequently, there is neither single constraint nor a single category in each geography the removal of which would unlock the full biophysical CMP. The large number and diversity of constraints reported, and the time it may take to mitigate them, suggests that it is unlikely that the full biophysical CMP of NCS can be realized, at least in the short to medium term.

These multiple co-occurring constraints for each geography represent diverse categories, underscoring that integrated solution development will likely cross sectors and disciplines. Almost every SDG region faced constraints from all seven categories (Fig. 4), as did four of the five countries with the highest CMP (Fig. 6A). Constraints with the highest co-occurrence were almost always from different categories (Fig. 2B). For instance, “Lack of funding” (Economic category) and “Lack of information on how to design or manage NCS” (Knowledge category) were the constraints pair that co-occurred in the same UN subregion most frequently (Fig. 2B). These two constraints may exacerbate each other due to local economic instability limiting the ability to dedicate resources to research and information dissemination, or regions with little knowledge on how to design or manage NCS projects being unable to compete for NCS funding. In some cases, co-occurrence of constraints from difference categories may indicate systemic issues that transcend individual constraint categories. For instance, the constraint pair with the second highest co-occurrence, “Disinterest or skepticism of NCS” (Social and Behavioral) and “Ineffective laws, policies, or regulations” (Policies and Rules), may reflect systematic or historical challenges in governance and lack of trust in institutions. This indicates that solutions within a given geography cannot focus on just one dimension (e.g. Economic or Knowledge), and could benefit from intersectoral collaboration that pools resources and expertise across diverse fields to develop holistic solutions.

There are at least five reasons why integrated policy interventions and solutions that account for local contexts and multiple co-occurring constraints may be more effective and efficient than isolated approaches. First, constraints often have causal interdependencies, where one constraint underlies another or multiple constraints reinforce each other, creating vicious cycles (60). Addressing a constraint may directly or indirectly alleviate another co-occurring constraint in the same geography, facilitating synergies. For instance, Reyes et al. (61) discuss that policy uncertainty drives other constraints to NCS including increased risk aversion and decreased investment in tree plantations in Chile. This is an example of how addressing one NCS constraint (in this case “Ineffective laws, policies, or regulations”) may alleviate others (in this case “Disinterest or skepticism”), the constraint pair with the second highest co-occurrence rate (Fig. 2B). Second, many constraints stem from underlying historical or systemic factors—such as inequality or food insecurity—meaning that addressing these root causes is likely to lead to more sustainable, long-term solutions. For instance, a community-based forest restoration project in the Philippines faced a variety of barriers (62), many of which stemmed from substantial food and financial insecurity issues affecting the community. Addressing these barriers by increasing sustainable livelihood opportunities and food security, especially for women, may improve long-term restoration success (24, 62).

Third, solutions that only resolve one or some of the constraints in a geography may indirectly cause or exacerbate other constraints, thus failing to improve NCS feasibility. For instance, we found that many UN subregions face both “Lack of enforcement of laws, policies and regulations” and “Insecure land tenure” (Fig. 2B), but in some cases, addressing the former exacerbates the latter, as observed in community-managed forests in the Southern Sierra of Oaxaca, Mexico (63). Fourth, constraints are often intertwined, meaning that addressing one may require tailoring approaches or engaging different stakeholders depending on the presence of other constraints in the same geography. For instance, strategic spatial planning should be tailored to the multiple desired objectives (“Inadequate planning and management”), and these planning processes may be shaped based on stakeholder trust (“Disinterest or skepticism”) and knowledge (“Lack of or insufficient technical support”), as exemplified in the Philippines (64). Fifth, because the majority of NCS management pathways co-occur, alleviating a constraint identified for a specific pathway may also benefit other pathways in the same geography (65). These reasons underscore the importance of identifying co-occurring constraints and developing context-specific solutions that engage diverse stakeholders, mitigate unintended consequences, and ensure long-term sustainability.

## Equity concerns especially prevalent in reforestation

Equity is a foundational principle of NCS, yet historical and ongoing injustices in natural resource management underscore the need for a social equity lens in implementation (2). Inequity can reduce the uptake, efficacy, or longevity of NCS (66–68). “Concerns over negative equity impacts” was the second most widely observed constraint across countries (61 countries; Fig. S3). It was disproportionately associated with reforestation; this pathway accounted for nearly 90% of all recorded observations for this constraint (Fig. 3). Our review identifies several factors contributing to equity concerns surrounding reforestation. First, there are concerns over negative equity impacts for Indigenous people and local communities if reforestation is implemented using top-down approaches that disregard local people's needs and preferences. When reforestation occurs on land that was cleared and converted to agriculture, it can negatively impact the livelihoods and food security of local communities (66, 69, 70). This approach can exacerbate existing inequalities by benefiting government, corporate, or international interests at the expense of local communities, who may be excluded from accessing land, natural resources, or funding (28, 66, 70, 71). Second, an emphasis on reforestation for climate mitigation can shift the burden of mitigating climate change to lower income nations, reinforcing historical patterns of land appropriation (69). Most observations of “Concerns over negative equity impacts” were from low and lower-middle income countries (Fig. S2). Equity concerns were pronounced in Sub-Saharan Africa (Figs. 4–6), potentially driven by pervasive socioeconomic inequalities, historical land dispossession, marginalization of certain communities, misclassification of grassy biomes as degraded areas suitable for reforestation, and dependence on rangelands for food security and livelihoods (69).

To address the equity challenges associated with reforestation, our literature review identified several integrated approaches (Table S3). Livelihood opportunities for forest-dependent Indigenous peoples and local communities can be supported through forest-based enterprises, such as bamboo furniture and essential oil production in Nepal's Tarai region (27, 66). Integrating reforestation with agriculture may offer more equitable benefits to local communities (70). Additionally, research points to the importance of enabling local participation in the design, planning, implementation, and monitoring of restoration interventions (66), as well as other NCS (27). Restoration priority maps should be improved to accurately reflect areas in need of reforestation—avoiding afforestation of grassy biomes—and ensuring that reforestation does not adversely impact the livelihoods and food security of local communities, particularly in developing nations (69). Improving land tenure and creating local payments for ecosystem services (PES) systems targeted at engaging poorer and smaller farmers may facilitate more equitable access to resources (67, 71).

Many other constraints beyond “Concerns over negative equity impacts” also have equity implications that are critical to address in implementing any pathway. When NCS design and implementation fail to take equity considerations into account, NCS may lead to inequitable outcomes or exacerbate existing underlying inequalities. “Human–wildlife conflict” is an example of a constraint that may arise from NCS implementation and lead to inequitable outcomes in the form of safety and livelihood impacts on communities, while “Insecure land tenure” is an example of a constraint which may be driven by underlying systemic issues and exacerbated by the implementation of NCS. In both cases, addressing constraints involves adapting the design and implementation of the NCS to not only mitigate negative impacts but contribute to more equitable outcomes. Inclusive and participatory approaches to designing and implementing NCS can improve the legitimacy and effectiveness of project outcomes (72, 73).

## Addressing ineffective laws and policies

“Ineffective laws, policies, or regulations” was 1.5 times more common than “Lack of enforcement” and twice as common as “Lack of laws and policies,” suggesting that while laws and policies often exist, they need to be made more effective. Complex, ambiguous, contradictory, and outdated laws and policies can be difficult to interpret and implement, leading to loopholes, exclusion of those without technical and legal knowledge, or uncertainty among potential adopters about the legality of or support for specific NCS pathways or implementation designs (74–76). For example, some forest renewal policies in western Canada are based on historic European forestry principles, emphasizing replication of the original stand type and projecting growth based on oversimplified models (76). Some policies may pose unnecessarily burdensome conditions on adopters, such as through contradictory or demanding requirements. For instance, in Puerto Rico, state and federal incentives support conflicting coffee farming systems (35). Many coffee agroforestry farmers mistakenly believe that sun farming is a requirement to qualify for state agricultural incentives, which undermines the conservation goals of shade coffee programs (35).

To resolve these issues, Gladkikh et al. (35) recommend that government agencies work together to harmonize conservation and agricultural incentives, so farmers are not forced to choose between them. Moreover, having clear and supportive laws may help enable external funding flows. For example, Waring et al. (77) found that funders' perception of political and regulatory risk drive their NCS investment allocations. This finding suggests that while many countries have established legal frameworks, efforts should focus on enhancing the effectiveness and congruence of existing laws and policies.

## Grasslands, peatlands, and wetlands are understudied

We found that constraints to NCS in peatlands, grasslands, and wetland ecosystems are understudied relative to their global extent and CMP. Fewer constraints were found for grassland (1.9% of total constraints), peatland (1.5%), and wetland (4.0%) NCS than forest (63.0%) NCS. Forests cover 31.7% of global land area, while grasslands cover 40%, peatlands cover 2.8%, and wetlands cover 6% (78–80). Similarly, Chang et al. (65) found that the evidence base on co-benefits for NCS pathways in these ecosystems was disproportionately small compared with their CMP. More research is needed to understand the nature of constraints to these pathways and to develop more realistic estimates of their near-term feasible CMP.

This is especially important because these pathways had fewer similar constraints than the forest-related pathways (Figs. S9 and S10). For example, avoided coastal wetland conversion and coastal wetland restoration constraints heavily emphasized “Lack of information on how to design or manage,” much more than pathways in other ecosystems. This likely reflects the specialized knowledge and technical expertise required for effective wetland management, and potentially identifies the need for more research on wetland management best practices and technical advice (81). “Lack of funding” comprised a higher proportion of avoided peatland conversion and peatland restoration constraints than any other pathway. While peatland restoration is among the NCS with the highest CMP globally (12), peatland restoration is expensive to implement and has high opportunity costs, because it is often less profitable than alternative management scenarios (e.g. timber or bioenergy production) (82). “Inadequate planning or management” comprised a higher proportion of avoided grassland conversion and grassland restoration constraints than other pathways, which may be attributed to the management challenges associated with both preventing soil degradation from overgrazing and shrub encroachment from grazing abandonment (83). Consequently, unique solutions may need to address constraints in these ecosystems, although it may be harder to develop tailored solutions given the dearth of information on constraints to these ecosystems.

## Applying adaptive management to diagnose and address co-occurring constraints

Our study provides evidence that each geography faces a suite of constraints (previous studies on constraints to NCS implementation were not spatially explicitly), often from different categories. We discussed reasons why developing solutions that take these co-occurring constraints into account may be more effective and sustainable. Here we suggest how practitioners can connect the constraints and categories we identified to NCS projects and develop solutions for these constraints. Constraints may arise before, during, or after an NCS project is implemented, and may affect project uptake, complete and effective execution, progress assessment, or permanence. Consequently, practitioners may find adaptive management to be a useful diagnostic framework for identifying constraints at each phase of an NCS project (Fig. S12A). Adaptive management, a widely used approach for addressing environmental challenges under conditions of uncertainty, is particularly relevant to NCS because it emphasizes learning and adjusting over time, aligning well with the often complex nature of NCS implementation (84–86). This approach could be useful to guide practitioners to systematically identify constraints within each phase of an NCS project, allowing for more precision in the identification of temporally co-occurring constraints. We hope this helps enable practitioners to account for key constraints prior to implementation. Identifying constraints at the phase where they first occur can help surface more effective and sustainable solutions, as interventions can prevent downstream impacts, rather than simply mitigating symptoms later in the process. Importantly, this framework can be applied at multiple geographic scales, from subnational to national and supranational, and it may be particularly useful in regions with limited existing research on implementation constraints.

The understanding of which constraints are co-occurring in the same project phase may affect the choice of solutions. To illustrate this, we provide an example for each phase from our literature review dataset. In each case, we highlight an Economic constraint, the most frequently observed constraint category, alongside a co-occurring constraint from a different category (Fig. S12A). The project phase affected by a constraint may impact how a constraint manifests and thus how it is solved. For instance, “Lack of funding” presents challenges in both the Implement and Evaluate phases, but the nature of these challenges and their solutions differ. While more efficient resource allocation can help in both cases, in Sweden, funding shortages were exacerbated by labor shortages in the Implement phase (87), whereas in New Zealand, they were compounded by a lack of standardized co-benefit quantification in the Evaluate phase (88), requiring distinct solutions. Constraints that co-occur within a phase may affect how the constraint both manifests and can be addressed, including which stakeholders can be involved. For instance, increasing PES can alleviate financial barriers, but if structured equitably—accounting for gender and socioeconomic disparities—it can also address the “Lack of opportunity to participate” constraint, empowering stakeholders and improving inclusivity in the Assess phase (89).

To assist practitioners in identifying constraints and solutions relevant to their projects, we provide a set of example guiding questions designed to systematically assess co-occurring constraints at each phase of project implementation (Fig. S12B), inspired by Moser and Ekstrom (90). Since constraints from all seven categories may arise at any phase, these questions are structured to help identify the full range of potential constraints. This approach may be particularly useful for practitioners whose positionality, interests, or background led them to focus on certain types of constraints over others. Additionally, we include example guiding questions for developing solutions, designed to encourage practitioners to consider integrated approaches that span multiple constraint categories and may require trans-sectoral collaboration.

## Future work

Our results and open source database provide a foundation for future research on NCS planning and implementation. By identifying geographic relationships between constraints, our database can help uncover co-occurrence of constraints that may indicate underlying issues affecting NCS implementation, such as equity, communication, or education concerns. Unlike many studies focused on the global biophysical CMP of NCS, our database can inform the assessment and mapping of broader NCS implementation feasibility. This information could be used to help identify which constraints most inhibit NCS deployment in a given geography and develop more realistic estimates of feasible NCS mitigation potential both by country and globally. Such future research could assess the severity of individual constraints; their mutability: whether and to what extent they can be addressed through limited direct interventions like financing and technical support or require broader societal changes such as improvement in governance capacity and quality or ending violent conflict; and evaluate what proportion of a geography's NCS potential they affect.

Future work could enlarge the evidence base on constraints to NCS implementation for geographies and pathways with limited data. In the meantime, policymakers, practitioners, and researchers in countries with limited observations may draw on the data we present, considering specifically constraints from other countries in the same UN subregion or SDG region. Future studies could also focus on finer spatial resolutions, such as country or subnational levels, to capture variations in constraints and solutions at scales relevant for individual NCS projects.

## Limitations

Our study has several limitations. First, research coverage is highly uneven across countries and pathways. There is risk of false negatives, especially for countries and pathways with limited data, so the absence of published evidence of constraints in a country should not be interpreted as evidence that there are few constraints. Second, we only included peer-reviewed scientific literature, excluding databases and gray literature, which could introduce a bias if certain constraints, geographies, and pathways are more likely to be represented in peer-reviewed journals. The geographic diversity of findings may have been further limited by only using English search terms. Constraints identified in papers likely reflect the authors' disciplines and demographics, which could lead to bias or gaps (91).

Some constraints may only apply to specific parts of a country due to variations in ecosystems, socioeconomic conditions, and political factors, meaning our country-level findings may not hold at the subnational level. Also, the frequency of a reported constraint does not necessarily indicate its importance or severity, as some topics may be more socially acceptable or frequently discussed in particular countries. For instance, the finding that “Lack of funding” is the most frequently observed constraint should be interpreted cautiously. Stakeholders might view funding as easier to discuss or address than constraints requiring deeper structural or behavioral change, leading to its more frequent mention in the literature we reviewed. Most observations of “Inadequate markets for ecosystem services” were found in Europe and Northern America, likely due to more established mechanisms for evaluating and trading these services, while other regions may not yet have developed such frameworks.

## Conclusion

Closing the NCS implementation gap requires identifying and addressing nonbiophysical constraints to implementation. Our spatially explicit assessment of NCS constraints provides new insights into the complexity of NCS implementation challenges and how constraints interact within regions. We highlight constraints relevant to each country, UN subregion, and SDG region, revealing their frequency, diversity, and geographic patterns. “Lack of funding” was the most widely and frequently identified constraint, and Economic constraints were the most frequently reported category globally. However, every geography and NCS pathway faced multiple, co-occurring constraints spanning different categories. The co-occurrence of constraints across categories suggests that addressing a single barrier in isolation is unlikely to fully unlock CMP. Rather, effective solutions likely will need to integrate interventions that address constraints in multiple categories and consider underlying systematic factors, reinforcing the need for trans-sectoral collaboration that pools resources and expertise across fields. Additionally, we find that NCS constraints are more similar within UN subregions than across broader SDG regions, suggesting that supranational initiatives at the subregional level may be particularly effective.

To support efforts to overcome these constraints, adaptive management may be a useful diagnostic framework to help practitioners systematically identify constraints and design solutions that account for interdependencies among constraints at each phase of NCS project implementation. Identifying constraints at the phase where they first occur can also surface more effective and sustainable solutions, as early interventions can prevent downstream impacts rather than simply mitigating symptoms later. Because NCS implementation is shaped by overlapping and interacting barriers, solutions that are context-specific and coordinated may improve effectiveness, efficiency, equity, and long-term sustainability. Unlocking CMP and maximizing the co-benefits of NCS may require collaborative initiatives that integrate multiple dimensions, such as innovative work at the nexus of policy, finance, governance, and science. By identifying key barriers at each implementation phase and providing a spatially explicit dataset on constraint distribution, our study supports future work on more realistic CMP estimates, targeted interventions, and evidence-based funding prioritization to improve the feasibility and impact of NCS worldwide.

## Methods

We conducted a systematic literature review on constraints to NCS. A detailed description of the methods is available in the Supplementary material.

We conducted literature searches for 11 NCS pathways using the Scopus and Web of Science databases on 2021 November 4 (see Supplementary material for search terms). The pathways included agroforestry, avoided forest conversion, avoided coastal wetland conversion, coastal wetland restoration, avoided grassland conversion, grassland restoration, avoided peatland conversion, peatland restoration, reforestation, climate-smart forestry, and regenerative agriculture. Searches included synonyms for pathways and constraints, intentionally broadening the scope to include studies that may not explicitly use an NCS or nature-based solutions (NbS) framing. This search yielded 26,432 publications (Fig. S1). Papers solely focused on regenerative agriculture were excluded for reasons of scope.

The remaining studies (*n* = 20,045) were prescreened based on titles and abstracts, applying two inclusion criteria: explicit mention of one of the 10 studied NCS pathways and an indication of constraint(s) to NCS implementation. Abstract screening was conducted manually and using the machine learning tool Abstrackr (92), with two rounds of intercoder reliability tests (Table S4). From this, 4,299 papers (publication years 1961–2021) were identified for full screening. Given that constraints may change over time, we focused full screening on the most recent papers, published in 2020–2021 (*n* = 1,821), representing 42.3% of the identified studies. Of these, 352 papers were fully coded for constraint information and the remaining were rejected, because they lacked information about constraints. These papers were coded for 43 variables (detailed in Supplementary material), including NCS type, implementers, geography, constraint, affected stakeholders, and solutions.

We classified the unique constraint observations from our systematic literature review into 39 constraints (Table S1). We developed these constraints iteratively through emergent coding, and the wording of each was chosen such that it encompasses the range of specific permutations of each constraint found in the reviewed papers (Table S2). Each observed constraint was reviewed by at least three authors for consistency.

We then grouped these constraints into seven categories (Table S2). Economic constraints arise from a lack of access to financial resources or services (credit or insurance), or to markets (due to the absence of markets or high transaction costs), or a lack of rentability of NCS (due to high costs or low prices for NCS outputs or ecosystem services). Economic constraints also include tradeoffs with agriculture and time lags in benefits. Knowledge constraints are caused by a lack of information (about the design, management, biophysical or economic performance, climate mitigation and co-benefits of NCS; either in the scientific community or among land managers), of technical advice, or of land managers' ability to effectively implement the NCS (due to land manager limitations in terms of literacy, numeracy, or technological capacity). Social and Behavioral constraints are caused by social norms, behaviors, preferences, or attitudes (specifically, disinterest in or skepticism of NCS), lack of social learning or exchange networks, discrimination of certain groups, disagreement among actors or groups, or equity concerns. Policies and Rules comprise formal or legal principles that negatively affect NCS implementation such as a lack or ineffectiveness of laws, policies or regulations; lack of tenure security; insecure, uncertain or inequitable benefit sharing; or incentives for competing land uses. Governments and Organizations can constrain NCS implementation through their behaviors (lack of political will; corruption or lack of transparency) or a lack of administrative (lack of enforcement of laws, policies, or regulations) or coordination capacity of state or nonstate entities, or their inability to prevent violent conflict or civil unrest. Material Inputs constraints arise from lack of needed NCS production inputs (e.g. seedlings, fertilizer), infrastructure (e.g. for irrigation), labor, water, or suitable land. Finally, negative side effects can constrain NCS implementation through competition with other land uses (excluding agriculture, tradeoffs with which are captured in the Economic category) or other negative side effects (Table S1). Despite some differences in the categorization and naming of select constraints, our classification is well aligned with existing ones (Table S2).

Data analysis was conducted in R. Avoided conversion and restoration pathways were combined for wetlands, grasslands, and peatlands, respectively, for most analysis due to limited data. Countries were aggregated by SDG Region, UN Subregion, and World Bank Income Group to examine trends across policy-relevant groupings and avoid small sample biases. SDG region refers to the regional groupings of countries used in the SDG report and statistical annex (93). UN subregion is a further disaggregated grouping of countries used by the United Nations Statistics Division (94). To compare constraint similarities within groupings, we used Jaccard similarity, Euclidean distance, and PERMANOVA analyses. Jaccard similarity focuses on the shared presence–absence of constraints (referred to as “constraint presence”), while Euclidean distance accounts for the shares of each constraint in the total constraint observations count for each pathway or geographic grouping (referred to as “constraint share”). We identified the similarity in constraint presence across geographies and pathways using a Jaccard similarity coefficient and similarity in constraint shares using Euclidean distance. Rankings of co-occurrence of constraints within subregions were assessed using Jaccard similarity (see Supplementary material for detailed methods).

## Supplementary Material

pgaf173_Supplementary_Data

## Data Availability

Our dataset is available on Harvard Dataverse at https://doi.org/10.7910/DVN/NEYIPD (33). The R code used to create the figures in the main text and Supplementary material has also been deposited in the Dataverse.

## References

[1] Griscom BW, et al 2017. Natural climate solutions. Proc Natl Acad Sci U S A. 114:11645–11650. 29078344 10.1073/pnas.1710465114 PMC5676916

[2] Ellis PW, et al 2024. The principles of natural climate solutions. Nat Commun. 15:547. 38263156 10.1038/s41467-023-44425-2 PMC10805724

[3] Chausson A, et al 2020. Mapping the effectiveness of nature-based solutions for climate change adaptation. Glob Chang Biol. 26:6134–6155. 32906226 10.1111/gcb.15310

[4] Seddon N, et al 2021. Getting the message right on nature-based solutions to climate change. Glob Chang Biol. 27:1518–1546. 33522071 10.1111/gcb.15513

[5] Seddon N . 2022. Harnessing the potential of nature-based solutions for mitigating and adapting to climate change. Science. 376:1410–1416. 35737796 10.1126/science.abn9668

[6] Stanturf JA, Mansourian S. 2020. Forest landscape restoration: state of play. R Soc Open Sci. 7:201218. 33489272 10.1098/rsos.201218 PMC7813234

[7] Seddon N, et al 2020. Understanding the value and limits of nature-based solutions to climate change and other global challenges. Phil Trans R Soc B Biol Sci. 375:20190120. 10.1098/rstb.2019.0120 PMC7017763 31983344

[8] Nabuurs G-J, et al Agriculture, forestry and other land uses (AFOLU). In: Shukla PR, et al, editors. IPCC, 2022: climate change 2022: mitigation of climate change. Contribution of working group III to the sixth assessment report of the intergovernmental panel on climate change. Cambridge University Press, Cambridge, UK and New York, NY, 2022. doi: 10.1017/9781009157926.009.

[9] Fagan ME, Reid JL, Holland MB, Drew JG, Zahawi RA. 2020. How feasible are global forest restoration commitments? Conserv Lett. 13:e12700.

[10] Fransen T, et al 2023. Taking stock of the implementation gap in climate policy. Nat Clim Chang. 13:752–755.

[11] Mo L, et al 2023. Integrated global assessment of the natural forest carbon potential. Nature. 624:92–101. 37957399 10.1038/s41586-023-06723-z PMC10700142

[12] Naturebase . Global NCS opportunity. 2024. [accessed 2024 Sept 18]. https://app.naturebase.org/map.

[13] Strassburg BBN, et al 2019. Strategic approaches to restoring ecosystems can triple conservation gains and halve costs. Nat Ecol Evol. 3:62–70. 30568285 10.1038/s41559-018-0743-8

[14] Griscom BW, et al 2020. National mitigation potential from natural climate solutions in the tropics. Phil Trans R Soc B Biol Sci. 375:20190126. 10.1098/rstb.2019.0126 PMC7017762 31983330

[15] Mappin B, et al 2019. Restoration priorities to achieve the global protected area target. Conserv Lett. 12:e12646.

[16] Naidoo R, et al 2008. Global mapping of ecosystem services and conservation priorities. Proc Natl Acad Sci U S A. 105:9495–9500. 18621701 10.1073/pnas.0707823105 PMC2474481

[17] Walker WS, et al 2022. The global potential for increased storage of carbon on land. Proc Natl Acad Sci U S A. 119:e2111312119. 35639697 10.1073/pnas.2111312119 PMC9191349

[18] Brancalion PHS, Holl KD. 2020. Guidance for successful tree planting initiatives. J Appl Ecol. 57:2349–2361.

[19] Karki L, et al 2023. Potentials and barriers to land-based mitigation technologies and practices (LMTs)—a review. Environ Res Lett. 18:093003.

[20] Nolan CJ, Field CB, Mach KJ. 2021. Constraints and enablers for increasing carbon storage in the terrestrial biosphere. Nat Rev Earth Environ. 2:436–446.

[21] Marshall AR, et al 2023. Fifteen essential science advances needed for effective restoration of the world's forest landscapes. Phil Trans R Soc B Biol Sci. 378:20210065. 10.1098/rstb.2021.0065 PMC9661955 36373922

[22] Schulte I, Eggers J, Nielsen JØ, Fuss S. 2022. What influences the implementation of natural climate solutions? A systematic map and review of the evidence. Environ Res Lett. 17:013002.

[23] Zeng Y, et al 2020. Economic and social constraints on reforestation for climate mitigation in Southeast Asia. Nat Clim Chang. 10:842–844.

[24] Asamoah EF, Maina JM. 2022. Nature-based climate solutions require a mix of socioeconomic and governance attributes. iScience. 25:105699. 36567709 10.1016/j.isci.2022.105699 PMC9768352

[25] Walker SE, et al 2024. Unintended consequences of nature-based solutions: social equity and flood buyouts. PLOS Clim. 3:e0000328.

[26] Schleicher J, et al 2019. Protecting half of the planet could directly affect over one billion people. Nat Sustain. 2:1094–1096.

[27] Erbaugh JT, et al 2020. Global forest restoration and the importance of prioritizing local communities. Nat Ecol Evol. 4:1472–1476. 32839542 10.1038/s41559-020-01282-2

[28] Fleischman F, et al 2020. Pitfalls of tree planting show why we need people-centered natural climate solutions. BioScience. 70:947–950.

[29] Tschora H, Cherubini F. 2020. Co-benefits and trade-offs of agroforestry for climate change mitigation and other sustainability goals in West Africa. Glob Ecol Conserv. 22:e00919.

[30] Tranchina M, Reubens B, Frey M, Mele M, Mantino A. 2024. What challenges impede the adoption of agroforestry practices? A global perspective through a systematic literature review. Agrofor Syst. 98:1817–1837.

[31] Pörtner H-O, et al 2023. Overcoming the coupled climate and biodiversity crises and their societal impacts. Science. 380:eabl4881. 37079687 10.1126/science.abl4881

[32] Naturebase . All NCS pathways v1. 2023. https://naturebase.org.

[33] Brumberg H, et al Global constraints to natural climate solution implementation. Harvard Dataverse, V3 [accessed 2024 Sept 23]. 10.7910/DVN/NEYIPD.

[34] Sasaki N . 2021. Timber production and carbon emission reductions through improved forest management and substitution of fossil fuels with wood biomass. Resour Conserv Recycl. 173:105737.

[35] Gladkikh TM, Collazo JA, Torres-Abreu A, Reyes AM, Molina M. 2020. Factors that influence participation of Puerto Rican coffee farmers in conservation programs. Conserv Sci Pract. 2:e172.

[36] dos Reis JC, et al 2020. Assessing the economic viability of integrated crop–livestock systems in Mato Grosso, Brazil. Renew Agric Food Syst. 35:631–642.

[37] Medeiros G, Florindo T, Talamini E, Neto AF, Ruviaro C. 2020. Optimising tree plantation land use in Brazil by analysing trade-offs between economic and environmental factors using multi-objective programming. Forests. 11:723.

[38] Chang K, Andersson KP. 2021. Contextual factors that enable forest users to engage in tree-planting for forest restoration. Land Use Policy. 104:104017.

[39] Ding Z, Yao S. 2021. Ecological effectiveness of payment for ecosystem services to identify incentive priority areas: sloping land conversion program in China. Land Use Policy. 104:105350.

[40] Commender KE, Munsell JF, Ares A, Sullivan BJ, Chamberlain JL. 2020. The effects of cost-share participant experience on forest buffer retention. Small-Scale For. 19:253–273.

[41] Wijsman K, et al 2021. Operationalizing resilience: co-creating a framework to monitor hard, natural, and nature-based shoreline features in New York state. Ecol Soc. 26.

[42] Raupp PP, et al 2020. Direct seeding reduces the costs of tree planting for forest and savanna restoration. Ecol Eng. 148:105788.

[43] Jalil A, Yesi Y, Sugiyanto S, Puspitaloka D, Purnomo H. 2021. The role of social capital of Riau women farmer groups in building collective action for tropical peatland restoration. For Soc. 5:341–351.

[44] Cao S, Suo X, Xia C. 2020. Payoff from afforestation under the Three-North Shelter Forest Program. J Clean Prod. 256:120461.

[45] Ma Z, Xia C, Cao S. 2020. Cost–benefit analysis of China's natural forest conservation program. J Nat Conserv. 55:125818.

[46] Austin KG, et al 2020. The economic costs of planting, preserving, and managing the world's forests to mitigate climate change. Nat Commun. 11:5946. 33262324 10.1038/s41467-020-19578-z PMC7708837

[47] Owusu V, Akoto-Adjepong V, Acheampong E, Barnes VR. 2022. Farmer perceptions and economic performance of cocoa agroforestry shade levels in Ghana. J Sustain For. 41:922–940.

[48] Löfqvist S, Garrett RD, Ghazoul J. 2023. Incentives and barriers to private finance for forest and landscape restoration. Nat Ecol Evol. 7:707–715. 37165107 10.1038/s41559-023-02037-5 PMC10172125

[49] Toxopeus H, Polzin F. 2021. Reviewing financing barriers and strategies for urban nature-based solutions. J Environ Manage. 289:112371. 33845267 10.1016/j.jenvman.2021.112371

[50] Calle A . 2020. Partnering with cattle ranchers for forest landscape restoration. Ambio. 49:593–604. 31292911 10.1007/s13280-019-01224-8 PMC6965560

[51] Roe S, et al 2021. Land-based measures to mitigate climate change: potential and feasibility by country. Glob Chang Biol. 27:6025–6058. 34636101 10.1111/gcb.15873 PMC9293189

[52] Schelhas J, Hitchner S, Dwivedi P, Thomas M. 2021. Understanding black landowner's engagement in forestry in Georgia, United States: a closer look. For Trees Livelihoods. 30:242–257.

[53] Kernecker M, Seufert V, Chapman M. 2021. Farmer-centered ecological intensification: using innovation characteristics to identify barriers and opportunities for a transition of agroecosystems towards sustainability. Agric Syst. 191:103142.

[54] Martín-Forés I, et al 2020. Spontaneous forest regrowth in South-West Europe: consequences for nature's contributions to people. People Nat. 2:980–994.

[55] Künzler D . 2022. Social policies driven by labour scarcity: colonial social policies in the concession economies of the United Nation subregion Middle Africa and their legacy. Glob Polit Econ. 1:238–256.

[56] Sachs J, et al Sustainable development report 2020: the sustainable development goals and covid-19 includes the SDG index and dashboards. Cambridge University Press, 2021.

[57] Blersch M, Keller J, Matusch T, Dannwolf L, Siegmund A. The network of UNESCO sites: changes and patterns visualised with cartograms. In: Martí-Henneberg J, editor. Creative ways to apply historical GIS. Springer, Cham, 2023. p. 181–195.

[58] Mehring M, Stoll-Kleemann S. 2008. Evaluation of major threats to forest biosphere reserves: a global view. GAIA - Ecol Perspect Sci Soc. 17:125–133.

[59] Hill MJ, Guerschman JP. 2022. Global trends in vegetation fractional cover: hotspots for change in bare soil and non-photosynthetic vegetation. Agric Ecosyst Environ. 324:107719.

[60] Eisenack K, et al 2014. Explaining and overcoming barriers to climate change adaptation. Nat Clim Chang. 4:867–872.

[61] Reyes R, Nelson H, Zerriffi H. 2021. How do decision makers´ ethnicity and religion influence the use of forests? Evidence from Chile. For Policy Econ. 128:102462.

[62] Gregorio N, Herbohn J, Tripoli R, Pasa A. 2020. A local initiative to achieve global forest and landscape restoration challenge—lessons learned from a community-based forest restoration project in Biliran Province, Philippines. Forests. 11:475.

[63] Gutiérrez-Zamora V, Estrada MH. 2020. Responsibilization and state territorialization: governing socio-territorial conflicts in community forestry in Mexico. For Policy Econ. 116:102188.

[64] von Kleist K, Herbohn J, Baynes J, Gregorio N. 2021. How improved governance can help achieve the biodiversity conservation goals of the Philippine National Greening Program. Land Use Policy. 104:104312.

[65] Chang CH, et al 2025. Global evidence of human well-being and biodiversity impacts of natural climate solutions. Nat Sustain. 8:75–85.

[66] Bhattarai S, Pant B, Laudari HK, Rai RK, Mukul SA. 2021. Strategic pathways to scale up forest and landscape restoration: insights from Nepal's Tarai. Sustainability. 13:5237.

[67] van der Meer Simo A, Kanowski P, Barney K. 2020. The role of agroforestry in swidden transitions: a case study in the context of customary land tenure in Central Lao PDR. Agrofor Syst. 94:1929–1944.

[68] Zepharovich E, Ceddia MG, Rist S. 2021. Social multi-criteria evaluation of land-use scenarios in the Chaco Salteño: complementing the three-pillar sustainability approach with environmental justice. Land Use Policy. 101:105175.

[69] Vetter S . 2020. With power comes responsibility – a rangelands perspective on forest landscape restoration. Front Sustain Food Syst. 4:1–10.

[70] Pichler M, Bhan M, Gingrich S. 2021. The social and ecological costs of reforestation. Territorialization and industrialization of land use accompany forest transitions in Southeast Asia. Land Use Policy. 101:105180.

[71] Brownson K, et al 2020. Governance of Payments for Ecosystem services influences social and environmental outcomes in Costa Rica. Ecol Econ. 174:106659.

[72] Vincent K, Daly M, Scannell C, Leathes B. 2018. What can climate services learn from theory and practice of co-production? Clim Serv. 12:48–58.

[73] Cash DW, et al 2003. Knowledge systems for sustainable development. Proc Natl Acad Sci U S A. 100:8086–8091. 12777623 10.1073/pnas.1231332100 PMC166186

[74] Erbaugh JT, Nurrochmat DR. 2019. Paradigm shift and business as usual through policy layering: forest-related policy change in Indonesia (1999–2016). Land Use Policy. 86:136–146.

[75] Hansen OW, van den Bergh J. 2024. Environmental problem shifting from climate change mitigation: a mapping review. PNAS Nexus. 3:pgad448. 38205028 10.1093/pnasnexus/pgad448 PMC10776357

[76] Lieffers VJ, Pinno BD, Beverly JL, Thomas BR, Nock C. 2020. Reforestation policy has constrained options for managing risks on public forests. Can J For Res. 50:855–861.

[77] Waring BG, Gurgel A, Köberle AC, Paltsev S, Rogelj J. 2023. Natural Climate Solutions must embrace multiple perspectives to ensure synergy with sustainable development. Front Clim. 5:01–07.

[78] Loisel J, et al 2021. Expert assessment of future vulnerability of the global peatland carbon sink. Nat Clim Chang. 11:70–77.

[79] Costanza R, et al 2014. Changes in the global value of ecosystem services. Glob Environ Change. 26:152–158.

[80] Xu J, Morris PJ, Liu J, Holden J. 2018. PEATMAP: refining estimates of global peatland distribution based on a meta-analysis. Catena (Amst). 160:134–140.

[81] Zhao Q, et al 2016. A review of methodologies and success indicators for coastal wetland restoration. Ecol Indic. 60:442–452.

[82] Rana P, et al 2024. Cost-efficient management of peatland to enhance biodiversity in Finland. Sci Rep. 14:2489. 38291097 10.1038/s41598-024-52964-x PMC10827728

[83] Buisson E, et al 2021. Key issues in Northwestern Mediterranean dry grassland restoration. Restor Ecol. 29:e13258.

[84] Lee KN, Lawrence J. 1986. Adaptive management: learning from the Columbia river basin fish and wildlife program. Environ Law. 16:431–460.

[85] Williams BK, Brown ED. 2014. Adaptive management: from more talk to real action. Environ Manage. 53:465–479. 24271618 10.1007/s00267-013-0205-7 PMC4544568

[86] Albert C, et al 2021. Planning nature-based solutions: principles, steps, and insights. Ambio. 50:1446–1461. 33058009 10.1007/s13280-020-01365-1 PMC8249551

[87] Manner J, Ersson BT. 2021. Mechanized tree planting in Nordic forestry: simulating a machine concept for continuously advancing site preparation and planting. J For Sci. 67:242–246.

[88] Yao RT, Palmer DJ, Payn TW, Strang S, Maunder C. 2021. Assessing the broader value of planted forests to inform forest management decisions. Forests. 12:662.

[89] Do H, Luedeling E, Whitney C. 2020. Decision analysis of agroforestry options reveals adoption risks for resource-poor farmers. Agron Sustain Dev. 40:1–12.

[90] Moser SC, Ekstrom JA. 2010. A framework to diagnose barriers to climate change adaptation. Proc Natl Acad Sci U S A. 107:22026–22031. 21135232 10.1073/pnas.1007887107 PMC3009757

[91] Berger R . 2015. Now I see it, now I don’t: researcher's position and reflexivity in qualitative research. Qual Res. 15:219–234.

[92] Wallace BC, Small K, Brodley CE, Lau J, Trikalinos TA. Deploying an interactive machine learning system in an evidence-based practice center: abstrackr. 2012. Proceedings of the 2nd ACM SIGHIT International Health Informatics Symposium, IHI ‘12. Association for Computing Machinery. p. 819–824.

[93] United Nations. SDG indicators. [accessed 2024 Sept 18]. https://unstats.un.org/sdgs/indicators/regional-groups/.

[94] United Nations Statistics Division . Geographic regions. [accessed 2024 Sept 18]. https://unstats.un.org/unsd/methodology/m49/.

