# Supplementary material for: Global analysis of constraints to natural climate solution implementation
Source: PNAS Nexus. 2025 Jun 24;4(6):pgaf173. doi: 10.1093/pnasnexus/pgaf173 (PMC12199758; doi:10.1093/pnasnexus/pgaf173)
Supplement: pgaf173_Supplementary_Data [file pgaf173_supplementary_data.zip › PNASNEXUS-PNASNEXUS-2024-01320R-s01.pdf]

Supplementary Information for

Global Analysis of Constraints to Natural Climate Solution Implementation

Hilary Brumberg[1,2,3]*, Margaret Hegwood[1,4], Waverly Eichhorst[1], Anna LoPresti[5], James T. Erbaugh[6,7], Timm Kroeger[6]

Hilary Brumberg
Email: hbrum@stanford.edu

This PDF file includes:

Supplementary Text
Figures S1-S12
Tables S1-S4
Legend for Dataset S1
SI References

Other supplementary materials for this manuscript include the following:

Dataset S1

Supplementary Information Text

Full methods

Search for relevant literature

We conducted literature searches for eleven NCS pathways using the Scopus and Web of Science databases on November 4, 2021. The eleven pathways included agroforestry, avoided forest conversion, avoided coastal wetland conversion, coastal wetland restoration, avoided grassland conversion, grassland restoration, avoided peatland conversion, peatland restoration, reforestation, climate-smart forestry, and regenerative agriculture. We conducted one search per NCS pathway in each database, except as follows. We conducted combined searches for the following pathway pairs, due to the low volume of studies on each: avoided coastal wetland conversion and coastal wetland restoration, avoided grassland conversion and grassland restoration, and avoided peatland conversion and peatland restoration. We also searched for natural-climate solutions and synonyms or related terms (e.g. nature-based solutions) for nine unique searches overall. Searches were conducted using a list of synonyms for each NCS pathway paired with the term “constraint” and synonyms (e.g., challenges, barriers) for implementing the studied NCS pathways. The search terms used were deliberately chosen to return studies that may not use an explicit NCS or Nature-based Solutions (NbS) framing. Additionally, some papers that may assess NCS implementation constraints mention neither NCS, NbS, nor specific pathway names in the title, abstract or keywords (e.g., a study with an integrated crop-livestock system (ICLS) framing may not mention the word “agroforestry,” but some of the specific constraints documented in the study may be relevant to the agroforestry pathway). Thus, we used search terms that described alternative NCS pathway names in addition to biome names (e.g., “forest”) and major activity types (e.g., “protect, conserve, avoid, restore, manage, rehabilitate, regenerate”). To do this we used search terms related to activities specific to particular biomes (e.g., forest “degradation” “preservation”, or “conversion”) and included sub-pathway and/or alternative descriptors. For example, rather than only searching for “wetland” or even “peatland”, we also included specific descriptors such as “fen”, “bog” or peatlands and “mangrove”, “sea grass”, and “salt marsh” for wetlands. We specified no time period of interest, and the search was conducted in English. A full list of terms used to conduct each search can be found in the “Search terms” section below. Figure S1 shows a flow diagram detailing the steps taken for the literature review and the number of papers in each step.

Pre-screening

Our search returned 26,432 unique publications (Table S1). Before pre-screening, we removed papers returned from the regenerative agriculture (RA) search, which represented 24% (n = 6,387) of the total papers returned from our search. Given the large number of RA articles, we concluded that the scope of the regenerative agriculture literature warranted a separate, future study. Note that papers returned by both the RA searches and searches of other pathways (e.g., agroforestry) were retained in our sample.

The remaining 20,045 studies returned from the search were pre-screened based on paper titles and abstracts to determine whether they met the two key inclusion criteria for our study: 1) the explicit mention of one of the studied NCS pathways, and 2) an indication that the study included information about constraints to NCS implementation. We define “constraints to NCS implementation” as factors that prevent NCS adoption, implementation, performance, evaluation, or permanence. For example, a funding constraint may not prevent the adoption of agroforestry as a practice but may limit effective project design and implementation. Papers were then coded according to the aforementioned criteria as either a “1” (include in analysis), “2” (potentially include in analysis), or “0” (exclude from analysis). Papers labeled as “1” met both inclusion criteria. Papers labeled as “2” lacked clear mention of an NCS pathway and/or constraints, but used terminology (e.g., climate-smart agriculture) that indicated the study may be relevant. Papers that met neither of these criteria were labeled as a “0”.

HB, WE, MH, and AL first manually screened 3,005 abstracts in Excel, and then screened the majority of the remaining abstracts using the machine learning tool Abstrackr (1), which uses real-time screening

results to predict abstract relevance based on the screening criteria. As a result, Abstrackr presents the abstracts predicted to be most relevant to the screener first, while less relevant abstracts are given lower priority in the database. Abstrackr also provides an estimate for the number of remaining abstracts that are likely to be relevant. We stopped the pre-screening process when Abstrackr predicted that none of the remaining abstracts (n = 4,280) would be relevant, the likelihood of inclusion within Abstrackr was 0.325 or less, and after each team member screened out 50 articles in a row.

We screened (excluding Abstrackr's rejected predictions) a total of 15,201 articles: 3,005 (19.7%) manually and 12,206 (80.3%) with Abstrackr. Of the screened papers, 4,299 papers were accepted for full screening (3,329 labeled as accept, 970 publications labeled as maybe accept). 11,466 publications were rejected for full screening. This resulted in a pre-screening acceptance rate of 28.2% (accepted abstracts as a percentage of screened abstracts).

Intercoder reliability during pre-screening

We conducted two rounds of intercoder reliability tests during pre-screening where each screener (HB, WE, MH, and AL) individually reviewed 100 papers. Average agreement was determined for each round of intercoder reliability testing, and coding differences were discussed by the group. In Round 1, the average agreement between screeners (defined as consensus among ≥ 3 screeners) was 81.3%. In Round 2, the average agreement between reviewers was 90.5% (Table S4). These results indicate that our results were consistent across pre-screeners.

Full coding

The 4,299 papers identified for full screening were published between 1961 and 2021. Since constraints may change over time and thus constraints reported in older papers may no longer apply, we decided to focus our full screening on the two most recent years included in our literature search (2020 and 2021). This subset (1,821 papers) accounted for 42.3% of all papers published during 1961-2021 initially identified for full screening.

We completed the full screen of 1,821 papers as follows. First, we developed a list of desired information to be collected from each article. Second, we tested this initial list by coding two publications collectively and adjusting the coding variables accordingly. This process also allowed for consistency among screeners when conducting the full screening. The final codebook contains 43 variables (see Dataset S1) which include information on NCS pathway, sub-pathway, implementers, geography, constraint, affected stakeholders, solutions, and solution costs.

Next, HB, WE, MH, and AL were randomly assigned the remaining articles for full screening. Depending on their length and complexity, articles took anywhere from 20 minutes to 3 hours to full screen for the presence of constraints and corresponding information. About 19% (352 of 1,821 papers) of fully screened publications contained information about constraints to NCS and were fully coded in accordance with the codebook. 151 of papers (~8 percent) were identified as review papers and set aside for future analysis.

Constraint observation and categorization

During the full screening process, information was collected on constraints from journal articles. Any information about a unique constraint–what we call a "constraint observation"–was recorded and related information was collected. A unique constraint observation is a constraint that affects one pathway in one country (i.e., constraint by pathway by country). For example, a reforestation project in Brazil that lacks funding necessary for successful implementation is reported as one constraint. This definition also implies that a single journal article may have multiple unique constraint observations even if only one constraint (e.g. skepticism) is discussed if that constraint applies to multiple pathways, multiple countries, or both. Overall, we recorded 2,480 constraint observations.

A constraint observation (our raw data) was then categorized into one of 39 constraints (Table S1). Throughout this paper, the term "constraint" refers to any of the 39 unique constraints that we developed through emergent coding. Each of these constraints was developed iteratively to capture the diverse

observations across journal articles that used different language to describe the same constraint. For example, one study might note that funds for reforestation are limited whereas another study notes that there is not enough cash directed towards reforestation. These are both categorized as the "Lack of funding" constraint. Due to differences in the description and interpretation of the observed constraints, the classifications of all observed constraints were reviewed by at least three authors for consistency. We then grouped the constraints into seven categories: Economic, Governments and Organizations, Knowledge, Material Inputs, Policies and Rules, Social and Behavioral, and Negative Side Effects (Table S2).

The literature on NCS constraints is inconsistent in both terminology and categorization. For example, what we term “constraint categories” is variously referred to as “feasibility dimensions" (2), "barriers and opportunities" (3), "categories of enabling factors" (4) or "barriers" (5), to name but a few. The classification of individual constraints into these categories and the thematic scope and terminology used for the categories themselves also show a considerable degree of diversity (Table S2). For example, Karki et al. (2023) classify “lack of credit access" as a "technological" barrier, while Schulte et al. (2021) classify the somewhat broader "financial services" as a ‘financial" enabling factor, and IPCC AR6 (2021) classifies "scale and accessibility of financing" as a "socio-economic" barrier or opportunity.

Below, we discuss how our constraint classification differs from those used in four other recent NCS studies (2–5). Table S2 identifies congruence between individual constraints in our and the other classifications.

As can be seen in Table S2, there is broad alignment in terminology between our constraint categories and those of the other four studies. One difference is that we refer to "knowledge" rather than "technological" or "technical" constraints because this term better captures the informational nature of some of the included constraints (e.g., lack of knowledge about NCS design, management, or performance; either in general or on the part of land managers). Furthermore, like Schulte et al. (2021) but unlike IPCC (2021 [AR6]), Karki et al. (2023) and Roe et al. (2021), we distinguish between the formal or informal institutional frameworks of policies, laws, rules, and regulations that affect NCS implementation (our Policies & Rules category), and the attitudes, behaviors or capacities of governmental or non-governmental entities (Governments & Organizations category). We believe this distinction is useful and important as the actions needed to overcome these two categories of constraints are fundamentally different. For example, constraints within our Policies & Rules category may require a focus on legislative efforts or other modes of rule-making, while constraints within the Governments & Organizations category may require improved capacity to enforce pre-existing rules or coordinate actions among different offices, agencies or departments.

We do not discuss in detail the agreement or differences between each of our constraints and specific constraints in the other classifications. Rather, when one of our constraints is identical with, or substantively similar to, specific constraints in the other classifications, this is indicated by the same Arabic numerals (shown in parentheses) in Table S2. Where there is limited congruence, this is indicated by italicized Arabic numerals.

Schulte et al. (2021) categorize NCS "enabling factors" based on IPCC (2014) (6). While most of the "enabling factors" identified in Schulte et al. (2021) are reflected in our constraints, some are not. The latter is due primarily to two factors: a substantive difference between Schulte et al.’s (2021) “enabling factors” and our constraints, and differences in the parsing of constraints into their constituent components or sub-constraints.

Enabling factors as defined in Schulte et al. (2021) are conditions that are positively correlated with NCS implementation in the literature. In contrast, our constraints are factors that in the literature are reported as impeding NCS implementation. While in many cases Schulte et al.'s (2021) "enabling factors" represent the inverse of constraints, this is not always true. For example, our classification does not include three of Schulte et al.'s (2021) biophysical enabling factors (favorable climatic conditions, species diversity, and carbon stock potential). Since enabling factors as defined in Schulte et al. (2021) are conditions that are positively correlated with NCS implementation, it is not surprising that, for example, high potential carbon stocks or high biodiversity are positively correlated with NCS because of the potential income they might generate (from carbon) or the financing they might attract (for biodiversity). However, lower potential carbon stocks are not necessarily a constraint on NCS implementation, because an NCS may deliver other desired outputs for potential implementers. Where

low carbon stock potential is indeed identified in a paper as a constraint on NCS implementation, its negative effect on NCS implementation is captured by our constraints related to carbon market access or carbon prices. The same applies to biodiversity, lower levels of which may not necessarily prevent the attractiveness of an NCS to potential adopters – say, farmers or ranchers who consider agroforestry adoption for productivity gains, increased climate resilience, or carbon income. Where low biodiversity prevents the feasibility of an NCS project, it will do so via lack of access to biodiversity markets or low prices on those markets, two constraints we capture separately.

An example of a difference in parsing of constraints is Schulte et al.'s (2021) "delivery of benefits" as an enabling factor, where delivery of benefits is defined as the sufficient size of overall benefits, or net welfare (i.e., not just financial) gains (4). We do not have a corresponding inverse constraint "lack of benefits" or "insufficient benefits." Rather, we have separate constraints for factors that individually or in combination may result in insufficient benefits, such as high implementation costs; inadequate markets or prices for NCS products, biodiversity conservation, or ecosystem services; human-wildlife conflict; tradeoffs with agricultural yield or profits; tradeoffs with other uses; or other negative side effects (e.g., perceived or actual health or property damage or invasive species as a result of NCS).

We do not have direct equivalents for Schulte et al.'s (2021) social NCS enabling factors of IPLC engagement, stakeholder consultation, recognition of traditional values, and FPIC. Presence of these enabling factors during project design and implementation ensures appropriate local consultation and increases the likelihood that project designs meet local desires and preferences. While we do not explicitly account for the lack of these factors as constraints, their absence is likely to result in several of the constraints in our Social & Behavioral category—specifically, preferences or norms favoring non-NCS land uses; disinterest or skepticism of NCS or lack of trust in NCS promoters; lack of opportunity to participate in or influence the implementation of NCS; and concerns over negative equity impacts.

Finally, there are some differences in categorizations between Schulte et al. (2021) and our analysis. For example, Schulte et al. (2021) classify lack of availability of qualified government personnel as an economic constraint (as explained in their paper, this is included as part of their "labor availability" constraint). We classify lack of government capacity under our Government & Organizations category, specifically, the constraint "lack of administrative capacity."

The IPCC's (2021) "socio-economic barriers and opportunities" category includes finance mechanisms, funding, and incentives, which we capture in our economic category; risk and uncertainty, which we capture in our knowledge and economic categories; impacts on poverty or food security, the latter of which we capture in our Economic and Negative Side Effects categories; and cultural values and social acceptance, which we bin into our Social & Behavioral category. IPCC's institutional barriers and opportunities include transparent and accountable governance (accounted for in our Government & Organizations category), clear land tenure and land-use rights (our Policies & Rules category), and institutional capacity (our Government & Organizations category). IPCC's ecological barriers and opportunities category includes land and water availability (in our Material Inputs category); uncertainties and differences in outcomes related to specific soil conditions, water availability, GHG reduction potential as well as natural variability and resilience (captured to various degrees in our Knowledge category), and adaptation and biodiversity benefits (captured in our Knowledge category). Their technological barriers and opportunities category consists of monitoring, reporting, and verification (MRV) needs, which we do not capture as a separate constraint. However, we capture the costs associated with MRV in our Economic category, and the challenges in quantification in our Knowledge category. In general, our larger number of constraints is largely due to our finer parsing of constraints.

Karki et al. (2023) identify socio-cultural "barriers" that comprise norms and values, knowledge and perception, and behavior "sub-barriers," with "descriptions" of specific finer barriers within each sub-barrier. These barriers generally are captured in the constraints in our Social & Behavioral and Knowledge categories. Karki et al.'s (2023) technological barriers include complexity (difficult to adopt activity; requiring high management skills), resources (access to specialized machinery, lack of transport infrastructure, lack of inputs, limited/no access to credit, limited extension facilities); and development (lack of MRV; larger uncertainties about benefits, limited understanding of land suitability; technological readiness). These are captured in our Economic, Knowledge, and Material Inputs categories. Karki et al.'s (2023) economic barriers include costs (unable to afford specialized machinery; large initial investment, expensive to deploy at a scale where there is large potential), income (potential for income decline due to tradeoffs; transitional period with higher production costs and lower income;

lack of incentives), and value (difficulty of monetizing non-market benefits/ES), which are captured in our Economic category. Their institutional barriers include policy barriers (lack of policy support mechanism to set explicit incentives; lack of policy implementation; disinterest of policymakers) that we capture in our Governments & Organizations and Policies & Rules categories; governance barriers (lack of cross-sectoral responsibility sharing; top-down approach; coordination between stakeholders, lack of proper monitoring) some of which we capture in our Governments & Organizations category; and regulation-related barriers (counter-productive public policies and legislation; lack of standards and protocols to measure C sequestration) that we capture in our Policies & Rules category. Karki et al.'s (2023) ethical barriers comprise challenges related to conflict (risks of land grabbing; equitable benefit sharing; social conflicts) and fairness (limited access of women and minority groups to resources and land; no consideration of the rights of Indigenous People and local communities) that we capture in our Social & Behavioral and Policies & Rules categories, and tradeoffs (land availability and competition with other land uses; possible increase in food prices and compromise food security; negative effect on the environment), which we capture in our Negative Side Effects category.

Roe et al. (2021) identified macro-level "feasibility indicators" to assess the country-level feasibility of terrestrial NCS for the world. Unavoidably, this resulted in the selection of indicators characterized by geographically broad data availability. Some of Roe et al.'s (2021) indicators are fairly similar to ours, while others represent country-wide characteristics that arguably are positively related to NCS feasibility but whose absence does not directly translate into specific constraints (e.g., GDP per capita, agricultural value added, personal rights, agricultural total factor productivity, or ease of doing business).

Data analysis

The final dataset is available on Harvard Dataverse (7). Data analysis was conducted in R. Since there were fewer data on wetland, grassland, and peatland ecosystems, the avoided conversion and restoration pathways for each of these ecosystems were combined into avoided wetland coastal conversion and coastal wetland restoration; avoided grassland conversion and grassland restoration; and avoided peatland conversion and peatland restoration, respectively, for most analyses. The only analyses in which these pathways are not combined are for the Jaccard similarity and Euclidean distances for pathway constraints; in these cases, they are referred to as “disaggregated pathways.”

We also aggregated the countries in three different ways for different parts of the analysis: SDG region, UN subregion, and World Bank Income Group. We aggregated for two reasons: 1) to avoid drawing conclusions based on small sample sizes due to limited data availability in each country, and b) to see how trends in constraints varied across these groupings which are often used for policies and other interventions. SDG region refers to the regional groupings of countries used in the SDG report and statistical annex (8). UN subregion is a further disaggregated grouping of countries used by the United Nations Statistics Division (9). These regions originate from continental groupings and are divided into subregions and intermediary regions to promote consistency in population size, demographic characteristics, and statistical accuracy. In the article text, “region” always refers to SDG regions and “subregion” always refers to UN subregions. The constraint distributions by SDG region and UN subregion are including the main text, and World Bank Income Groups are included in the SI (Figure S2).

We compared the rankings of constraint and constraint category observations across all countries (Figures 2A and 2C) with different subsets that may be useful to readers and as sensitivity analysis (Figures S3-S7). Figure S3 provides the number of unique countries for each constraint was observed, to suggest how widespread each constraint was observed. Figure S4 provides the ranking of constraints just for the 10% of countries (21 countries) with the highest biophysical NCS CMP, which together comprise 68% of the world's CMP (10). Figures S5 and S6 provide the distribution of constraints and constraint categories, respectively, observed in the five countries with the most observations in our dataset (Brazil, United States of America, Colombia, Peru, and Nepal) and for all countries except these five. Figure S7 provides the total CMP potentially affected by each constraint (Mt CO_2e), calculated by simply adding up the full CMP of each country for which a constraint was observed. While this could be interpreted as a proxy for the potential impact of each constraint, it is an extreme oversimplification and makes many assumptions and thus should be interpreted with a high degree of caution. For instance, such an analysis does not consider potential interrelations among constraints and may be interpreted

as suggesting that elimination of a particular constraint could unlock the CMP shown for the constraint. Moreover, some constraints may only affect a portion of a country. Also, constraints may reduce CMP to different degrees; some constraints may fully prevent NCS implementation, while others may merely slow the scaling or reduce impact. We provide this figure here in the SI in case it can be of use to any readers, but it should also be interpreted carefully and always accompanied by the assumptions and limitations.

To help determine whether aggregating the countries was appropriate, we conducted Jaccard similarity, Euclidean distance, and PERMANOVA analyses to identify whether countries tended to have more similar constraints to other countries in their same SDG region and UN subregion. We used both Jaccard similarity and Euclidean distance to capture different aspects of constraint similarities as a robustness check. Jaccard similarity focuses on the shared presence-absence of constraints (referred to as “constraint presence"), while Euclidean distance accounts for the proportional differences in constraint composition (referred to as “constraint share”). For Jaccard similarity, we created a binary presence-absence table for constraints in each country (constraint presence) and then calculated the Jaccard dissimilarity between each country. Then we converted the Jaccard distance matrix into a similarity matrix. For each country pair, we identified whether the two countries are in the same region (within-region) or in different regions (between-region). Euclidean distance was employed to quantify the differences in the proportion of each constraint across countries. We computed the proportion of each constraint relative to the total count of constraints found in each country (constraint shares). Using these proportions, we calculated the Euclidean distance between each pair of countries to measure dissimilarity. We then grouped the country pairs based on whether they were in the same region. The Jaccard similarity and Euclidean distances were used in PERMANOVA (Permutational Multivariate Analysis of Variance) to statistically evaluate whether the composition of country constraints differed significantly between regions. These analyses were completed separately to compare countries based on SDG region and then by UN subregion (Figure S8).

To compare the similarity of constraints among different pathways, we conducted both Jaccard and Euclidean similarity analyses. Disaggregated pathways were used for this analysis to compare constraints between the restoration and avoided conversation pathways for each ecosystem (ex. constraints observed for peatland restoration and avoided peatland conversion were disaggregated). For each disaggregated pathway in each subregion, we then constructed a binary presence-absence table for each constraint. This approach quantified the extent of shared constraints between disaggregated pathways within the same subregion. For each subregion, we calculated the Jaccard distance matrix and converted it to a similarity matrix by subtracting the distance values from one. We then determined the average Jaccard similarity across all subregions for each disaggregated pathway. For Euclidean distance, we computed the proportion of each constraint within each subregion-pathway combination. This proportional data was used to calculate Euclidean distance matrices, providing a measure of dissimilarity based on the relative abundance of constraints (constraint shares). These distances were converted to similarity matrices and averaged across subregions for each disaggregated pathway to estimate the average Euclidean similarity for each disaggregated pathway across subregions. Finally, heatmaps were generated to visualize the average Jaccard and Euclidean similarities of disaggregated pathways, highlighting which disaggregated pathways had the most similar constraints within subregions (Figures S9-10).

To identify co-occurrence of constraints within subregions, we used Jaccard similarity analysis. We constructed a binary presence-absence table that indicated the presence of each constraint in each subregion. Using this binary data, we calculated the Jaccard distance matrix to measure the dissimilarity between each pair of constraints, which we then converted into a similarity matrix. We then determined the constraint that exhibited the highest Jaccard similarity with each individual constraint, highlighting the pairs with the greatest similarity. For example, the constraint “Lack of information about co-benefits” demonstrated the greatest co-occurrence with “Lack of funding” ($J=0.76$), but “Lack of funding” demonstrated the greatest co-occurrence with “Lack of information about how to design or manage” ($J=0.89$). Using the pairwise Jaccard similarity coefficient data, we created a network graph to visualize asymmetrical pairwise relationships (Figure 2B). To aid readers in visualizing the relationship between different categorizations of constraints, we also created a fluvial plot (Figure S11).

To help practitioners identify co-occurring constraints and solutions at different phases of NCS implementation, we recommend considering constraints at each phase of the adaptive management framework. Figure S12 provides a visual to aid in this process, including examples of co-occurring

constraints at each phase (Figure S12A) and example guiding questions to identify constraints and solutions at each phase (Figure 12B). These are examples and are not intended to be exhaustive. Constraints from multiple categories are likely to be present at each phase of an NCS project, as illustrated by the inner circles in Figure S12A. In the Assess phase, constraints may limit the ability to diagnose the problem, identify the cultural and geographic context, engage key rightsholders and stakeholders, and account for existing initiatives, restricting NCS uptake. This paper contributes to the Assess phase by identifying existing implementation conditions at national and supra-national scales. The Design phase focuses on defining project scope and objectives, identifying strategies and alternatives, creating an operational plan, and anticipating potential impacts, but constraints at this phase may hinder feasibility, decision-making, and planning effectiveness. In the Implement phase, constraints may prevent full or effective application of NCS. Constraints to the Evaluate phase may inhibit the selection of appropriate indicators, the development of standardized monitoring processes, and the ability to assess and interpret project impacts. Finally, in the Adapt phase, constraints may limit the ability to update objectives and actions based on new knowledge and prevent adjustments that mitigate unintended negative consequences, affecting NCS permanence. While this framework describes an idealized implementation process, in practice, NCS implementation is often iterative, with projects engaging in only some phases or revisiting earlier ones as conditions evolve.

Search terms
Below are the search strings used for the literature searches on constraints to NCS feasibility.

[Sample: All NCS pathways]
“nature-based solutions” OR “natural climate solutions” OR “nature-based climate solutions”

[Sample: Avoided forest conversion]
“avoided forest loss” OR “avoided deforestation” OR “forest conservation” OR “forest protection” OR “avoided forest degradation” OR “avoided forest conversion” OR “forest preservation”

[Sample: Agroforestry]
“agro-forest*” OR “agroforest*” OR “woodlot” OR “silvopastur*” OR “silvo-pastur*” OR “agrisilvicultur*” OR “agri-silvicultur*” OR “agrosilvicultur*” OR “agro-silvicultur*” OR “tree-based intercropping” OR “windbreaks” OR “alley cropping” OR “alley-cropping” OR “improved fallow” OR “integrated crop-livestock-tree systems” OR “integrated crop-livestock systems” OR “integrated tree-livestock systems” OR “integrated tree-crop systems” OR “sustainable agriculture” OR “community forestry” OR “hedgerows” OR “riparian buffers”

[Sample: Reforestation]
“reforestation” OR “forest restoration” OR “forest landscape restoration” OR “timber plantations” OR “tree plantations” OR “mixed tree species plantations” OR “forest regrowth” OR “secondary forests” OR “tree planting” OR “afforestation” OR “forest regeneration” OR “forest cover increase” OR “forest-cover increase” OR “tree cover increase” OR “tree-cover increase” OR “forest recovery” OR “forest transition”

[Sample: Peatland protection and restoration]
“peatland* protection” OR “peatland* restoration” OR “peatland* rewetting” OR “peatland* wetting” OR “avoided peatland* conversion” OR “avoided peat* drainage” OR “avoided peat* fires” OR “avoided peatland subsidence” OR “peatland* conservation” OR “peatland* recovery” OR “bog protection” OR “bog restoration” OR “bog rewetting” OR “bog wetting” OR “avoided bog conversion” OR “bog recovery” OR “bog conservation” OR “bog recovery” OR “fen protection” OR “fen restoration” OR “fen rewetting” OR “fen wetting” OR “avoided fen conversion” OR “fen recovery” OR “fen conservation” OR “fen recovery” OR “avoided fen conversion” OR “brown carbon”

[Sample: Climate-smart forestry]
“Climate-smart forestry” OR “climate smart forestry” OR “ low-impact logging” OR “low impact logging” OR “reduced-impact logging “ OR “reduced impact logging” OR “low-impact timber production” OR “low impact timber production” OR “sustainable forestry” OR “low-impact forestry” OR “low impact forestry” OR “sustainable timber production” OR “sustainable logging” OR “extended rotation” OR “extended harvest rotation” OR “conservation forestry” OR “set-asides” OR “improved forest management” OR “improved natural forest management” OR “enhanced forest growth” OR “directional felling” OR “improved logging road construction” OR “improved forest road construction” OR “improved skidding” OR “reduced wood waste” OR (“reduced collateral damage” AND “forest*”)

[Sample: Grassland conservation]
“Grassland* protection” OR “avoided grassland conversion” OR “avoided grassland loss” OR “grassland conservation” OR “prairie conservation” OR “grassland restoration” OR “prairie restoration” OR “avoided prairie loss” OR “savannah restoration” OR “savannah protection” OR “avoided savannah conversion” OR “avoided savannah loss”

[Sample: Coastal wetland protection]
“coastal wetland protection” OR “coastal wetland restoration” OR “avoided coastal wetland conversion” OR “mangrove restoration” OR “mangrove protection” OR “avoided mangrove conversion” OR “avoided mangrove loss” OR “saltmarsh protection” OR “salt marsh protection” OR “salt-marsh protection” OR “avoided saltmarsh conversion” OR “avoided salt marsh conversion” OR “avoided salt-marsh conversion” OR “avoided saltmarsh loss” OR “avoided salt marsh loss” OR “avoided salt-marsh loss” OR “saltmarsh restoration” OR “salt marsh restoration” OR “salt-marsh restoration” OR “seagrass restoration” OR “seagrass protection” OR “sustainable shrimp farming” OR “sustainable aquaculture” OR “tidal marsh protection” OR “avoided tidal marsh conversion” OR “tidal marsh restoration” OR “avoided tidal marsh loss” OR “submerged aquatic vegetation restoration” OR “submerged aquatic vegetation protection” OR “avoided submerged aquatic vegetation conversion” OR “avoided submerged

aquatic vegetation loss” OR “sustainable kelp farming” OR “sustainable seaweed farming” OR “sustainable seaweed aquaculture” OR “tidal wetland protection” OR “tidal wetland restoration” OR “avoided tidal wetland conversion” OR “avoided tidal wetland loss”

[Sample: Regenerative agriculture Run 1]
“regenerative agriculture” OR “conservation agriculture” OR “sustainable agriculture” OR “organic agriculture” OR “sustainable farming” OR “organic farming” OR “reduced-till” OR “reduced till” OR “minimum till” OR “minimum-till” OR “no till” OR “no-till” OR “conservation tillage” OR “fertilizer management” OR “optimal fertilizer use” OR “integrated soil fertility management” OR “4R nutrient management” OR “soil fertility management” OR “sustainable intensification” OR “rice water management” OR “rice water conservation” OR “alternate wetting and drying” OR “water-saving rice irrigation” OR (“water-saving irrigation” AND “rice”) OR (“water-saving irrigation practices” AND “rice”) OR (“water-saving technologies” AND “rice”) OR (“water-saving practices” AND “rice”) OR (“water-saving methods” AND “rice”) OR “water-saving rice technologies” OR “water saving rice irrigation” OR (“water saving irrigation” AND “rice”) OR (“water saving irrigation practices” AND “rice”) OR (“water saving technologies” AND “rice”) OR (“water saving practices” AND “rice”) OR (“water saving methods” AND “rice”) OR “water saving rice technologies” OR “water saving rice irrigation” OR “water-saving rice” OR “water saving rice” OR “alternative rice irrigation” OR “cover crops” OR “continuous cover” OR “cover cropping” OR “permanent soil cover” OR “intercropping, rotational grazing” OR “holistic grazing” OR “multi-paddock grazing”

[Sample: Regenerative agriculture Run 2]
“regenerative agriculture” OR “conservation agriculture” OR “sustainable agriculture” OR “organic agriculture” OR “sustainable farming” OR “organic farming” OR “reduced-till” OR “reduced till” OR “minimum till” OR “minimum-till” OR “no till” OR “no-till” OR “conservation tillage” OR “fertilizer management” OR “optimal fertilizer use” OR “integrated soil fertility management” OR “4R nutrient management” OR “soil fertility management” OR “sustainable intensification” OR (“alternate wetting and drying” AND “rice”) OR (“mid-season drainage” AND “rice”) OR “cover crops” OR “continuous cover” OR “cover cropping” OR “permanent soil cover” OR “intercropping, rotational grazing” OR “holistic grazing” OR “multi-paddock grazing”

AND

[Constraint synonym]
“feasibility” OR “constrain*” OR “limitation*” OR “obstacle*” OR “barrier*” OR “hurdle*” OR “cost*” OR “restriction*” OR “impediment*”

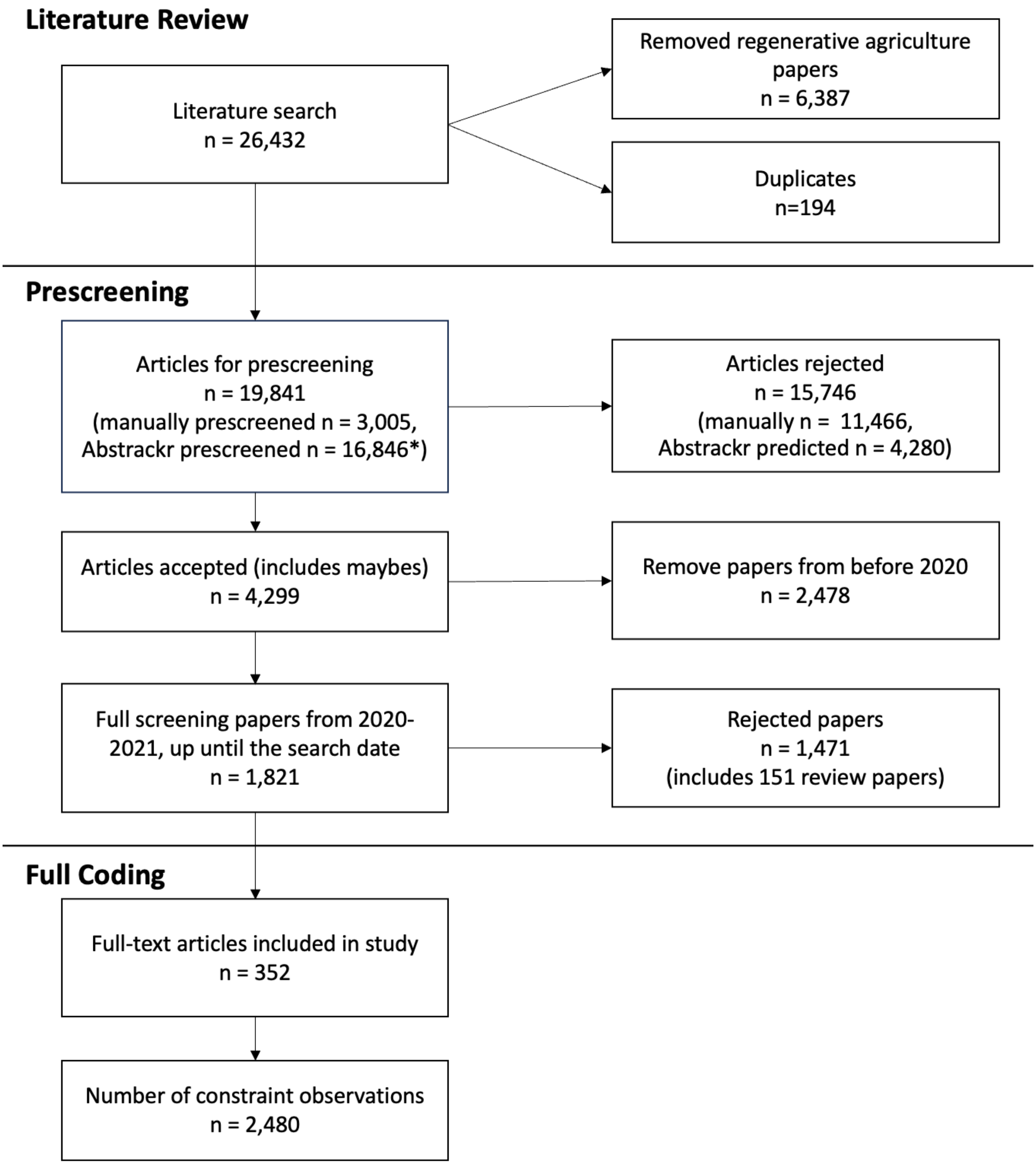

Figure S1. Flow diagram detailing steps taken for literature review. *After performing a code-check before submission, we found that the total number of articles uploaded for review in Abstrackr (n=16,846) likely contained additional duplicate articles, as updating our code reduced the number of de-duplicated and relevant entries by 28 observations (n=16,819). When we checked screened articles exported from Abstrackr, we found no duplicates, and thus our search and screening process was likely unaffected by the encoding error.

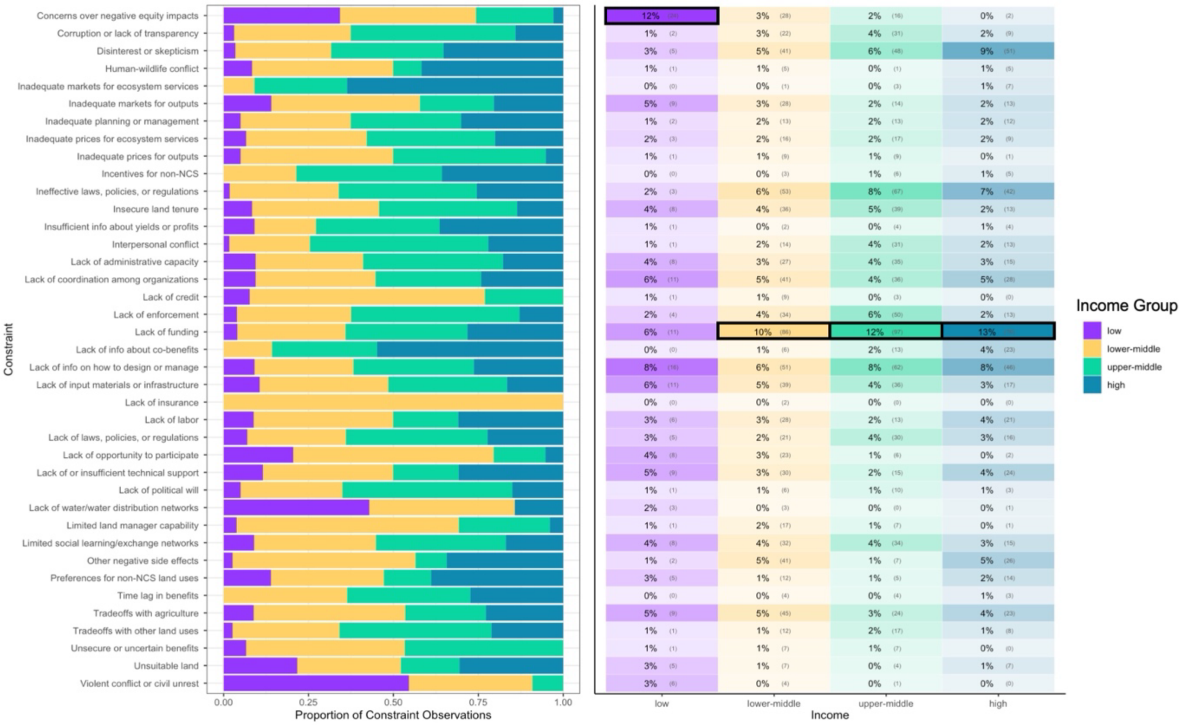

Figure S2. Constraints analyzed by World Bank income groups. A: Breakdown of constraints by income group: Percent breakdown of each constraint by income groups (e.g., 40% of observations of “Concerns over negative equity impacts” were found in lower-middle income countries. **B: Breakdown of income groups by constraint:** Frequency of each constraint category found for each income group. Shading represents the percent breakdown of each income group by constraint. Black box indicates the most frequent constraint in each income group. For example, “Lack of funding” was observed 76 times in high income countries, representing 13% of constraints in high income countries, the most frequent constraint for this income group.

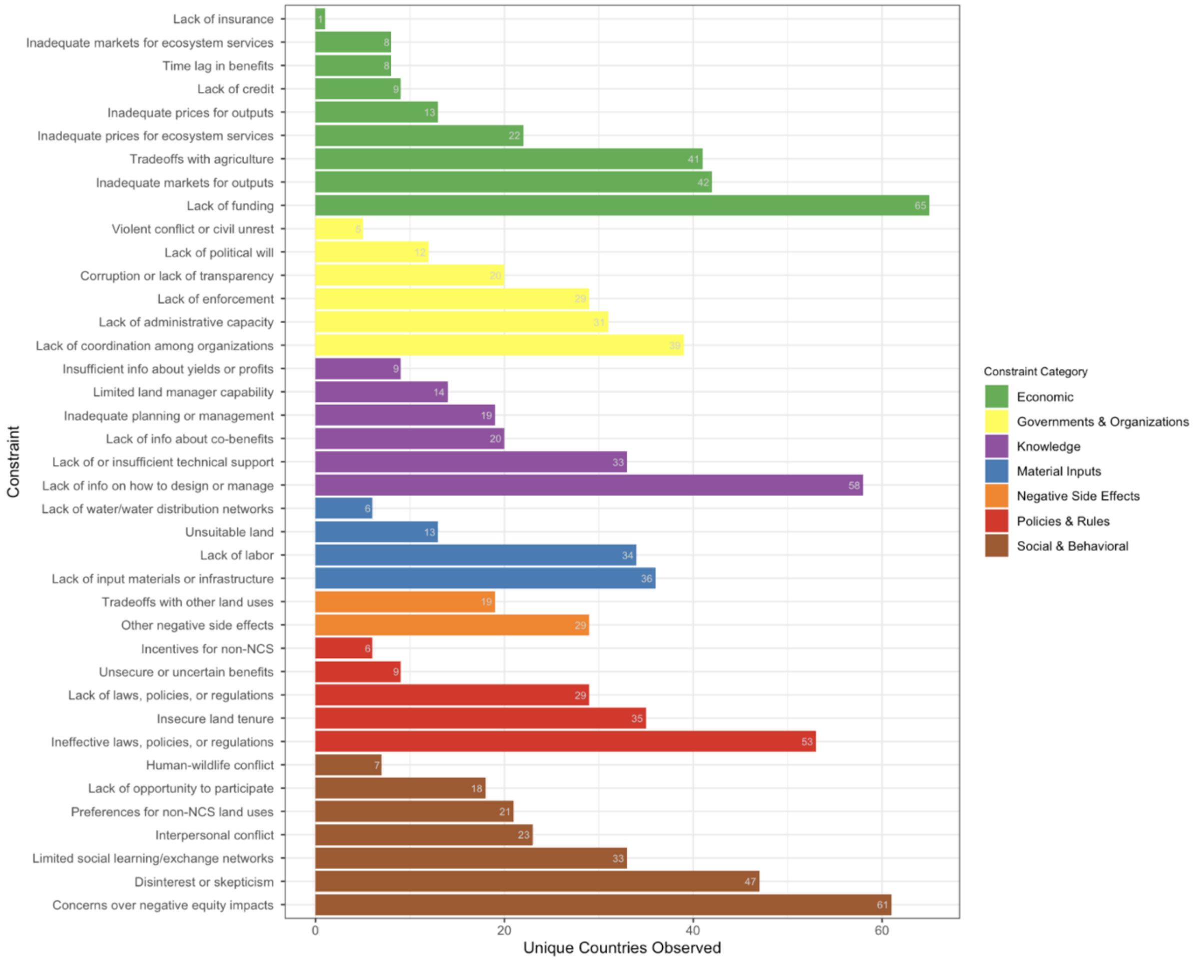

Figure S3: Number of unique countries for which each constraint was observed.

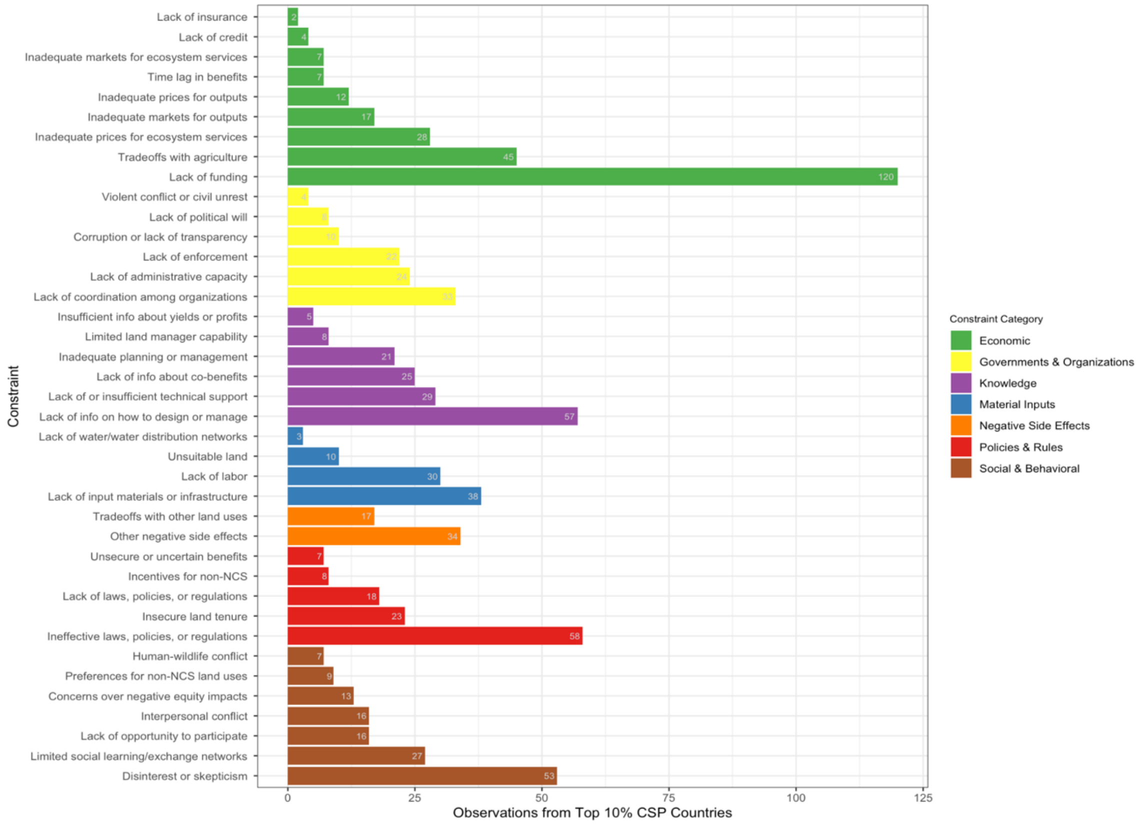

Figure S4. Constraints from the 10% of countries with the highest biophysical NCS CMP (21 countries), comprising 68% of the world's NCS CMP.

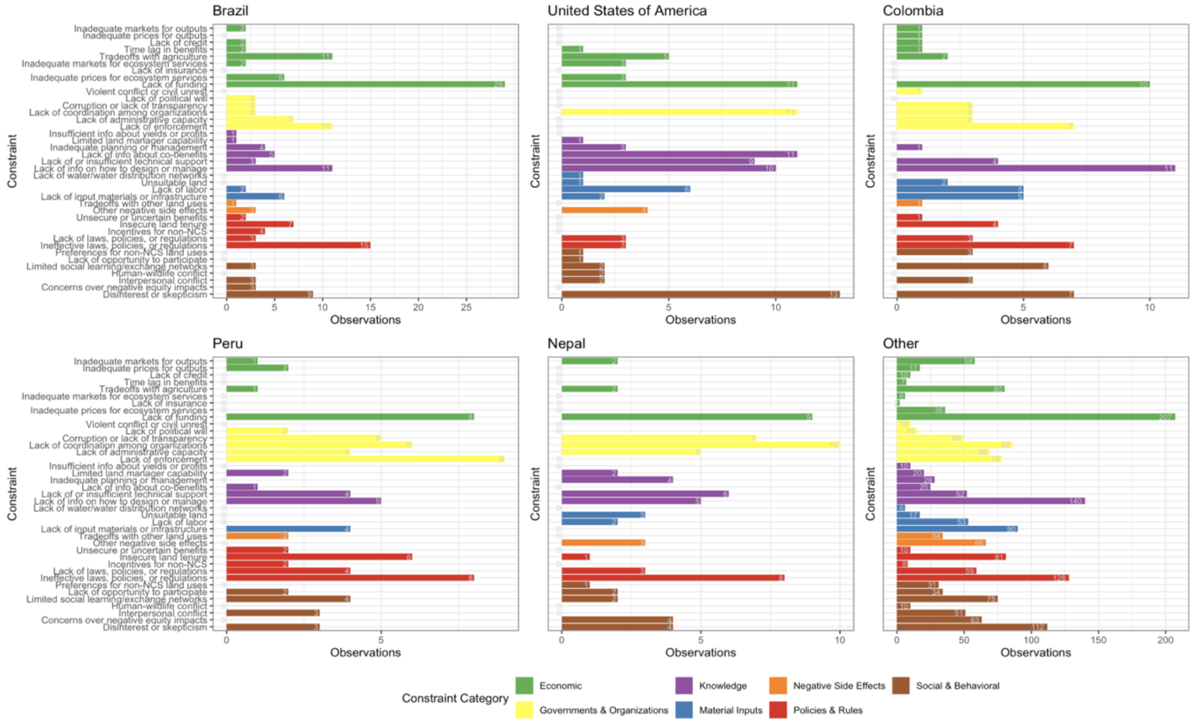

Figure S5: Number of unique observations for each constraint for the five countries with the most observations (Brazil, United States of America, Colombia, Peru, and Nepal) and for all countries except these five.

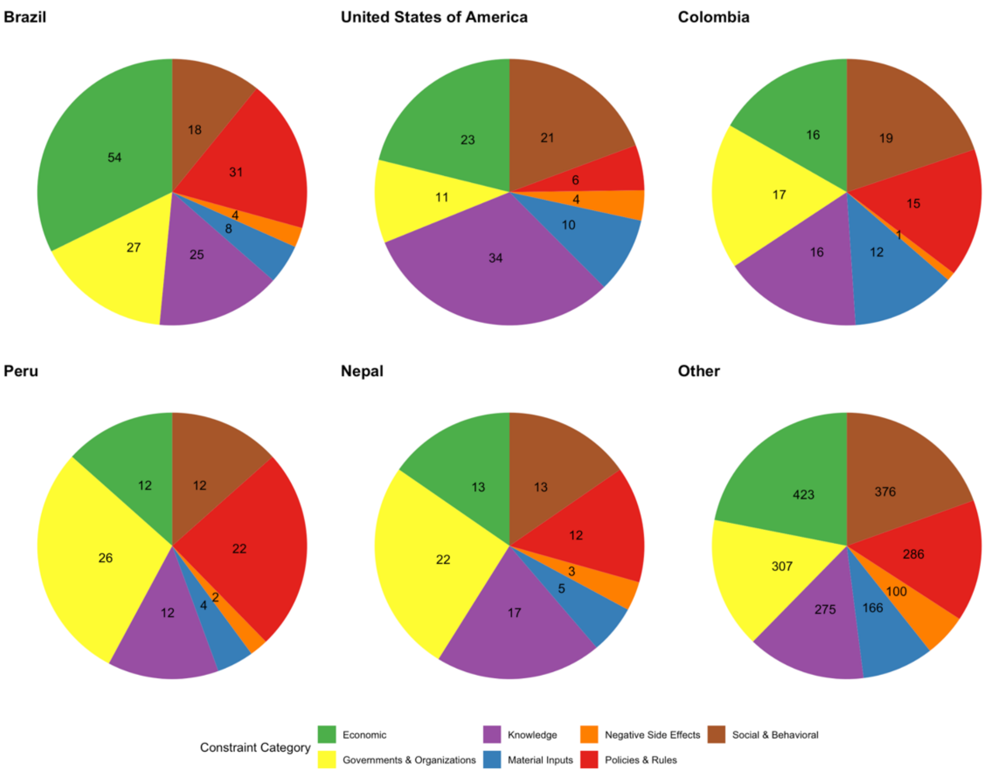

Figure S6: Number of unique observations for each constraint category for the five countries with the most observations (Brazil, United States of America, Colombia, Peru, and Nepal) and for all other countries combined.

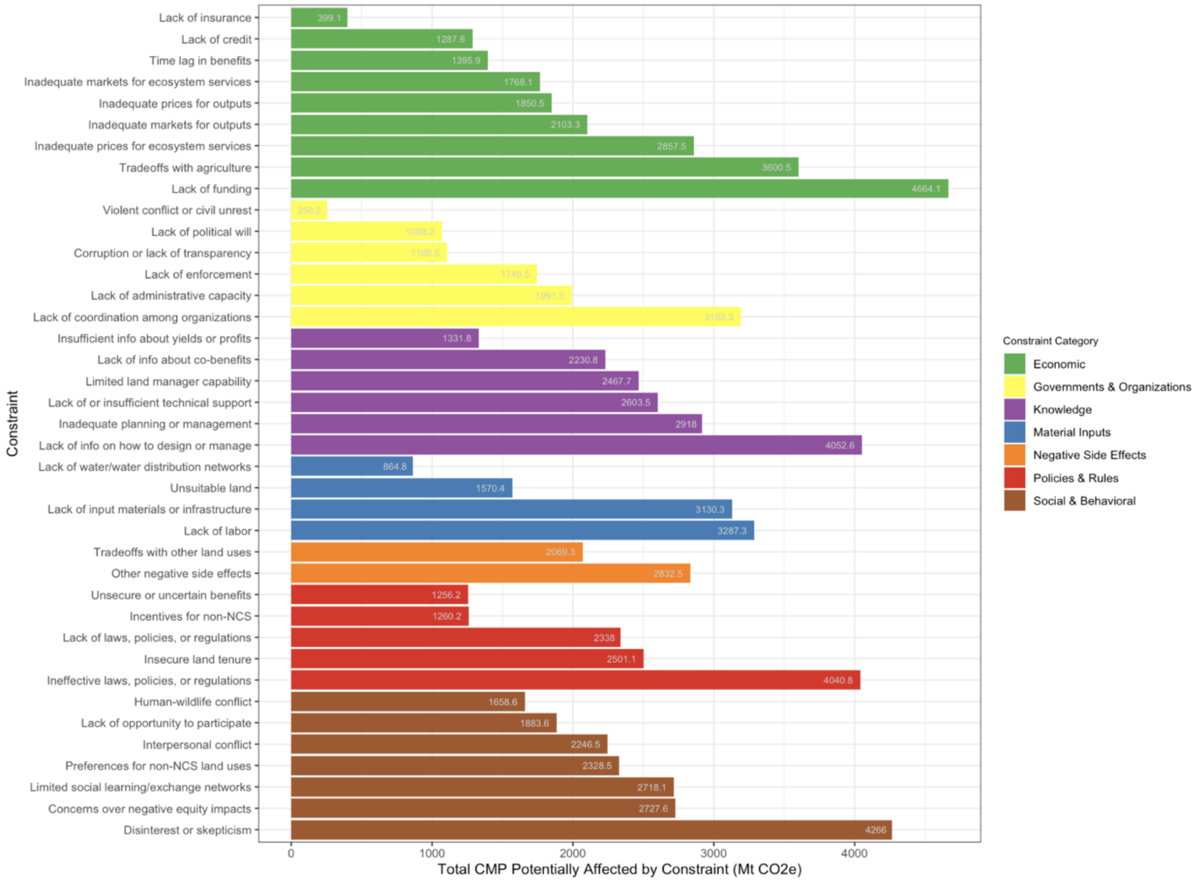

Figure S7: Total Climate Mitigation Potential (CMP) potentially affected by each constraint (Mt CO_2e). Calculated by adding up the total CMP for each country for which the constraint was observed (10). See SI text for caveats and limitations of these estimates.

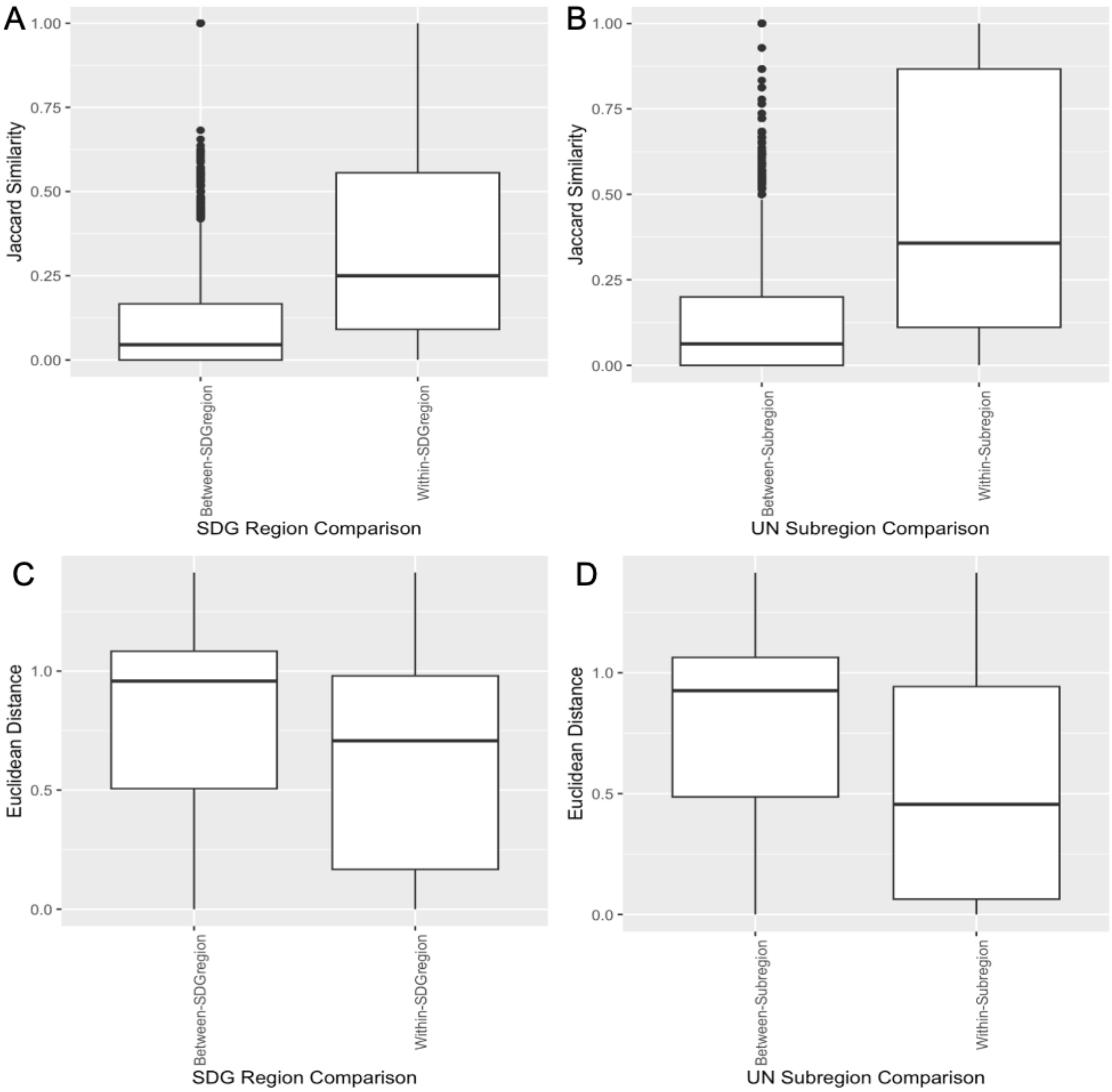

Figure S8. Comparison of constraints faced by counties within the same SDG region (A and C) and within the same UN subregion (B and D). Jaccard similarity (panels A and B) shows similarity of constraint presence in each country, with higher similarity indicating more similar constraints present among countries. Euclidean distance (panels C and D) shows dissimilarity in each country's constraint proportions (the portion of a country's total constraint observations composed by each constraint), with higher distances indicating less similar constraint ratios between countries.

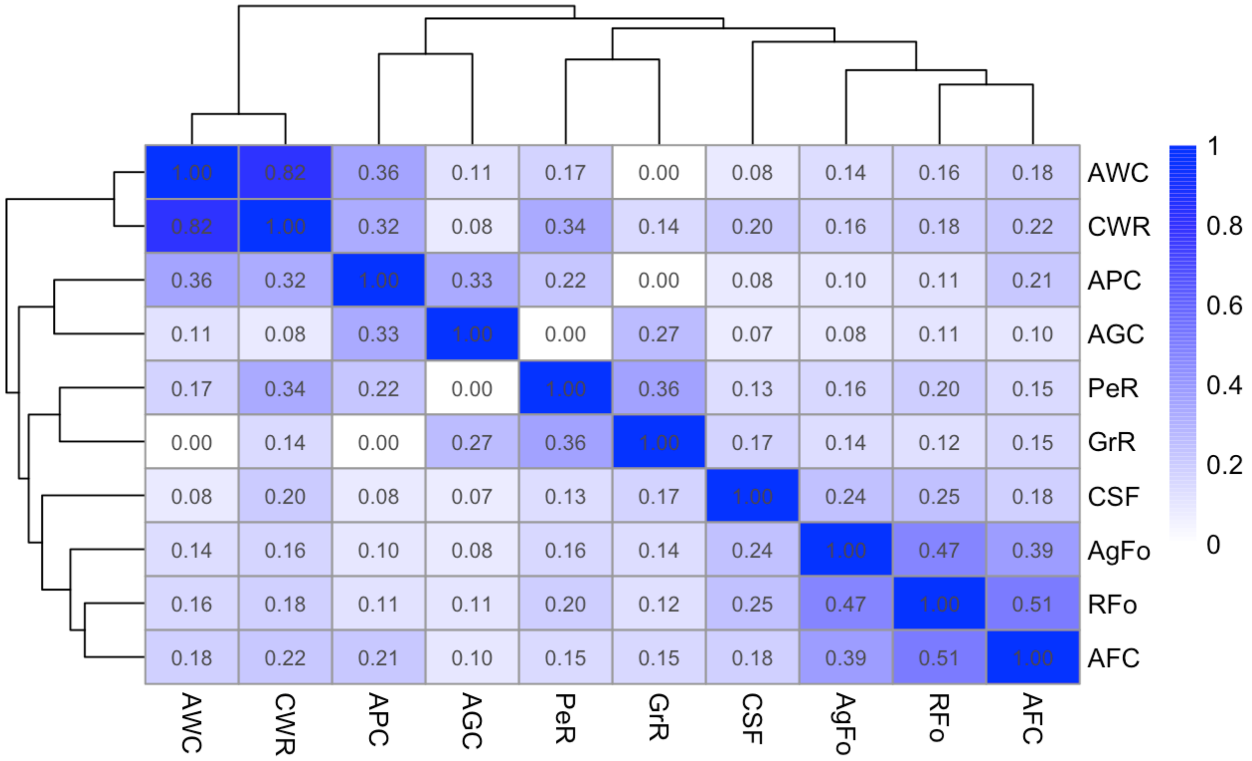

Figure S9. Similarity in constraints observed between pairs of disaggregated pathways within a UN subregion (Jaccard similarity), averaged across all UN subregions. Higher values indicate that within UN subregions, the two pathways observed similar constraints on average. For instance, UN subregions had an average Jaccard similarity between reforestation (RFo) and avoided forest conversion (AFC) of 51%.

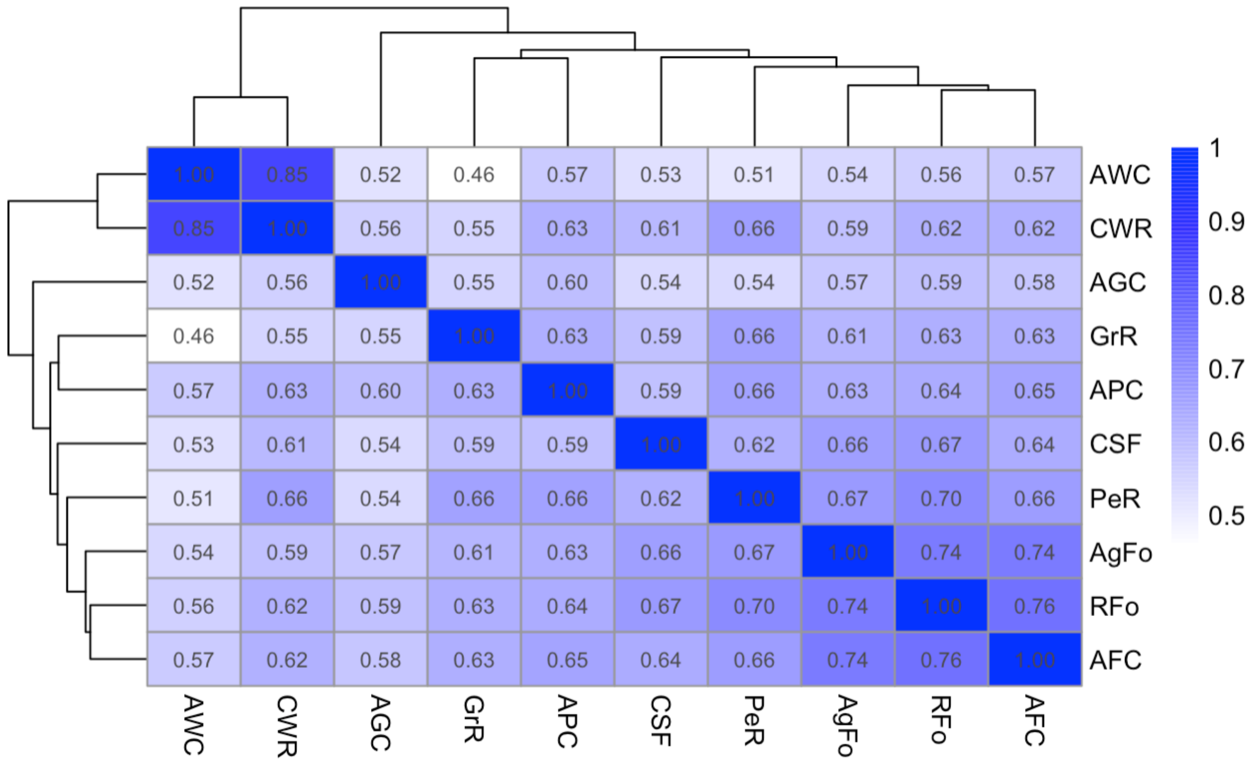

Figure S10. Similarity in constraint shares between pairs of disaggregated pathways within a UN subregion (Euclidean similarity), averaged across all UN subregions. Higher values indicate that within UN subregions, the two pathways had similar portions of each constraint on average.

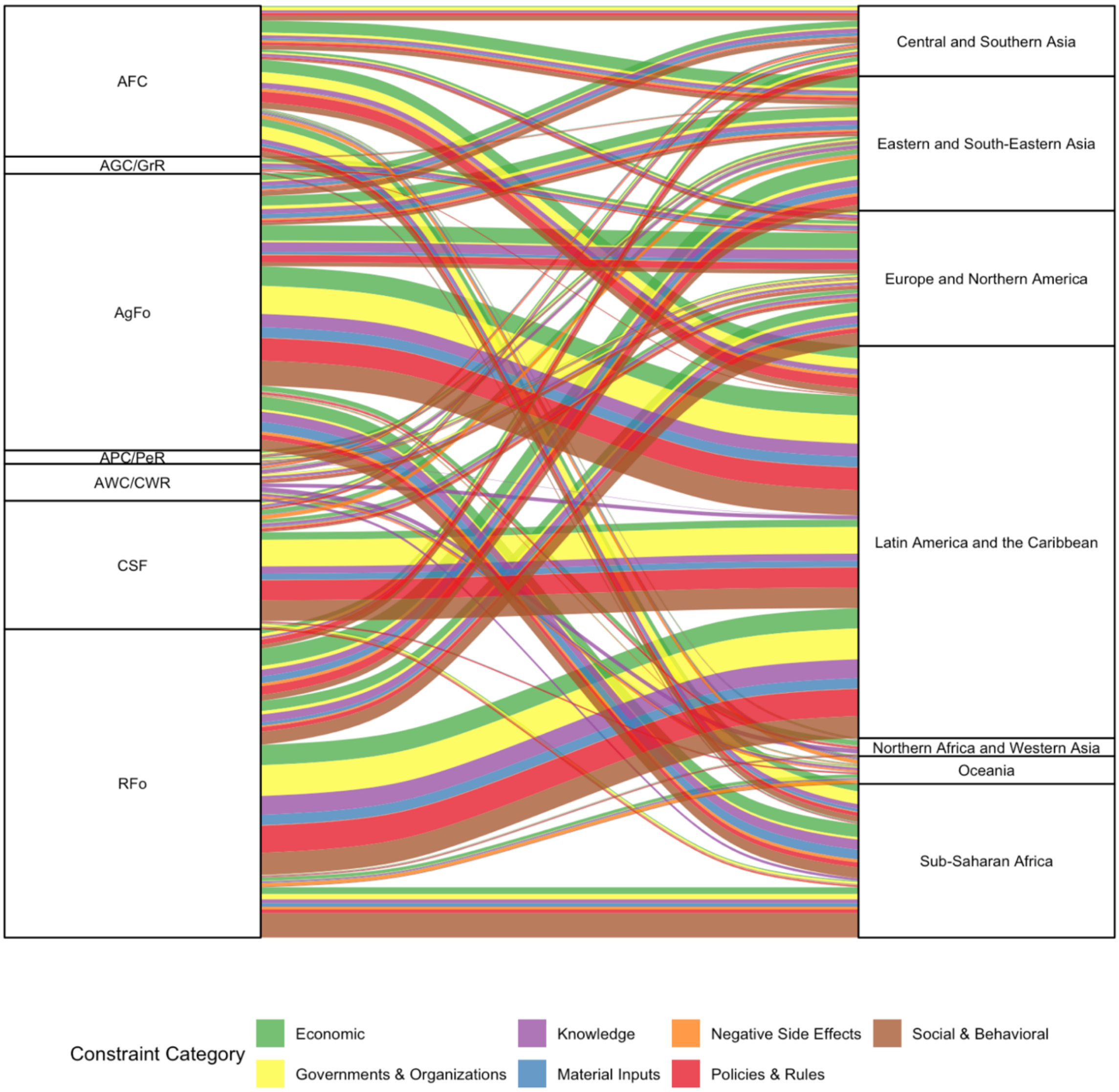

Figure S11. Fluvial plot showing relationship between constraints for each pathway (left column) in each SDG region (right column), colored by constraint category.

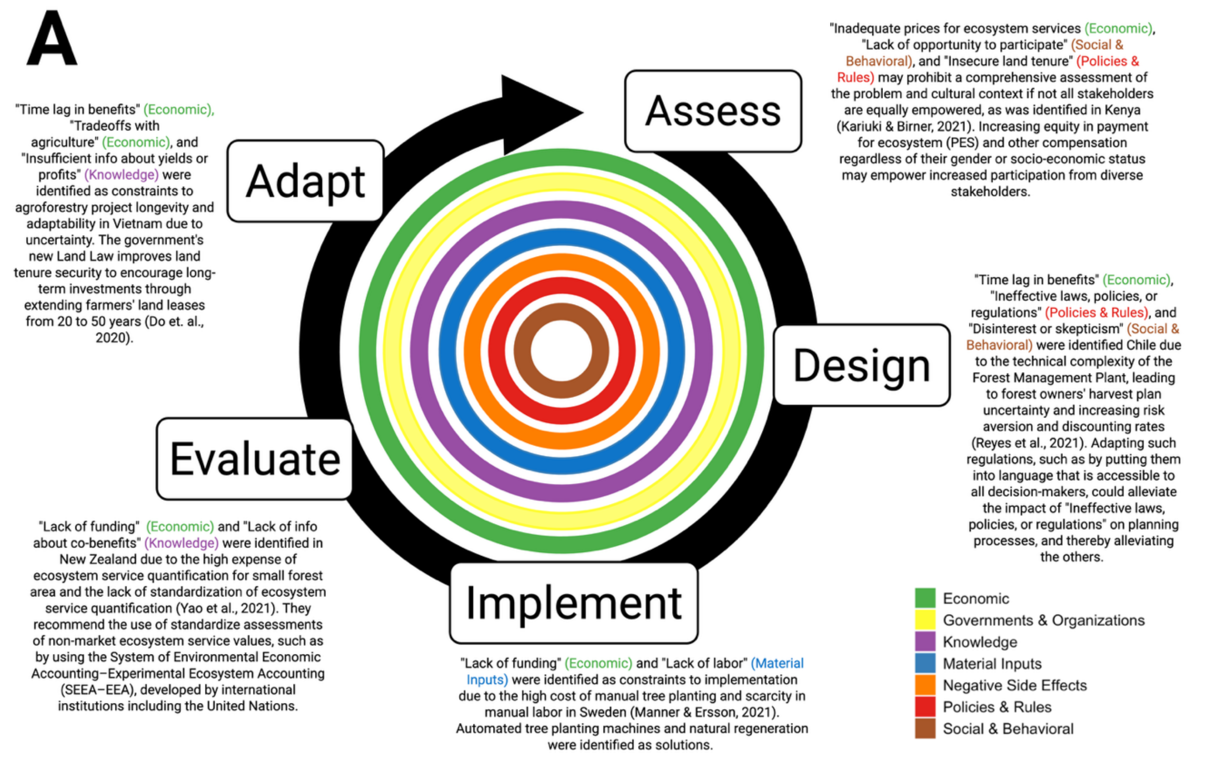

B

	Assess	Design	Implement	Evaluate	Adapt
**Example guiding questions to identify constraints at each phase**	• Economic: What financial limitations could hinder NCS implementation?<br>• Knowledge: What technical knowledge gaps exist among stakeholders, and how do these impact implementation?<br>• Social & Behavioral: Are there cultural norms, power dynamics, or lack of trust that could limit participation?<br>• Policies & Rules: Are land tenure rights and benefit-sharing mechanisms clearly defined?<br>• Governments & Organizations: Do governance weaknesses exist?<br>• Material Inputs: Are there infrastructure gaps that could prevent success?<br>• Negative Side Effects: What competing land-use pressures might create conflicts?	• Economic: What financial constraints must be accounted for in planning?<br>• Knowledge: Are there research gaps or missing extension services that would limit technical capacity?<br>• Social & Behavioral: Do social conflicts or lack of participatory decision-making structures exist?<br>• Policies & Rules: Are there conflicting policies or regulatory barriers that must be addressed?<br>• Governments & Organizations: Do relevant institutions have the capacity to oversee implementation?<br>• Material Inputs: Are there constraints in resource availability?<br>• Negative Side Effects: Are there economic or ecological trade-offs between NCS and alternative land uses?	• Economic:Are there funding bottlenecks or delays in financial disbursement?<br>• Knowledge: Is there a lack of technical support for landowners or implementers?<br>• Social & Behavioral: Are key stakeholders resisting implementation due to skepticism or competing interests?<br>• Policies & Rules: Do regulatory or policy gaps slow down implementation?<br>• Governments & Organizations: Are implementing institutions coordinated and capable?<br>• Material Inputs: Are key materials available?<br>• Negative Side Effects: Are there unintended socio-economic consequences emerging from implementation?	• Economic: Are there financial constraints limiting long-term monitoring?<br>• Knowledge: Are monitoring technologies or expertise insufficient?<br>• Social & Behavioral: Has implementation changed community attitudes or created equity issues?<br>• Policies & Rules: Are data collection methods aligned with national or global reporting requirements?<br>• Governments & Organizations: Is there institutional capacity to enforce and maintain monitoring systems?<br>• Material Inputs: Is there sufficient labor to conduct monitoring?<br>• Negative Side Effects: Have unintended negative effects emerged?	• Economic: What financial adjustments are needed to improve future NCS projects?<br>• Knowledge: What lessons can be incorporated into adaptive management?<br>• Social & Behavioral: How can participatory processes be improved to address new challenges?<br>• Policies & Rules: What regulatory changes are needed to support adaptive management?<br>• Governments & Organizations: How can institutions be restructured to support flexible decision-making?<br>• Material Inputs: What new investments are needed to remove persistent material constraints?<br>• Negative Side Effects: How can emerging trade-offs be mitigated to improve long-term effectiveness?
**Example guiding questions to identify solutions to co-occurring constraints**	• What governance reforms or collaborative models could improve institutional coordination? (*Governments & Organizations, Policies & Rules, Economic*)<br>• How can local and scientific knowledge be integrated to inform planning? (*Knowledge, Social & Behavioral, Governments & Organizations*)<br>• How can supply chains and infrastructure be improved to ensure the availability of key inputs? (*Material Inputs, Economic, Governments & Organizations*)	• How can project designs reduce costs while maintaining ecological effectiveness? (*Economic, Material Inputs*)<br>• How can participatory planning approaches improve stakeholder buy-in and social equity? (*Social & Behavioral, Policies & Rules, Governments & Organizations*)<br>• How can behavioral change interventions increase adoption? (*Social & Behavioral, Policies & Rules, Knowledge*)	• What training and technical support programs can be introduced to build local implementation capacity? (*Knowledge, Social & Behavioral, Governments & Organizations*)<br>• How can financing structures prevent funding gaps? (*Economic, Policies & Rules*)<br>• What infrastructure investments are necessary to remove material constraints? (*Material Inputs, Economic, Governments & Organizations*)	• What sustainable finance mechanisms can support long-term monitoring? (*Economic, Policies & Rules, Governments & Organizations*)<br>• What cost-effective monitoring technologies can improve efficiency? (*Knowledge, Material Inputs, Economic*)<br>• How can participatory monitoring programs increase engagement and data reliability? (*Social & Behavioral, Knowledge, Policies & Rules*)<br>• How can evaluation frameworks measure long-term economic, ecological, and social success? (*Knowledge, Economic, Policies & Rules, Negative Side Effects*)	• What flexible financial instruments can improve project adaptability? (*Economic, Policies & Rules, Governments & Organizations*)<br>• How can negative trade-offs be mitigated through policy adjustments or additional incentives? (*Negative Side Effects, Social & Behavioral, Economic*)<br>• How can governance systems institutionalize adaptive management? (*Governments & Organizations, Policies & Rules, Economic*)<br>• What mechanisms can ensure that lessons learned are applied to future projects? (*Knowledge, Policies & Rules, Governments & Organizations*)

Figure S12. Framework to identify constraints and solutions at different phases of NCS implementation. A) Adaptive management cycle adapted to NCS implementation. Inner colored circles illustrate that constraints from each category may occur at any phase. Text outside the circle diagram indicates examples from our literature review database of multiple co-occurring constraints in that phase and potential solutions (11–15). B) Example guiding questions to identify constraints at each phase from each category, and example guiding questions to identify integrated solutions to constraints that may co-occur in each phase (16).

Table S1: Descriptions of the constraints included in the analysis to provide context for the type of information that constituted a particular constraint.

Constraint	Abbreviated Name	Description
	**Economic Constraints**	
**High implementation cost or lack of funding**	Lack of funding	Lack of access to funding for establishing, implementing, or maintaining the NCS pathway, or prohibitively high costs for establishing, implementing, or maintaining the NCS pathway.
**Lack of access to credit**	Lack of credit	Lack of access to financial credit for establishing, implementing, or maintaining the NCS pathway. Observations were only included in this category if they specified credit.
**Lack of insurance for NCS assets or outputs**	Lack of insurance	Lack of access to insurance for risk management related to establishing, implementing or maintaining the NCS.
**Inadequate/inaccessible markets for ecosystem services or biodiversity**	Inadequate markets for ecosystem services	Inadequate and/or inaccessible economic markets for ecosystem services or biodiversity provided by the NCS pathway. Ecosystem services are defined here as the benefits people obtain from ecosystems (IPBES), including tangible (e.g., carbon sequestration) and intangible (e.g., cultural value) benefits. While food products may be considered ecosystem services, markets for outputs such a food are included in the 'markets for outputs' constraint.
**Inadequate/inaccessible markets for outputs**	Inadequate markets for outputs	Inadequate and/or inaccessible economic markets for outputs of the NCS pathway, such as food and forest products. While such outputs can be considered ecosystem services, they are included here to differentiate direct market outputs from services which have typically been excluded from markets.
**Inadequate prices for ecosystem services or biodiversity conservation**	Inadequate prices for ecosystem services	Inadequate prices for ecosystem services, such as carbon or water yield, including payment for ecosystem service transactions in which the price is too low to incentivize or maintain the NCS pathway effectively.
**Inadequate prices for outputs**	Inadequate prices for outputs	Inadequate prices for NCS outputs such as food or forest products, in which the price is too low to incentivize or maintain the NCS pathway effectively.
**Time lag in benefits**	Time lag in benefits	A time lag between implementation of the NCS and the accrual of benefits from the NCS which negatively impacts interest and/or ability to establish, implement, or maintain the NCS pathway.
**Tradeoffs with agricultural yield or profit**	Tradeoffs with agriculture	Tradeoffs between the NCS pathway and yield and/or profit of non-NCS agricultural products (i.e., row crop or livestock) which negatively impacts interest and/or ability to establish, implement, or maintain the NCS pathway.
	**Knowledge Constraints**	
**Insufficient information about yields, profits, or prices**	Insufficient info about yields or profits	Insufficient information about financial outcomes and marketable products resulting from the NCS, such as agricultural yields and forest product prices.

		This constraint includes lack of information, lack of consensus on information, or lack of methodologies for acquiring information.
**Lack of information about on-site benefits or co-benefits**	Lack of info about co-benefits	Lack of information about the on-site benefits or co-benefits resulting from the NCS pathway, such as short- and long-term mitigation potential, conservation/restoration outcomes, and wider societal welfare outcomes. This constraint includes lack of information, lack of consensus on information, or lack of methodologies for acquiring information (ex. soil fertility, fodder or shade for livestock, etc.).
**Lack of information on how to design, begin, or manage the NCS**	Lack of info on how to design or manage	Lack of information about how to design, begin, or manage the NCS pathway, such as limited information on implementation options, monitoring and data limitations, or ecological knowledge gaps. This constraint includes lack of information, lack of consensus on information, or lack of methodologies for acquiring information.
**Lack of or insufficient technical advice or technical support for land managers/operators**	Lack of or insufficient technical support	Lack of or insufficient technical support for implementing the NCS, including inadequate or inaccessible extension services.
**Limited land manager/operator literacy, numeracy, or technological capabilities**	Limited land manager capability	Limited land manager or operator capabilities, including literacy, numeracy, or technological ability.
**Inadequate planning or management**	Inadequate planning or management	Insufficient or poor planning or management of NCS. Information to manage NCS exists but planning or management was not executed effectively.
	**Social & Behavioral Constraints**	
**Preferences or norms favoring non-NCS land uses**	Preferences for non-NCS land uses	Preferences for land use(s) other than the focal NCS pathway, including social norms favoring other land uses, which negatively impacts the establishment, implementation, or maintenance of the NCS pathway.
**Disinterest or skepticism of NCS or lack of trust toward NCS promoters**	Disinterest or skepticism	Disinterest and/or skepticism in the NCS pathway, including skepticism toward NCS promoters or implementing bodies. This constraint includes perceptions of negative impacts that disincentivize uptake of the NCS pathway.
**Limited social learning or exchange networks**	Limited social learning/exchange networks	Limited social learning or exchange networks between individuals or groups which negatively impacts the establishment, implementation, or maintenance of the NCS pathway.
**Lack of opportunity to participate in or influence implementation due to gender, race, ethnicity, or other dimensions of identity**	Lack of opportunity to participate	Lack of opportunity to participate in or influence the implementation of NCS due to gender, race, ethnicity, or other dimensions of identity which negatively impacts the establishment, implementation, or maintenance of the NCS pathway.
**Interpersonal conflict**	Interpersonal conflict	Interpersonal conflict between individuals or groups which negatively impacts the establishment, implementation, or maintenance of the NCS pathway.

**Human-wildlife conflict**	Human-wildlife conflict	Human-wildlife conflict which negatively impacts the establishment, implementation, or maintenance of the NCS pathway. Includes human-wildlife conflict which arises from the NCS.
**Concerns over negative equity impacts**	Concerns over negative equity impacts	Concerns over the realized or potential negative equity impacts (e.g., related to gender, race, ethnicity, or other dimensions of identity) caused by the NCS, which negatively impacts the establishment, implementation, or maintenance of the NCS pathway.
**Policies & Rules Constraints**		
**Ineffective laws, policies, or regulations**	Ineffective laws, policies, or regulations	Laws, policies, regulations, or government programs, which are ineffective in establishing, implementing, maintaining, or regulating the NCS pathway due to a range of deficiencies including: regulatory barriers, ambiguity, overly prohibitive, inefficient or inconsistent processes, conflicting language, oversimplification etc.
**Lack of laws, policies, or regulations**	Lack of laws, policies, or regulations	Lack of laws, policies, regulations, or government programs which would aid in establishing, implementing, or maintaining the NCS pathway. This constraint addresses the absence of a law, policy, or regulation, in contrast to the 'ineffective laws, policies or regulations' constraint which addresses existing laws, policies, or regulations.
**Insecure land tenure**	Insecure land tenure	Lack of secure land tenure which negatively impacts the implementation and/or effectiveness of the NCS pathway, including tenure systems which are inequitable, overly complex, lack clarity, or are fragile. Includes lack of secure land management or rights to manage or sell property.
**Unsecure or uncertain NCS benefit sharing**	Unsecure or uncertain benefits	Unsecure or uncertain benefits sharing from the NCS pathway, including inequal access to benefits, unclear distribution of benefits, distance of potential beneficiaries to the NCS location, or lack of sufficient benefits. This includes insecure, uncertain, or lack of rights to use resources generated from NCS.
**Incentives for non-NCS land use**	Incentives for non-NCS	Incentives provided for non-NCS land uses (e.g., agricultural subsidies, reduced taxes, or access to credit or insurance for non-NCS) which constrain the implementation of NCS.
**Governments & Organizations Constraints**		
**Lack of administrative capacity for implementation**	Lack of administrative capacity	Lack of government or organization administrative capacity for planning or implementing the NCS pathway. If more specific information was provided about the cause of the administrative capacity issue (e.g., labor shortages leading to lack of capacity) the constraint was placed in the more detailed category.
**Lack of enforcement of laws, policies, or regulations**	Lack of enforcement	Lack of enforcement of laws, policies, regulations, or government programs, negatively effecting or preventing implementation of the NCS pathway. This

		constraint includes lack of enforcement, weak enforcement, and presence of illegal activities (e.g., illegal logging) which implies a lack of enforcement ability.
**Lack of coordination among organizations**	Lack of coordination among organizations	Lack of coordination between different organizations, sectors or administrative units within the same organization, which negatively impacts or prevents the implementation of NCS. This constraint includes lack of coordination between different scales of governance (e.g., local and national) as well as different sectors or administrative units (e.g., political silos). For lack of social coordination or communication, see constraint on limited social learning.
**Lack of political will**	Lack of political will	Lack of political will which negatively impacts the establishment, implementation, or maintenance of the NCS pathway. This constraint includes political prioritization of other goals and lack of government interest for NCS including a lack of urgency for action.
**Violent conflict or civil unrest**	Violent conflict or civil unrest	Violent conflict or civil unrest which negatively impacts the establishment, implementation, or maintenance of the NCS pathway.
**Corruption or lack of transparency**	Corruption or lack of transparency	Presence of corrupt of untransparent governance processes which negatively impact the implementation and/or effectiveness of the NCS pathway.
**Material Inputs Constraints**		
**Lack of input materials or infrastructure**	Lack of input materials or infrastructure	Lack of input materials or physical infrastructure needed to implement the NCS pathway, such as lack of seeds and seedling supply, road infrastructure, or equipment including vehicles.
**Lack of labor**	Lack of labor	Lack of labor needed to implement the NCS pathway, including labor shortages and lack of skilled labor (external or own).
**Lack of water or water distribution networks**	Lack of water/water distribution networks	Lack of water or water distribution networks for NCS pathway (e.g. irrigation)
**Unsuitable land**	Unsuitable land	Unsuitable land for the NCS pathway, including land that is too small, terrain too difficult, or not easily accessible to potential NCS land managers or operators
**Negative Side Effects**		
**Tradeoffs with other non-agriculture land uses**	Tradeoffs with other land uses	Tradeoffs between the NCS pathway and other land uses which negatively impacts interest and/or ability to establish, implement, or maintain the NCS pathway. Excludes agricultural land uses.
**Other negative side effects**	Other negative side effects	The implementation of the NCS pathway leads to a negative side effect on the pursual of other priorities (e.g., health, invasive species management, fire risk, infrastructure, etc.).

Table S2: Comparison of constraint categories and constraints used in this paper with those used in recent other studies (2–5). Numbers in parentheses indicate which constraints of ours to which other classifications are identical or similar.

**This paper**	**IPCC AR6 (2021)**	**Schulte et al. (2021)**	**Roe et al. (2021)**	**Karki et al. (2023)**
NCS implementation constraint categories and constraints	*AFOLU barriers and opportunities*	*Categories of NCS enabling factors*	*NCS feasibility dimensions and indicators*	*Barriers and sub-barriers to LMT deployment*
**Economic**	**Socio-economic**	**Economic**	**Economic**	**Economic**
• High implementation costs or lack of funding (1)<br>• Lack of access to credit (2)<br>• Lack of insurance for NCS assets or outputs (3)<br>• Inadequate/inaccessible markets for ecosystem services or biodiversity (4)<br>• Inadequate/ inaccessible markets for NCS outputs (5)<br>• Inadequate prices for ecosystem services or biodiversity conservation (6)<br>• Inadequate prices for outputs (7)<br>• Time lag in benefits (8)<br>• Tradeoffs with agricultural yield or profit (9)	• Design and coverage of financing mechanisms (1)<br>• Scale and accessibility of financing (1,2,6)<br>• Risk aversion and uncertainty coupled with significant upfront investments and time lags (1,6,10,11,8)<br>• Impacts on poverty and food security (9)<br>• Cultural values and social acceptance (16)	• Delivery of benefits<br>• Accessibility (1)<br>• Labor availability (projects, programs, or gov. ministries) (28,35)<br>• Market competitiveness (1,4,5,6,7)<br>**Financial**<br>• Performance-based finance (2,4)<br>• Secure funding (1)<br>• Donor finance (1)<br>• Financial services (2)	• GDP per capita (PPP)<br>• Forest rents ($/ha)<br>• Agricultural value added ($/ha)<br>• Ease of doing business<br>• Ease of obtaining a bank loan (2)	• *Costs* (Unable to afford specialized machinery; large initial investment, expensive to deploy at a scale where there is large potential) (1)<br>• *Income* (potential for income decline due to tradeoffs; transitional period with higher production costs and lower income; lack of incentives) (8,9)<br>• *Value* (difficulty of monetizing non-market benefits/ES) (4,6)
**Knowledge**	**Technological**	**Technical**	**Technological**	**Technological**
• Insufficient information about yields, profits, or prices (10)<br>• Lack of information about on-site benefits or co-benefits (11)<br>• Lack of information on how to design, begin, or manage the NCS (12)<br>• Lack of or insufficient technical advice or technical	• Monitoring, reporting, and verification needs (1,11 – specifically, on-site C sequestration)	• Technical assistance (11)<br>• Monitoring, reporting, verification (11 – specifically, on-site C sequestration)<br>• Land-use planning (12)<br>• Compatible practices (12)	• Access to information and communications (10,11,12,13)<br>• Market access and infrastructure (4,5)<br>• Agricultural total factor productivity	• *Complexity* (Difficult to adopt due to the complexity of LMTs for farmers/land managers; Requiring high management skills) (12,14,15)<br>• *Resources* (Difficulty in access to specialized machinery; Unavailability of transportation infrastructure; Lack of access to inputs; Limited/no access to credit; Limited extension facilities) (4,13,34,36)<br>• *Development* (Lack of development of efficient monitoring, reporting and verification; Large

support for land managers/operators (13)<br>• Limited land manager/operator literacy, numeracy or technological capabilities (14)<br>• Inadequate planning or management (15)		• Research (10,11,12)		uncertainties about the benefits of an LMT; Limited scientific understanding of land suitability; Technological readiness) (10,11,12,14)
**Social & Behavioral**		**Social**	**Socio-cultural**	**Socio-cultural**
• Preferences or norms favoring non-NCS land uses (16)<br>• Disinterest or skepticism of NCS or lack of trust toward NCS promoters (17)<br>• Limited social learning or exchange networks (18)<br>• Lack of opportunity to participate in or influence the implementation due to gender, race, ethnicity, or other dimensions of identity (19)<br>• Interpersonal conflict (20)<br>• Human-wildlife conflict (21)<br>• Concerns over negative equity impacts (22)		• IPLC engagement (*19*)<br>• Acceptance (local) (16,17)<br>• Stakeholder consultation<br>• Recognition of traditional values<br>• Free, prior, informed consent (FPIC)	• Personal rights (political rights, freedom of expression, freedom of religion, access to justice, property rights for women)<br>• Nutrition and basic medical care	• *Norms and values* (traditional beliefs, cultural affiliation to a traditional production system, cultural norm) (16)<br>• *Knowledge and perception* (Lack of knowledge, limited awareness of value and benefits, low social acceptance, social pressure against LMTs, perceived threats from LMTs) (10,11,17,21)<br>• *Behavior* (Difficulty in long-term decision for transition, lack of trust, habit of relying on conventional practice) (17)
				**Ethical**
				• *Conflicts* (risks of land grabbing; Issue of equitable benefit sharing; Issue of social conflicts) (20,22,25,26)<br>• *Tradeoffs* (Land availability and competition with other land uses; Possible increase in food prices and compromise food security; Negative effect on the environment) (*9*,38,39)<br>• *Fairness* (Limited access of women and minority groups to resources and land; No consideration of the rights of Indigenous People and local communities) (19)
**Policies & Rules**	**Institutional**	**Institutional**	**Institutional**	**Institutional**
• Ineffective laws, policies or regulations (23)<br>• Lack of laws, policies or regulations (24)	• Transparent and accountable governance (33)	• Institutional frameworks (23,24)<br>• Land/customary tights (25)	• Voice and accountability<br>• Political stability and absence of violence (32)	• ***Policy*** **(**Lack of policy support mechanism to set explicit incentives; Lack of policy implementation; Disinterest of policymakers) (24,28,31)

• Insecure land tenure (25)<br>• Unsecure or uncertain NCS benefit sharing (26)<br>• Incentives for non-NCS land use (27)	• Clear land tenure and land-use rights (25)<br>• Institutional capacity (28,*30*)	• Realistic requirements<br>• Enforcement (29)<br>• Regulatory support (23,24)<br>• Clear carbon rights	• Government effectiveness (28,29,30)<br>• Regulatory quality (22)<br>• Rule of law (29,33)<br>• Control of corruption (33)<br>• Tenure insecurity (25)	• ***Governance*** **(**Lack of cross-sectoral responsibility-sharing; Top-down approach; Coordination between stakeholders; Lack of proper monitoring) (30)<br>• ***Regulation*** **(**Counter-productive public policies and legislation; Lack of standards and protocols to measure carbon sequestration) (22,*24*,26)
**Governments & Organizations**		**Political**		
• Lack of administrative capacity for implementation (28)<br>• Lack of enforcement of laws, policies or regulations (29)<br>• Lack of coordination among organizations (30)<br>• Lack of political will (31)<br>• Violent conflict or civil unrest (32)<br>• Corruption or lack of transparency (33)		• Collaboration/ coordination (30)<br>• Acceptance (leadership) (31)<br>• Political integrity (33)<br>• Transparency (33)<br>• Accountability		
**Material Inputs**	**Ecological**	**Biophysical**	**Geophysical**	
• Lack of input materials or infrastructure (34)<br>• Lack of labor (35)<br>• Lack of water or water distribution networks (36)<br>• Unsuitable land (37)	• Land and water availability (36,37)<br>• Specific soil conditions, water availability, GHG emission potential, natural variability and resilience (*10,11,12*)<br>• Adaptation benefits and biodiversity conservation (11)	• Land conditions (37)<br>• Climatic conditions<br>• Species diversity<br>• Carbon stock potential	• Total land-based technical mitigation potential / total land area (tCO_2/ha)	
			**Environmental-ecological**	
			• Environmental performance index (EPI)	
**Negative Side Effects**				
• Tradeoffs with other non-agriculture land uses (38)				

• Other negative side effects of NCS (39)				

Notes: Italicized numbers indicate potential or partial match with the respective constraint used in this paper. AFOLU – Agriculture, forestry and other land uses. ES – ecosystem services. GHG – greenhouse gas. LMT – land-based mitigation technology. NCS – natural climate solution.

Table S3: Example solutions identified in the literature review for each constraint.

Constraint	Example Solutions
	**Economic Constraints**
**High implementation cost or lack of funding**	• Reduce implementation costs, such as by implementing natural forest regeneration or mechanized tree planting (14, 16).<br>• Facilitate certification-based, quality-assurance price premium or additional market access to help offset costs (17).<br>• Leverage finance from multilateral institutions such as the World Bank, Asian Development Bank, Global Environment Facility and UNFCCC framework, especially in the absence of government funding (18).<br>• Create long-term, performance-based financial incentives to support reforestation and avoid reversal (19).
**Lack of access to credit**	• Public policy to facilitate funding schemes with low interest rates for agroforestry (20).<br>• Credit schemes that consider labor opportunity costs, fertilizer subsidies, land rental value, and the regional economic benefits of adoption, as was found for shade cacao agroforestry in Ghana (21).<br>• Develop incentives through partnerships between different sectors, such as the Forests National Corporation and government agencies to support forest beekeeping in Sudan (22).
**Lack of insurance for NCS assets or outputs**	NA
**Inadequate/ inaccessible markets for ecosystem services or biodiversity**	• Governments, non-governmental organizations, and private sector collaboration create structured markets for payment for ecosystem services (PES) and certified timber or nontimber products (23).<br>• Research financial performance optimization for both timber and carbon sequestration to help alleviate carbon market uncertainty concerns (24).<br>• Facilitate early monetization of REDD+ credits through tailored financial instruments, as well as calculations of the social cost of carbon (25).
**Inadequate/ inaccessible markets for outputs**	• Create stable markets through establishing links to processing factories, which buy agroforestry products such as longan fruit and make value-added products (26).<br>• Target high-end markets through increasing product quality and value-added labels (27).<br>• Develop new sources of income, such as ecotourism (28).

**Inadequate prices for ecosystem services or biodiversity conservation**	• Develop long-term payment for ecosystem services (PES) arrangements and include regular opportunities to re-negotiate payments, such as in response to costs changes and performance (29).<br>• Create biomass or carbon credits that match or exceed profits from traditional agriculture (30).<br>• Improve monitoring to reduce forest carbon market uncertainties (31).
**Inadequate prices for outputs**	• Create farmers' cooperative to improve agroforestry product quality (26).<br>• Integrate bees and livestock in to agroforestry system to increase financial benefits and food security (32).<br>• Create laws and policies to incentivize sustainable livelihood opportunities, such as forest beekeeping (22).
**Time lag in benefits**	• Improve land tenure security to encourage long-term investments, such as through policies that extend farmers' land leases (11).<br>• Reduce uncertainty around forest management decisions, such as by putting regulations into language that is accessible to all decision-makers (13).<br>• Improve technical assistance to landowners so they can make more informed decisions (33).
**Tradeoffs with agricultural yield or profit**	• Facilitate access to additional financial services to offset production decreases, improve market linkages, and ensure fair prices of sustainable agricultural products (34).<br>• Increase awareness of co-benefits to farmers, including construction materials and fences, through regional agricultural extension services (35).<br>• Research innovative practices to reduce this tradeoff, such as pasture improvement and agroforestry systems (36).
**Knowledge Constraints**	
**Insufficient information about yields, profits, or prices**	• Assess the impact of carbon revenue on the profitability of agroforestry systems compared to monocultures, especially in under-studied regions (37).<br>• Improve agroforestry cost and revenue accounting, such as through integrating total income, total capital, and economic ecosystem sustainability analysis (38).<br>• Facilitate opportunities of knowledgeable key actors to share their expertise in small group settings (39).
**Lack of information about on-site benefits or co-benefits**	• Standardize assessments of non-market ecosystem service values, such as by using the System of Environmental Economic Accounting–Experimental Ecosystem Accounting (SEEA–EEA), developed by international institutions including the United Nations (15).<br>• Create targeted extension efforts to show farmers that buffer strip and hedgerow restoration benefits both the environment and agricultural production (40).<br>• Harness new geospatial data products that have both broad geographic coverage and fine spatial resolution to monitor progress toward large-scale conservation targets (41).
**Lack of information on how to design, begin, or manage the NCS**	• Establish national-level database with baseline data and centralized information on forest landscape initiatives across the country to support scientifically-grounded ecosystem restoration planning (42).<br>• Improve knowledge transfer, such as through field schools and farmer-to-farmer exchange visits and trainings (43).<br>• Leverage insights from emerging technology, such as drone photogrammetric data, to improve management of regenerating forests (44).

	• Increase research on the cultivation of native tree species, such as through public or private research and development initiatives (23).
**Lack of or insufficient technical advice or technical support for land managers/ operators**	• Facilitate local ecological knowledge through peer knowledge transfer, on-side demonstrations, and seed exchange networks (45).<br>• Create dedicated forestry extension unit to improve coordination and funding of forestry extension programs and provide training for extension officers (46).<br>• Employ boundary spanners to facilitate knowledge transfer between scientists and practitioners and create accessible research syntheses (47).
**Limited land manager/ operator literacy, numeracy, or technological capabilities**	• Host annual events that include workshops to improve entrepreneurship skills (48).<br>• Improve technical skills of forest landowners, especially those who have historically been excluded from land ownership and training, to facilitate shifts to technical forestry (49).
**Inadequate planning or management**	• Create spatial management plans to strategically select reforestation locations that align with intended outcomes (50).<br>• Create and implement proactive strategies prior to extreme event occurrence (51).<br>• Empower local administrative levels to make decisions relevant to local contexts instead of centralized national decision-making (52).
**Social & Behavioral Constraints**	
**Preferences or norms favoring non-NCS land uses**	• Establish long-term extension services to shift farming norms (53).<br>• Improve financial stability for rural households by ensuring fair prices for farm products and strengthening economic alternatives that align with sustainable land use (54).<br>• Leverage social networks and respected community figures to promote NCS (55)
**Disinterest or skepticism of NCS or lack of trust toward NCS promoters**	• Build cross-party consensus to depoliticize NCS, ensuring long-term policy stability beyond election cycles (56).<br>• Foster trust between NCS promoters and land users by demonstrating long-term commitment (57).<br>• Demonstrate tangible benefits of NCS through pilot projects, field demonstrations, and farmer-to-farmer knowledge sharing (45).<br>• Design restoration projects with aesthetic and practical benefits to increase local approval and attract developers (56).
**Limited social learning or exchange networks**	• Support traditional knowledge preservation by integrating local ecological knowledge into formal education, demonstrations, and restoration initiatives (27).<br>• Integrate local knowledge into research and monitoring efforts (58).<br>• Strengthen farmer-to-farmer learning networks through peer exchanges, on-site demonstrations, and seed exchange programs to facilitate knowledge transfer (45).5/23/25 2:40:00 AM
**Lack of opportunity to participate in or influence**	• Support Indigenous-led conservation by adapting policies to uphold Indigenous rights, investing in cultural preservation, and integrating traditional governance structures into environmental programs (59).<br>• Strengthen gender equality by establishing female-led farmer organizations with support from extension agents (60).

**implementation due to gender, race, ethnicity, or other dimensions of identity**	• Encourage participation of underrepresented groups in conservation enterprises by establishing training programs, leadership opportunities, and demographic representation in governance bodies (61).
**Interpersonal conflict**	• Formalize property management structures within families to prevent inheritance-related disputes by designating a property administrator or hiring mediation services (49).<br>• Encourage cooperative agreements among landowners to share responsibilities and benefits of conservation practices (35).<br>• Strengthening stakeholder engagement to integrate diverse values from the outset and use boundary spanners to translate scientific knowledge (47).
**Human-wildlife conflict**	• Provide timely financial assistance to farmers facing wildlife-related losses and establish clear thresholds for acceptable damage (62).<br>• Integrate preventative measures such as fencing, wildlife-resistant crops, and strategic land-use planning to reduce conflicts and minimize economic losses for farmers (63).
**Concerns over negative equity impacts**	• Improve local land tenure security (64).<br>• Apply social multi-criteria evaluation (SMCE) to explicitly consider equity and justice in land use planning (65).<br>• Create mechanisms to include poorer and smaller farmers in payment for ecosystem services (PES) schemes, such as local PES programs (66).<br>• Support sustainable livelihoods of forest-dependent communities, such as bamboo furniture and essential oil production (42).
**Policies & Rules Constraints**	
**Ineffective laws, policies, or regulations**	• Revise perverse regulations and reduce taxes and reporting costs for restoration (67).<br>• Update outdated forestry laws, modelled after best practices developed in other countries (68).<br>• Adopt selective logging codes of practice, occupational health and safety standards, and compliance mechanisms to attract professional harvesters with experience in sustainable forestry (69).<br>• Use “smart regulation,” a flexible approach to governance arrangements that considers synergies between stakeholder groups in design and implementation of regulations (70).
**Lack of laws, policies, or regulations**	• Create restoration protocols as two levels of government: a) federal government develop guiding protocol aligned with global restoration policies and practices, and b) local governments develop context-specific protocols within the framework of the federal guidelines (42).<br>• Develop targeted policies agroforestry, which could include establishing livelihoods and recognizing Indigenous knowledge and value systems (34).<br>• Bring together stakeholders from different sectors and scales to develop regulatory frameworks (ex. CONAVEG in Brazil and “Mesa de Restauración” in Guatemala) (71).
**Insecure land tenure**	• Implement a participatory Fit-for-Purpose (FfP) land administration approach to recognize diverse tenure rights and resolve conflicts (72).

	• Frame land tenure disputes as socio-territorial conflicts, emphasizing shared interests among neighboring communities and their interdependence in forest management to foster cooperative solutions and improve tenure security (73).<br>• Design policies that integrate tenure security with non-tenurial factors, such as targeted extension services, to enhance market performance and incentivize the adoption of sustainable land management practices (74).
**Unsecure or uncertain NCS benefit sharing**	• Establish and enforce legal protections for tree tenure to ensure land stewards receive the full benefits of their restoration efforts by preventing unauthorized harvesting (54).<br>• Implement spatially targeted and differentiated payments, prioritizing continuous funding from ecosystem service beneficiaries rather than short-term projects, to create more predictable and equitable NCS benefit-sharing mechanisms (75).<br>• Secure legal rights for smallholders to harvest and benefit from planted trees (76).
**Incentives for non-NCS land use**	• Reform agricultural policies and subsidies to remove disincentives for tree planting, shifting farming culture away from viewing trees as obstacles (77).<br>• Enforce supply chain regulations and moratoriums on deforestation-linked soy and meat production to break the cycle of agribusiness-driven deforestation (78).<br>• Establish restoration grants to offset income delays and provide viable economic alternatives to land uses that compete with NCS (17).
**Governments & Organizations Constraints**	
**Lack of administrative capacity for implementation**	• Develop collaborative, structured knowledge-sharing platforms to improve monitoring, reporting, and verification (MRV) (79).<br>• Support local governance and institutional strengthening through multi-stakeholder collaborations and investment in local institutions (80).<br>• Enhance learning networks and knowledge transfer mechanisms (56).
**Lack of enforcement of laws, policies, or regulations**	• Establish collective land titling to create incentives for community vigilance and reduce intrusion (81).<br>• Increase deforestation fine value and quantity (82).<br>• Structure financial mechanisms, such as REDD+ or Voluntary Partnership Agreement/FLEGT, around compliance with forest regulations (83).
**Lack of coordination among organizations**	• Empower bridging organizations, such as farmer associations and local governments, to facilitate collaboration and knowledge-sharing between stakeholders (84).<br>• Establish multi-scale coordination platforms to align international, national, and local policy efforts (85).<br>• Align external monitoring programs with existing local governance structures to prevent confusion and duplication of efforts (86).
**Lack of political will**	• Frame delayed NCS action as a "mitigation debt" that increases future economic costs and welfare losses, creating an economic argument for immediate investment (87).<br>• Implement supply chain interventions, such as soy and meat moratoriums, to create economic pressure for stronger environmental governance and reduce incentives for deforestation (78).

**Violent conflict or civil unrest**	• Develop multi-level MRV systems in conflict-prone regions, involving local communities, government agencies, and NGOs to improve data reliability, legitimacy, and enforcement. Collaborative monitoring can enhance transparency, resolve land-use disputes, and build trust in forest governance (79).
**Corruption or lack of transparency**	• Establish community auditing and special oversight commissions of Community Forest Enterprises to enforce statutes, facilitating trust and preventing corruption (61).<br>• Implementers, donors, and facilitators increase transparency of decision processes and procedural equity through holding participative village workshops, brochures, and informational radio and television programming (75).<br>• Make Forestry Divisions independent so they are not subject to political interference (68).
**Material Inputs Constraints**	
**Lack of input materials or infrastructure**	• Strengthen seed and seedling supply chains by partnering with traditional communities (88).<br>• Promote small-scale mechanization by advancing affordable equipment suited for agroforestry (89).<br>• Scale up wild seed collection by training additional seed collectors and supplementing traditional seed sourcing with advanced propagation techniques (90).
**Lack of labor**	• Support new workforce development through targeted training programs and peer-to-peer learning (27).<br>• Improve planting machines (14).<br>• Adopt collective hiring models to pool resources, centralize skills development, and improve workforce efficiency (91).
**Lack of water or water distribution networks**	• Implement traditional water conservation techniques such as stone bunds, half-moons, zaï pits, soil management, nurse shrubs, and Vallerani trenches to capture rainwater and improve soil moisture (92).<br>• Strategically locate restoration sites near existing wells used for agriculture or other purposes to ensure a reliable water source without the high costs of new drilling (92).
**Unsuitable land**	• Support smallholders in agroforestry adoption by providing access to public land under a legal framework and promoting collective farming initiatives (93).<br>• Adapt NCS approaches to land size constraints by promoting practices that minimize interference with farm operations and provide income (35).<br>• Assess natural regeneration potential before implementation, prioritizing areas near forest reserves with sufficient seed sources (94).
**Negative Side Effects**	
**Tradeoffs with other non-agriculture land uses**	• Integrate traditional cultural land use, traditional knowledge, and cultural values into forest management strategies (95).<br>• Financial incentives to encourage adoption, such as results-based carbon payments or increasing rural credit through loan guarantees or credit subsidies (96).
**Other negative side effects**	• Develop strategic spatial plans tailored to the multiple desired objectives (50).<br>• Reduce carbon emissions from forestry trucks and machinery through using biofuels from biomass and forest residues and high fuel efficiency vehicles (97).<br>• Provide training for farmers as they transition to more technically-challenging practices (98).

Table S4. Agreement in two rounds of intercoder reliability.

	**Agreement %**	
	**Round 1**	**Round 2**
Average	81.3	90.5
≥*50%*	100	100
≥*75%*	85	90
=100%	40	73

Dataset S1 (separate file). Dataset on recent constraints to Natural Climate Solution (NCS) implementation (7). The “FullData” tab is the fully coded dataset resulting from the literature review and used in the analysis in this paper. The “Codebook” tab describes the variables in the “FullData” tab. The “ConstraintDefinitions” tab defines each of the 39 constraints and identifies their corresponding categories. The “PathwayDefinitions” tab defines each of the NCS pathways studied, as well as many of their sub-pathways. The “CountryCodes” tab provides the geographic categorization used in the paper for each country.

SI References

1. B. C. Wallace, K. Small, C. E. Brodley, J. Lau, T. A. Trikalinos, Deploying an interactive machine learning system in an evidence-based practice center: abstrackr in *Proceedings of the 2nd ACM SIGHIT International Health Informatics Symposium*, IHI '12., (Association for Computing Machinery, 2012), pp. 819–824.

2. S. Roe, *et al.*, Land-based measures to mitigate climate change: Potential and feasibility by country. *Global Change Biology* **27**, 6025–6058 (2021).

3. Sixth Assessment Report — IPCC. Available at: https://www.ipcc.ch/assessment-report/ar6/ [Accessed 10 March 2025].

4. I. Schulte, J. Eggers, J. Ø. Nielsen, S. Fuss, What influences the implementation of natural climate solutions? A systematic map and review of the evidence. *Environ. Res. Lett.* **17**, 013002 (2021).

5. L. Karki, *et al.*, Potentials and barriers to land-based mitigation technologies and practices (LMTs)—a review. *Environ. Res. Lett.* **18**, 093003 (2023).

6. AR5 Climate Change 2014: Mitigation of Climate Change — IPCC. Available at: https://www.ipcc.ch/report/ar5/wg3/ [Accessed 10 March 2025].

7. [dataset]* Hilary Brumberg, *et al.*, Global constraints to natural climate solution implementation. Harvard Dataverse, V3. https://doi.org/10.7910/DVN/NEYIPD. Deposited 23 September 2024.

8. United Nations, SDG Indicators. Available at: https://unstats.un.org/sdgs/indicators/regional-groups/ [Accessed 18 September 2024].

9. United Nations Statistics Division, Geographic Regions. Available at: https://unstats.un.org/unsd/methodology/m49/ [Accessed 18 September 2024].

10. Naturebase, All NCS Pathways v1. Deposited 2023.

11. H. Do, E. Luedeling, C. Whitney, Decision analysis of agroforestry options reveals adoption risks for resource-poor farmers. *Agron. Sustain. Dev.* **40**, 1–12 (2020).

12. R. W. Kariuki, *et al.*, Integrating stakeholders' perspectives and spatial modelling to develop scenarios of future land use and land cover change in northern Tanzania. *PLOS ONE* **16**, e0245516 (2021).

13. R. Reyes, H. Nelson, H. Zerriffi, How do decision makers´ ethnicity and religion influence the use of forests? Evidence from Chile. *Forest Policy and Economics* **128**, 102462 (2021).

14. J. Manner, B. T. Ersson, Mechanized tree planting in Nordic forestry: simulating a machine concept for continuously advancing site preparation and planting. *J. For. Sci.* **67**, 242–246 (2021).

15. R. T. Yao, D. J. Palmer, T. W. Payn, S. Strang, C. Maunder, Assessing the Broader Value of Planted Forests to Inform Forest Management Decisions. *Forests* **12**, 662 (2021).

16. R. Crouzeilles, *et al.*, Achieving cost-effective landscape-scale forest restoration through targeted natural regeneration. *Conservation Letters* **13**, e12709 (2020).

17. I. Brown, Challenges in delivering climate change policy through land use targets for afforestation and peatland restoration. *Environmental Science & Policy* **107**, 36–45 (2020).

18. S. Wolf, M. Pham, N. Matthews, P. Bubeck, Understanding the implementation gap: policy-makers' perceptions of ecosystem-based adaptation in Central Vietnam. *Climate and Development* **13**, 81–94 (2021).

19. Z. Ding, S. Yao, Ecological effectiveness of payment for ecosystem services to identify incentive priority areas: Sloping land conversion program in China. *Land Use Policy* **104**, 105350 (2021).

20. J. C. dos Reis, *et al.*, Assessing the economic viability of integrated crop−livestock systems in Mato Grosso, Brazil. *Renewable Agriculture and Food Systems* **35**, 631–642 (2020).

21. V. Owusu, V. Akoto-Adjepong, E. Acheampong, V. R. Barnes, Farmer Perceptions and Economic Performance of Cocoa Agroforestry Shade Levels in Ghana. *Journal of Sustainable Forestry* **41**, 922–940 (2022).

22. E. Elzaki, G. Tian, Economic evaluation of the honey yield from four forest tree species and the future prospect of the forest beekeeping in Sudan. *Agroforest Syst* **94**, 1037–1045 (2020).

23. M. Gastauer, *et al.*, Balancing natural forest regrowth and tree planting to ensure social fairness and compliance with environmental policies. *Journal of Applied Ecology* **58**, 2371–2383 (2021).

24. A. Ahtikoski, R. Ahtikoski, M. Haapanen, J. Hynynen, K. Kärkkäinen, Economic Performance of Genetically Improved Reforestation Material in Joint Production of Timber and Carbon Sequestration: A Case Study from Finland. *Forests* **11**, 847 (2020).

25. A. Golub, D. Herrera, G. Leslie, B. Pietracci, R. Lubowski, A real options framework for reducing emissions from deforestation: Reconciling short-term incentives with long-term benefits from conservation and agricultural intensification. *Ecosystem Services* **49**, 101275 (2021).

26. V. H. Do, *et al.*, Fruit Tree-Based Agroforestry Systems for Smallholder Farmers in Northwest Vietnam—A Quantitative and Qualitative Assessment. *Land* **9**, 451 (2020).

27. C. Barlagne, *et al.*, Stakeholders' engagement platform to identify sustainable pathways for the development of multi-functional agroforestry in Guadeloupe, French West Indies. *Agroforest Syst* **97**, 463–479 (2023).

28. G. Techel, M. Lemenih, T. Pistorius, Planning FLR for the landscape between Chebera Churchura National Park and Kafa Biosphere Reserve. *Land Use Policy* **104**, 104242 (2021).

29. R. Ranjan, Payments for ecosystems services-based agroforestry and groundwater nitrate remediation: The case of *Poplar deltoides* in Uttar Pradesh, India. *Journal of Cleaner Production* **287**, 125059 (2021).

30. B. Spencer, *et al.*, Determinants of the economic viability of mallee eucalypts as a short rotation coppice crop integrated into farming systems of Western Australia. *GCB Bioenergy* **13**, 242–256 (2021).

31. B. D. V. Marino, V. Truong, J. W. Munger, R. Gyimah, Direct measurement forest carbon protocol: a commercial system-of-systems to incentivize forest restoration and management. *PeerJ* **8**, e8891 (2020).

32. B. Chhiev, V. Jongrungrot, Rubber agroforestry system (RAS) practices to overcome rubber price and soil erosion in southern Thailand. *International Journal of Agricultural Technology* **17**, 13–32 (2021).

33. E. Stutzman, R. J. Barlow, W. Morse, D. Monks, L. Teeter, Natural resource professionals' engagement with landowners on silvopasture in the Southeastern United States. *Agroforest Syst* **94**, 2137–2146 (2020).

34. F. I. Alambo, Agroforestry-based Livelihoods in the Face Of Cultural and Socio-economic Dynamics in Rural Gedeo, Southern Ethiopia. *Journal of Rural and Community Development* **15** (2020).

35. D. Ruppert, M. Welp, M. Spies, N. Thevs, Farmers' Perceptions of Tree Shelterbelts on Agricultural Land in Rural Kyrgyzstan. *Sustainability* **12**, 1093 (2020).

36. A. E. B. Lacerda, A. L. Hanisch, E. R. Nimmo, Leveraging Traditional Agroforestry Practices to Support Sustainable and Agrobiodiverse Landscapes in Southern Brazil. *Land* **9**, 176 (2020).

37. P. Waldén, M. Ollikainen, H. Kahiluoto, Carbon revenue in the profitability of agroforestry relative to monocultures. *Agroforest Syst* **94**, 15–28 (2020).

38. P. Campos, *et al.*, Total income and ecosystem service sustainability index: Accounting applications to holm oak *dehesa* case study in Andalusia-Spain. *Land Use Policy* **97**, 104692 (2020).

39. Ö. Grönlund, E. Erlandsson, L. Djupström, D. Bergström, L. Eliasson, Nature conservation management in voluntary set-aside forests in Sweden: practices, incentives and barriers. *Scandinavian Journal of Forest Research* **35**, 96–107 (2020).

40. P. Meli, *et al.*, A global review of past land use, climate, and active vs. passive restoration effects on forest recovery. *PLOS ONE* **12**, e0171368 (2017).

41. J. M. Rey Benayas, *et al.*, Landscape restoration in a mixed agricultural-forest catchment: Planning a buffer strip and hedgerow network in a Chilean biodiversity hotspot. *Ambio* **49**, 310–323 (2020).

42. S. Bhattarai, B. Pant, H. K. Laudari, R. K. Rai, S. A. Mukul, Strategic Pathways to Scale up Forest and Landscape Restoration: Insights from Nepal's Tarai. *Sustainability* **13**, 5237 (2021).

43. M. F. Zerihun, Agroforestry Practices in Livelihood Improvement in the Eastern Cape Province of South Africa. *Sustainability* **13**, 8477 (2021).

44. S. Puliti, A. Granhus, Drone data for decision making in regeneration forests: from raw data to actionable insights1. *J. Unmanned Veh. Sys.* **9**, 45–58 (2021).

45. B. C. Tarbox, M. Swisher, Z. Calle, C. H. Wilson, S. L. Flory, Decline in local ecological knowledge in the Colombian Andes may constrain silvopastoral tree diversity. *Restoration Ecology* **28**, 892–901 (2020).

46. E. I. Agube, E. M. Igbokwe, O. F. Ojo, Role of Extension Forest Officers in Forest Conservation in Cross River State, Nigeria. *Journal of Agricultural Extension* **25**, 69–80 (2021).

47. S. Busbridge, B. D. Clarkson, K. J. Wallace, A tenuous link: Information transfer between urban ecological research and restoration practice. *Urban Forestry & Urban Greening* **60**, 127019 (2021).

48. R. K. Bannor, M. A. F. Ros-Tonen, P. O. Mensah, M. Derkyi, V. F. Nassah, Entrepreneurial behaviour among non-timber forest product-growing farmers in Ghana: An analysis in support of a reforestation policy. *Forest Policy and Economics* **122**, 102331 (2021).

49. J. Schelhas, S. Hitchner, P. Dwivedi, M. Thomas, Understanding black landowner's engagement in forestry in Georgia, United States: a closer look. *Forests, Trees and Livelihoods* **30**, 242–257 (2021).

50. K. von Kleist, J. Herbohn, J. Baynes, N. Gregorio, How improved governance can help achieve the biodiversity conservation goals of the Philippine National Greening Program. *Land Use Policy* **104**, 104312 (2021).

51. S. Orchard, D. R. Schiel, Enabling nature-based solutions for climate change on a peri-urban sandspit in Christchurch, New Zealand. *Reg Environ Change* **21**, 1–18 (2021).

52. M. Valette, *et al.*, Beyond fixes that fail: identifying sustainable improvements to tree seed supply and farmer participation in forest and landscape restoration. *Ecology and Society* **25** (2020).

53. S. Lee, *et al.*, Adoption potentials and barriers of silvopastoral system in Colombia: Case of Cundinamarca region. *Cogent Environmental Science* **6**, 1823632 (2020).

54. T. Kibru, R. Husseini, E. Birhane, J. Haggar, N. Solomon, Farmers' perception and reasons for practicing farmer managed natural regeneration in Tigray, Ethiopia. *Agroforest Syst* **95**, 1327–1342 (2021).

55. M. K. Lapinski, *et al.*, Culture and social norms: Behavioral decisions about grassland conservation among ethnically Tibetan pastoralists. *Journal of International and Intercultural Communication* **15**, 333–354 (2022).

56. S. Han, C. Kuhlicke, Barriers and Drivers for Mainstreaming Nature-Based Solutions for Flood Risks: The Case of South Korea. *Int J Disaster Risk Sci* **12**, 661–672 (2021).

57. T. M. Gladkikh, J. A. Collazo, A. Torres-Abreu, A. M. Reyes, M. Molina, Factors that influence participation of Puerto Rican coffee farmers in conservation programs. *Conservation Science and Practice* **2**, e172 (2020).

58. S. W. Lyon, M. DiBlasio, E. Creveling, On using initial monitoring data to communicate restoration potentials and limitations. *Applied Environmental Education & Communication* **19**, 287–302 (2020).

59. B. Villhauer, Transforming REDD+ for Indigenous rights in Costa Rica. *Local Environment* **26**, 1221–1234 (2021).

60. C. Duffy, G. Toth, J. Cullinan, U. Murray, C. Spillane, Climate smart agriculture extension: gender disparities in agroforestry knowledge acquisition. *Climate and Development* **13**, 21–33 (2021).

61. M. Butler, D. Current, A Comparative Analysis of Community-Based Enterprise Governance in the Maya Biosphere Reserve. *Society & Natural Resources* **34**, 1449–1471 (2021).

62. M. Puri, E. Pienaar, K. Karanth, B. Loiselle, Food for thought—examining farmers' willingness to engage in conservation stewardship around a protected area in central India. *Ecology and Society* **26** (2021).

63. H. Yang, F. Lupi, J. Zhang, J. Liu, Hidden cost of conservation: A demonstration using losses from human-wildlife conflicts under a payments for ecosystem services program. *Ecological Economics* **169**, 106462 (2020).

64. A. van der Meer Simo, P. Kanowski, K. Barney, The role of agroforestry in swidden transitions: a case study in the context of customary land tenure in Central Lao PDR. *Agroforest Syst* **94**, 1929–1944 (2020).

65. E. Zepharovich, M. G. Ceddia, S. Rist, Social multi-criteria evaluation of land-use scenarios in the Chaco Salteño: Complementing the three-pillar sustainability approach with environmental justice. *Land Use Policy* **101**, 105175 (2021).

66. K. Brownson, *et al.*, Governance of Payments for Ecosystem Ecosystem services influences social and environmental outcomes in Costa Rica. *Ecological Economics* **174**, 106659 (2020).

67. R. D. Harrison, *et al.*, Restoration concessions: a second lease on life for beleaguered tropical forests? *Frontiers in Ecology and the Environment* **18**, 567–575 (2020).

68. M. Fayiah, Uncertainties and trends in the forest policy framework in Sierra Leone: an overview of forest sustainability challenges in the post-independence era. *International Forestry Review* **23**, 139–150 (2021).

69. B. H. Poudyal, T. Maraseni, G. Cockfield, An assessment of the policies and practices of selective logging and timber utilisation: A case study from natural forests of *Tarai* Nepal and Queensland Australia. *Land Use Policy* **91**, 104422 (2020).

70. H. Smith, P. Kanowski, R. J. Keenan, S. Phimmavong, Lao Plantation Policy: Prospects for Change. *Forests* **12**, 1132 (2021).

71. D. Schweizer, P. Meli, P. H. S. Brancalion, M. R. Guariguata, Implementing forest landscape restoration in Latin America: Stakeholder perceptions on legal frameworks. *Land Use Policy* **104**, 104244 (2021).

72. B. Reydon, M. Molendijk, N. Porras, G. Siqueira, The Amazon Forest Preservation by Clarifying Property Rights and Potential Conflicts: How Experiments Using Fit-for-Purpose Can Help. *Land* **10**, 225 (2021).

73. V. Gutiérrez-Zamora, M. Hernández Estrada, Responsibilization and state territorialization: Governing socio-territorial conflicts in community forestry in Mexico. *Forest Policy and Economics* **116**, 102188 (2020).

74. F. A. Asaaga, M. A. Hirons, Y. Malhi, Questioning the link between tenure security and sustainable land management in cocoa landscapes in Ghana. *World Development* **130**, 104913 (2020).

75. J. G. Montoya-Zumaeta, S. Wunder, L. Tacconi, Incentive-based conservation in Peru: Assessing the state of six ongoing PES and REDD+ initiatives. *Land Use Policy* **108**, 105514 (2021).

76. N. Sibelet, C. A. Gómez-Luciano, I. A. Gutiérrez-Montes, A Success Story of Collective Action Changing Public Policy: Farmers' Innovation in the Face of Repressive Forestry Policy in Zambrana Abajo, Dominican Republic. *Small-scale Forestry* **20**, 351–369 (2021).

77. O. FitzGerald, C. M. Collins, C. Potter, Woodland Expansion in Upland National Parks: An Analysis of Stakeholder Views and Understanding in the Dartmoor National Park, UK. *Land* **10**, 270 (2021).

78. E. J. de Area Leão Pereira, L. C. de Santana Ribeiro, L. F. da Silva Freitas, H. B. de Barros Pereira, Brazilian policy and agribusiness damage the Amazon rainforest. *Land Use Policy* **92**, 104491 (2020).

79. R. Sufo Kankeu, M. Tsayem Demaze, M. Krott, D. J. Sonwa, S. Ongolo, Reprint of "Governing knowledge transfer for deforestation monitoring: Insights from REDD+ projects in the Congo Basin region." *Forest Policy and Economics* **114**, 102105 (2020).

80. C. Futemma, F. De Castro, E. S. Brondizio, Farmers and Social Innovations in Rural Development: Collaborative Arrangements in Eastern Brazilian Amazon. *Land Use Policy* **99**, 104999 (2020).

81. M. A. Vélez, J. Robalino, J. C. Cardenas, A. Paz, E. Pacay, Is collective titling enough to protect forests? Evidence from Afro-descendant communities in the Colombian Pacific region. *World Development* **128**, 104837 (2020).

82. M. B. Saraiva, *et al.*, Forest regeneration in the Brazilian Amazon: Public policies and economic conditions. *Journal of Cleaner Production* **269**, 122424 (2020).

83. F. Ankomah, K. Boateng, W. Asante, M. Ansong, Implementation gaps in forest management prescriptions and noncompliance in forest regulations in Ghana: Case study of four forest reserves. *Environ Dev Sustain* **24**, 9355–9379 (2022).

84. Y. Zinngrebe, *et al.*, Agroforestry governance for operationalising the landscape approach: connecting conservation and farming actors. *Sustain Sci* **15**, 1417–1434 (2020).

85. R. F. Velasco, M. Köthke, M. Lippe, S. Günter, Scale and context dependency of deforestation drivers: Insights from spatial econometrics in the tropics. *PLOS ONE* **15**, e0226830 (2020).

86. L. F. Kowler, *et al.*, Aiming for Sustainability and Scalability: Community Engagement in Forest Payment Schemes. *Forests* **11**, 444 (2020).

87. K. Glenk, M. Faccioli, J. Martin-Ortega, C. Schulze, J. Potts, The opportunity cost of delaying climate action: Peatland restoration and resilience to climate change. *Global Environmental Change* **70**, 102323 (2021).

88. W. B. R. Martins, *et al.*, Mining in the Amazon: Importance, impacts, and challenges to restore degraded ecosystems. Are we on the right way? *Ecological Engineering* **174**, 106468 (2022).

89. S. B. Brodt, N. M. Fontana, L. F. Archer, Feasibility and sustainability of agroforestry in temperate industrialized agriculture: preliminary insights from California. *Renewable Agriculture and Food Systems* **35**, 513–521 (2020).

90. J. Fargione, *et al.*, Challenges to the Reforestation Pipeline in the United States. *Front. For. Glob. Change* **4** (2021).

91. F. Brandão, G. Schoneveld, Oil Palm Contract Farming in Brazil: Labour Constraints and Inclusivity Challenges. *The Journal of Development Studies* **57**, 1428–1442 (2021).

92. B. Vinceti, M. Valette, A. L. Bougma, A. Turillazzi, How Is Forest Landscape Restoration Being Implemented in Burkina Faso? Overview of Ongoing Initiatives. *Sustainability* **12**, 10430 (2020).

93. A. Dhakal, R. K. Rai, Who Adopts Agroforestry in a Subsistence Economy?—Lessons from the Terai of Nepal. *Forests* **11**, 565 (2020).

94. M. Lohbeck, *et al.*, Drivers of farmer-managed natural regeneration in the Sahel. Lessons for restoration. *Sci Rep* **10**, 15038 (2020).

95. J. Jaworek-Jakubska, M. Filipiak, A. Napierała-Filipiak, Understanding of Forest Cover Dynamics in Traditional Landscapes: Mapping Trajectories of Changes in Mountain Territories (1824–2016), on the Example of Jeleniogórska Basin, Poland. *Forests* **11**, 867 (2020).

96. A. Wilkes, S. Wang, L. Lipper, X. Chang, Market Costs and Financing Options for Grassland Carbon Sequestration: Empirical and Modelling Evidence From Qinghai, China in *Frontiers in Environmental Science*, (2021), p. 657608.

97. G. Medeiros, T. Florindo, E. Talamini, A. Fett Neto, C. Ruviaro, Optimising Tree Plantation Land Use in Brazil by Analysing Trade-Offs between Economic and Environmental Factors Using Multi-Objective Programming. *Forests* **11**, 723 (2020).

98. A. Sharmin, M. Hossain, A. S. Mollick, Farmers' Perceptions and Attitudes Toward Aquasilviculture in the Periphery of the Sundarbans Forest of Bangladesh. *Small-scale Forestry* **20**, 391–405 (2021).
